# Soil Seed Bank Persistence Across Time and Burial Depth in Calcareous Grassland Habitats

**DOI:** 10.3389/fpls.2021.790867

**Published:** 2022-02-04

**Authors:** Tereza Mašková, Peter Poschlod

**Affiliations:** Ecology and Conservation Biology, Institute of Plant Sciences, University of Regensburg, Regensburg, Germany

**Keywords:** calcareous grasslands, dormancy, longevity index, soil seed bank, light requirements for germination, alternating temperature requirements for germination, burial depth

## Abstract

Seed persistence in the soil is crucial for population dynamics. Interspecific differences in soil seed mortality could be a mechanism that may stimulate species coexistence in herbaceous plant communities. Therefore, understanding the levels and causes of seed persistence is vital for understanding community composition and population dynamics. In this study, we evaluated the burial depth as a significant predictor of the temporal dynamics of soil seed persistence. We suppose that species differ in this temporal dynamics of soil seed persistence according to burial depth. Furthermore, we expected that burial depth would affect soil seed persistence differently concerning the species-specific type of dormancy, light, and fluctuating temperature requirements for germination. Seeds of 28 herbaceous species of calcareous grasslands were buried in the field into depths of 1, 5, and 10 cm under the soil surface. Seed viability was tested by germination and tetrazolium tests several times for three years. Species-specific seed traits—a type of dormancy, light requirements and alternating temperature requirements for germination, and longevity index—were used for disentangling the links behind species-specific differences in soil seed persistence. Our study showed differences in soil seed persistence according to the burial depth at the interspecific level. Generally, the deeper the buried seeds, the longer they stayed viable, but huge differences were found between individual species. Species-specific seed traits seem to be an essential determinant of seed persistence in the soil. Seeds of dormant species survived less and only dormant seeds stayed viable in the soil. Similarly, seeds of species without light or alternating temperature requirements for germination generally remained viable in the soil in smaller numbers. Moreover, seeds of species that require light for germination stayed viable longer in the deeper soil layers. Our results help understand the ecosystem dynamics caused by seed reproduction and highlight the importance of a detailed long-term investigation of soil seed persistence. That is essential for understanding the fundamental ecological processes and could help restore valuable calcareous grassland habitats.

## Introduction

Soil seed bank formation is one of the bed-hedging mechanisms for many species because more or less part of their seeds stay ungerminated in the soil for the future years (Venable and Brown, 1988), especially in ecosystems where opportunities for seedling establishment are unpredictable (Wijayratne and Pyke, 2012; Bhatt et al., 2019). At the same time, species-specific differences in the soil seed persistence are known to promote species diversity and co-existence in herbaceous plant communities, as referred to in the storage effect concept (Chesson and Warner, 1981; Warner and Chesson, 1985; Facelli et al., 2005). Understanding the soil seed bank persistence is crucial for understanding the community dynamics in both short- and long-term perspectives (Basto et al., 2018), can improve predictions of species distribution under a changing environment (Ooi, 2012), and has direct implications for successful management and restoration of endangered ecosystems (Richter and Stromberg, 2005; Tatár, 2010; Chapman et al., 2019; Funk, 2021).

One widely used method to study soil seed persistence is determining the depth distribution of germinable seeds in the soil. The depth distribution of seeds is considered indirect evidence of seed longevity (Thompson, 1993; Thompson et al., 1997). Based on these measurements, Thompson et al. (1998) classified soil seed persistence as “transient” or “persistent” and calculated the longevity index to summarize seed-soil persistence data from different studies. On the other hand, direct evidence of seed longevity (i.e., the time elapsed since a species was the last present on the locality or long-term field burial experiments) is rarely used (but see Schwienbacher et al., 2010; Moravcová et al., 2018), although it is known that site-specific conditions can affect both the soil seed persistence (Schafer and Kotanen, 2003; Long et al., 2015) and burial depth (Benvenuti, 2007; Egawa and Tsuyuzaki, 2013). Furthermore, results may be confounded by seed size—seed number trade-off. Smaller seeds enter easier deeper soil layers (Benvenuti, 2007) and are often overrated according to their persistence than larger seeds during indirect longevity estimation (Saatkamp et al., 2009).

Moreover, the deeper the seeds are buried, the lower the chance of seedling reaching the surface (Pearson et al., 2002; Grundy et al., 2003). Different mechanisms which allow seeds to stay ungerminated in the deeper soil layers were evolved (Milberg et al., 2000; El-Keblawy et al., 2018). Three main aspects of germination traits are considered the most important for soil seed persistence, germination timing, and hitting the gap of favorable conditions after disturbances or during the season (Grubb, 1977; Fenner and Thompson, 2005; Saatkamp et al., 2011b). First, delayed germination *via* dormancy mechanisms (Baskin and Baskin, 2014). Second, light requirements for germination, since light can penetrate only an upper layer of soil (Kasperbauer and Hunt, 1988; Mandoli et al., 1990), so seeds can persist deeper until disturbances occur (Baskin and Baskin, 2014; Milberg et al., 2000). Third, alternating temperature requirements for germination may also serve as detection of burial depth and for a gap detection (Thompson and Grime, 1983).

It is unclear whether and how burial depth affects soil seed persistence during a time, and comparison among a higher number of species is almost missing (but see Rivera et al., 2012). Therefore, we investigated the soil seed persistence concerning the burial depth. We address these hypotheses: (i) the deeper the seeds are buried, the longer they will remain viable, (ii) seeds of non-dormant species will survive shorter in the soil without respect to the burial depth, and (iii) seeds of species with light and/or alternating temperature requirements for germination will survive in higher proportions in deeper layers where light is not available and temperature conditions are more constant.

## Materials and Methods

### Study Site and Species Selection

The experiment was located in Baden-Wuerttemberg, Germany. We chose four localities of calcareous grassland—Teck (48.59N, 9.47E), Eichhalde (48.58N, 9.49E), Eselsrain (48.51N, 9.06E), and Surrlesrain (48.84N, 9.05E). All localities are situated on White Jurassic rubble with rendzina soil type. Mild and dry climate and species-rich vegetation are typical. We selected 28 species concerning germinability (Beier, 1991) and aspects of seed-soil bank dynamics (Poschlod and Jackel, 1993). We collected seeds and performed burial experiments between June 1991 and October 1996 (see Table 1 for details about species, localities, and experiment timing).

**TABLE 1:** List of species used in the burial experiment.

Species	Grassland name	Date of burial	Excavation dates	Type of dormancy	Light requirements for germination	Alternating temperature requirements	Longevity index
*Antennaria dioica*	Eichhalde	1993	O 93–A 94– A 95–A 96	PD	NO	NO	0
*Anthericum ramosum*	Teck	1992	O 91–A 92–O 92	PY	NO	YES	0
*Aster amellus*	Surrlesrain	1993	A 94–O 94	ND	NO	NO	0
*Brachypodium pinnatum*	Teck	1991	O 91–A 92–O 92	ND	NO	NO	0.07
*Bromus erectus*	Teck	1991	O 91–A 92–A 92	ND	NO	NO	0.29
*Bupleurum falcatum*	Surrlesrain	1993	A 94–O 94	MD	YES	NO	0
*Carex flacca*	Teck	1991	O 91–A 92–O 92	PD	YES	YES	0.58
*Carlina acaulis*	Teck	1991	A 92	ND	NO	NO	0
*Carlina vulgaris*	Teck	1991	A 92–O 92	ND	NO	NO	0.13
*Cirsium acaule*	Teck	1991	O 91–A 92–O 92	ND	YES	NO	0
*Daucus carota*	Teck	1991	O 91–A 92–O 92	PD	YES	NO	0.73
*Dianthus cartusianorum*	Surrlesrain	1993	A 94–O 94	ND	NO	NO	0
*Festuca ovina*	Teck	1991	O 91–A 92–O 92	ND	NO	NO	0.19
*Gentianella germanica*	Eichhalde	1992	A 93–O 93–A 94	MPD	YES	NO	0.17
*Globularia elongata*	Eichhalde	1993	O 93–A 94–A 95–A 96	PD	YES	YES	0
*Hippocrepis comosa*	Teck	1991	O 91–A 92–O 92	PY	NO	NO	0.22
*Hypericum perforatum*	Eselsrain	1992	O 92–A 93–O 94	ND	YES	NO	0.84
*Lactuca serriola*	Eselsrain	1992	O 92–A 93–A 94	ND	YES	NO	0.88
*Leontodon hispidus*	Teck	1991 (1993)	O 91–A 92–O 92 (A 94–O 94)	ND	YES	NO	0.36
*Linum catharticum*	Teck	1991	O 91–A 92–O 92	PD	YES	NO	0.77
*Lotus corniculatus*	Teck	1991	O 91–A 92–O 92	PY	NO	NO	0.4
*Ononis spinosa*	Teck	1991	O 91–A 92–O 92	PY	NO	NO	0
*Origanum vulgare*	Eselsrain	1992	O 92–A 93–A 94	ND	YES	NO	0.81
*Pimpinella saxifraga*	Teck	1991	O 91–A 92–O 92	MPD	NO	NO	0.05
*Pulsatilla vulgaris*	Eselsrain	1994	O 94	ND	NO	NO	0.33
*Rhinanthus alectorolophus*	Surrlesrain	1993	A 94–O 94	PD	NO	NO	0.67
*Sanguisorba minor*	Teck	1991	O 91–A 92–O 92	ND	NO	NO	0.42
*Sedum reflexum*	Surrlesrain	1993	A 94–O 94	PD	YES	NO	0

*Grassland name = locality of burial; Date of burial indicates the year of seed collection and their immediate burial; Excavation dates indicate time sequence of excavation (A = April in the given year, O = October in the given year); Type of dormancy: MD, morphological dormancy; MPD, morphophysiological dormancy; ND, no dormancy; PD, physiological dormancy; PY, physical dormancy.*

### Burial Experiment

For the burial experiment, the homogeneous site at each of the localities was selected. We collected diaspores at the same locality where the burial experiment was performed. Harvest was timed to the moment of full maturity, i.e., it was possible to separate seeds from the mother plant with a light touch (except for *Carlina* sp., whose entire inflorescences were collected in mid-October before the achenes were blown off). Random selection of harvested seeds and inflorescences was made to obtain the broadest possible natural spectrum of diaspores (Maas, 1989); maternal effects on the dominant structure of diaspores were not taken into account (Gutterman, 1992). Immediately after harvest, we placed 50 seeds into nylon bags (4 cm × 4 cm, mesh size 300 μm). We made a borehole 10 cm deep and put three nylon bags with seeds of the same species inside the soil core in the depth of 1, 5, and 10 cm. We placed the soil core into a nylon bag (mesh size 2 mm) and inserted it back to the soil. We made five replicates for each species for each excavation time (except for *Festuca ovina*, *Cirsium acaule*, and *Anthericum ramosum* with two, two, and four replicates, respectively because there were not enough seeds available for these species). The position of replicates was randomized inside the site.

We performed several excavations of seeds during the next few years. The first excavation took place at the end of October in the same year as seeds were buried (except for late species whose seeds ripen during September or later). The subsequent excavation was performed during April following the burial (after winter freezing), then during October (after one whole season). The remaining replicates were excavated during April in the following years (see Table 1 for details).

We took all seeds which were not germinated or molded in the soil, treated them by 2% solution of sodium hypochlorite for 2 min, placed them in the Petri dish with filter paper and sufficient moisture, and kept them in the growing chamber (22°C/14°C at 14 h/10 h light/dark) for 6 weeks. We counted as viable those in which the radicle emerged through the seed testa. We stratified ungerminated seeds in dark conditions at a temperature of 3°C for 6 weeks and then put them again in the growing chamber with the same settings for the next 6 weeks. We tested the viability of remaining ungerminated seeds using the tetrazolium test to distinguish between viable (but dormant) and death seeds.

### Seed Traits

We used the longevity index from the LEDA database (Kleyer et al., 2008; Poschlod et al., 2020; unpublished data) and information about the type of dormancy, light requirements for germination, and alternating temperature requirements from literature (Kawatani et al., 1976; Grime et al., 1981; Thompson and Grime, 1983; Jones and Turkington, 1986; Pegtel, 1988; Maas, 1989; Beier, 1991; Milberg, 1994; Poschlod et al., 2003; McDavid, 2012; ten Brink et al., 2013; Baskin and Baskin, 2014; Lang et al., 2014; Tudela-Isanta et al., 2018; Leipold et al., 2019; Lopez del Egido et al., 2019; Rosbakh et al., 2020; Holländer and Jäger, n.d.) and online databases ENSCOBASE,^1^ Seed Information Database^2^ (see Table 1 for details).

### Data Analysis

We used a set of mixed-effect linear models with the proportion of viable seeds (all viable seeds regardless of dormancy) and the proportion of dormant seeds (seeds viable according to the tetrazolium test) as the dependent variable. We used species and plant families as random effects. To avoid the model overfitting, we performed individual analysis for each seed trait and used time, burial depth, one of the seed traits (longevity index, dormancy, light requirements, and alternating temperature requirements), and interaction of respective seed trait with time and burial depth as fixed effects. Furthermore, we performed a model with time, burial depth, and its interaction as fixed effects for investigation of the time × depth interaction. Explanatory variables were standardized and log-transformed to meet the assumptions of normality and homogeneity of variance and take into account the right-skewed distribution of these variables. We classified species with the morphological, morphophysiological, physiological, and physical types of dormancy together as dormant species. Mixed-effect models were performed using the lmer function in R package lme4 (Bates et al., 2015). We tested the random effects using the ranova function from the lmerTest package. We calculated *R*^2^ using Nakagawa and Schielzeth’s R^2^_GLMM_ (Johnson, 2014) as implemented in the r.squaredGLMM function from the R package MuMIn. For better understanding the behavior of individual species and at the same time for not to overparametrize the model, we preferred to redo the analysis for each species individually. We fitted linear models for individual species for burial depth, time, and interaction and performed a multiway ANOVA. We used R software (R Core Team, 2021) for performing all analyses.

## Results

### Time and Burial Depth

**FIGURE 1:**
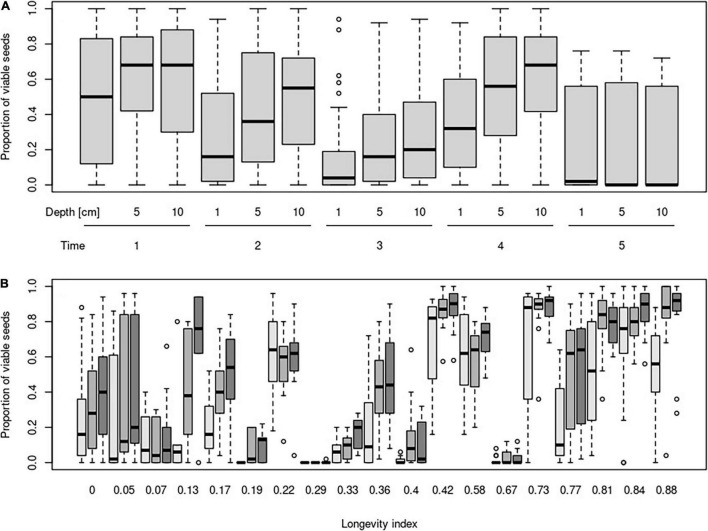
Proportion of viable seeds in different burial depth and their changes according to time **(A)** and longevity index **(B)**. Time steps indicate: 1—October in the same year as seeds were buried; 2—April of the year following the burial; 3—October of the year following the burial; 4—April of the second year following the burial; and 5—April of the third year following the burial. Darkness of boxes indicates burial depth (light gray = 1 cm, middle gray = 5 cm, and dark gray = 10 cm).

The mixed-effect model indicated no very strong main effect of burial depth or time on the proportion of viable seeds. Unsurprisingly, the proportion of viable seeds decreased during the time and increased with burial depth. No significant interaction between time and burial depth was detected (Table 2 and Figure 1A).

**TABLE 2:** Results of set of mixed-effect linear models with the proportion of viable seeds and proportion of dormant seeds as the dependent variable.

Factor	The proportion of viable seeds		The proportion of dormant seeds	
	Estimate	*R* ^2^	Estimate	*R* ^2^
Time	−0.28	0.08***	−0.06	0.002*
Depth	0.05	0.04***	−0.003	–
Time:depth	0.001	–	0.004	–
Longevity index	1.21	0.11***	−0.51	–
Dormancy	−0.34	0.11***	0.86	0.19*
Light requirements	0.82	0.21***	0.1	0.003*
Alternating temperature	0.79	0.13***	0.22	–
Longevity index:time	−0.29	0.16***	0.018	–
Longevity index:depth	0.1	0.03***	0.003	–
Dormancy:time	0.18	0.06***	0.02	–
Dormancy:depth	−0.04	0.04***	−0.002	–
Light requirements:time	0.02	0.05***	−0.03	0.003**
Light requirements:depth	0.0	0.05***	0.002	–
Alternating temperature:time	−0.19	0.06***	−0.02	0.001*
Alternating temperature:depth	0.05	0.04***	0.007	–

Explanatory variables were standardized and log-transformed. Species identity and plant family were included as random effects—indicates non-significant relationships. * indicates significant relationships.

We found only a very low negative effect of time on the proportion of dormant seeds; all other investigated factors—burial depth and interaction between time and burial depth—did not affect the proportion of dormant seeds (Table 2).

For random effects, we found a strong significant effect of species on the proportion of viable seeds (*p* < 0.001), indicating that the interspecific differences are the most important for the soil seed persistence. We found no effect of a family (*p* = 0.14) on the proportion of viable seeds. On the other hand, both random effects—species and family—affected the proportion of dormant seeds (*p* < 0.001, *p* = 0.026, respectively).

### Seed Traits

**FIGURE 2:**
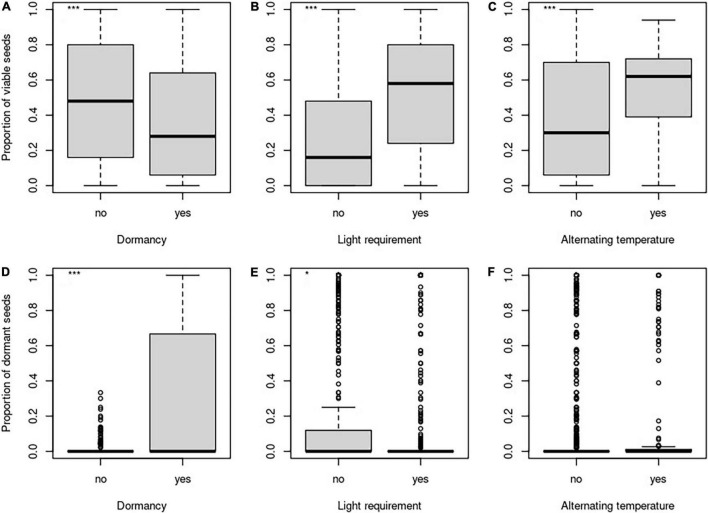
Differences in the proportion of viable seeds **(A–C)** and the proportion of dormant seeds **(D–F)** concerning the dormancy **(A,D)**, light requirements for germination **(B,E)**, and alternating temperature requirements **(C,F)**. *Indicates significant relationship.

**FIGURE 3:**
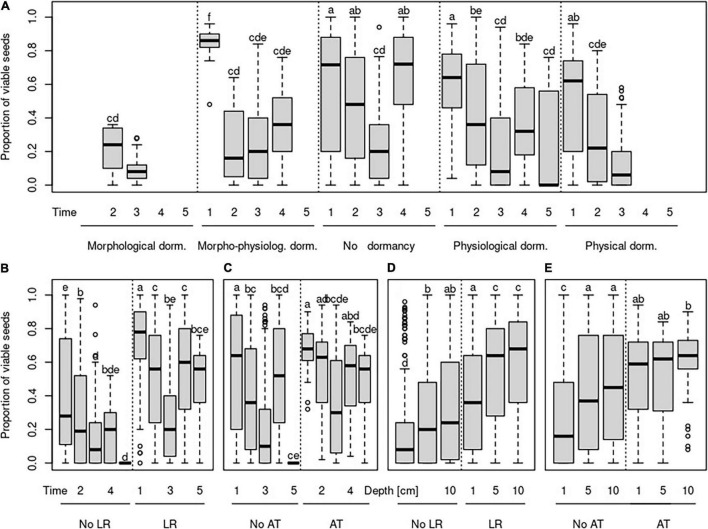
Changes in the proportion of viable seeds over time **(A–C)** and over burial depth **(D,E)** concerning the species-specific type of dormancy **(A)**, species-specific germination requirements to light **(B,D)**, and species-specific germination requirements to alternating temperature **(C,E)**. Time steps indicate: 1—October in the same year as seeds were buried; 2—April of the year following the burial; 3—October of the year following the burial; 4—April of the second year following the burial; 5—April of the third year following the burial. No LR—species without light requirements for germination, LR—species with requirements for germination, No AT—species without alternating temperature requirements for germination, and AT—species with alternating requirements for germination.

The proportion of viable seeds was significantly related to all investigated seed traits—longevity index, dormancy, light requirements for germination, and alternating temperature requirements for germination and interacted with both time and burial depth (Table 2 and Figures 1B, 2 for details). The proportion of dormant seeds was significantly related to the dormancy but time and burial depth did not modify it. Furthermore, we found a significant interaction between time and light requirement for germination and time and alternating temperature requirement for germination in the case of the proportion of dormant seeds. Namely, species with some type of dormancy showed a lower proportion of viable seeds than species without dormancy. We determined the opposite pattern in the case of the proportion of dormant seeds (Figure 3A).

Surprisingly, a significant negative relationship between the proportion of viable seeds and interaction between longevity index and time was detected. The relationship of the proportion of viable seeds with the interaction between longevity index and burial depth was significantly positive.

The species without the requirements to both—light and alternating temperature for germination—had a generally lower proportion of viable seeds (Figures 3B–E). Significant interactions between light requirements for germination with both time and burial depth, on the proportion of viable seeds, showed that seeds of species that require light for germination stayed viable longer in the deeper layer of soil (Figures 3B,D). We also found a significant interaction between alternating temperature requirements and time and burial depth on the proportion of viable seeds. The proportion of viable seeds increased with the burial depth for species with the requirements of alternating temperature and decreased over time for this species (Figure 3E).

### Species-Specific Behavior

For most species (21 from 28), the proportion of viable seeds significantly decreased during the time (e.g., *Brachypodium pinnatum*). We also found the relationship between the proportion of viable seeds and the burial depth—the proportion of viable seeds decreased with the burial depth for four species (e.g., *Origanum vulgare*) and increased for 14 species (e.g., *Gentianella germanica*). There was a significant positive interaction between burial depth and time for three species (*Daucus carota*, *Hypericum perforatum*, and *O. vulgare*) and a significant negative interaction for one species (*Bupleurum falcatum*). See Table 3 and Supplementary Figure 1 for species-specific details.

**TABLE 3:** Results of linear models for individual species between the proportion of viable seeds and proportion of dormant seeds and burial depth, time, and its interaction, performed as a multiway ANOVA.

Species	Proportion of viable seeds			Proportion of dormant seeds		
	depth	time	depth:time	depth	time	depth:time
*Antennaria dioica*	0.03	−0.06				
*Anthericum ramosum*	0.01	−0.38		−0.06		0.05
*Aster amellus*	0.03	−0.10				
*Brachypodium pinnatum*		−0.23				
*Bromus erectus*						
*Bupleurum falcatum*	0.05	−0.03	−0.01			
*Carex flacca*		−0.10				
*Carlina acaulis*						
*Carlina vulgaris*		0.16				
*Cirsium acaule*						
*Daucus carota*	−0.02	−0.28	0.02			
*Dianthus cartusianorum*	0.10	−0.06				
*Festuca ovina*	0.02	−0.03				
*Gentianella germanica*	0.05				−0.45	
*Globularia elongata*		−0.05				
*Hippocrepis comosa*		−0.26				
*Hypericum perforatum*	−0.01	−0.12	0.01			
*Lactuca serriola*	0.05					
*Leontodon hispidus*	0.04	−0.19				
*Linum catharticum*	0.07	−0.10			0.09	
*Lotus corniculatus*	0.02	−0.04				
*Ononis spinosa*	0.05	−0.05				
*Origanum vulgare*	−0.01	−0.15	0.02			
*Pimpinella saxifraga*		−0.38			−0.01	
*Pulsatilla vulgaris*	0.01					
*Rhinanthus alectorolophus*						
*Sanguisorba minor*	−0.01	−0.26				
*Sedum reflexum*	0.01	−0.11			−0.39	

*Numbers indicate an estimate of a given relationship; only significant relationships are shown.*

Only for *A. ramosum* (the only non-legume species with physical dormancy in our dataset), we found a significant relationship between the proportion of dormant seeds and the burial depth and its significant interaction with time—the proportion of dormant seeds declined during the time in the upper layer but increased in the deeper layer. For three species (*Pimpinella saxifraga*, *G. germanica*, and *Sedum reflecum*), the proportion of dormant seeds significantly decreased during the time (e.g., *G. germanica*) and for *Linum catharcticum* significantly increased during the time. All these four species have the physiological or morphophysiological types of dormancy—however, another five species with the physiological type of dormancy did not show this pattern. See Table 3 and Supplementary Figure 2 for species-specific details.

## Discussion

Our burial experiment with seeds of 28 species from calcareous grassland habitat demonstrates the complexity of the soil seed bank and clearly shows that results of indirect investigation of seed longevity have to be interpreted carefully. Although we found significantly better seed persistence in deeper soil layers for species with a higher longevity index, this correlation was not strong. Our findings confirm the previous investigation of Saatkamp et al. (2009) which found no relationship between soil seed persistence in the burial experiment and seed bank persistence and therefore recommended different use of soil seed abundance and experimental soil seed persistence. Moreover, site-specific conditions, such as rainfall or soil texture, affected both natural seed vertical movement (Benvenuti, 2007; Egawa and Tsuyuzaki, 2013) and soil seed persistence (Schafer and Kotanen, 2003; Long et al., 2015). Therefore, long-term direct investigation under the given environmental conditions is necessary for a precise understanding of the community dynamics.

We found substantial species-specific differences in the pattern of soil seed persistence both over time and depending on the burial depth. These differences were explained mainly on the species level for the proportion of viable seeds and the family level for the proportion of dormant seeds, which is in agreement with our knowledge of seed dormancy as the earliest trait in plant life history (Carta et al., 2016; Liu et al., 2017). Previous investigation in calcareous grassland communities showed essentially similar patterns in species-specific differences for constant burial depth (Pons, 1991). On the other hand, our results emphasized the importance of burial depth for particular species.

In our experiment, we cannot separate if seeds detected as non-viable after the given time of burial germinated in the soil before excavation or were destroyed due to pathogens attack. Mortality *via* fungi attack is both site-specific (Schafer and Kotanen, 2003) and species-specific (Gardarin et al., 2010). Nevertheless, the reason why seeds did not stay viable in the soil has not high importance for answering our questions about soil seed persistence. In both cases, such seeds do not play a role as seed supply in the soil and do not affect the long-term community dynamics.

As we expected, seeds of species with light requirements for germination stay viable longer in the deeper layer. Although light can penetrate only a tiny upper layer of soil (Kasperbauer and Hunt, 1988; Mandoli et al., 1990), seedlings, especially of large-seeded species, can emerge successfully from much greater depth (Bond et al., 1999). Generally, germination in light conditions is one of the mechanisms to detect the burial depth. It was shown before that light requirement is essential to keep seeds ungerminated just after entering the soil (Saatkamp et al., 2011b). Our findings of the longer persistence of seeds in deeper soil layers for species with light requirements for germination support this idea. Huge differences between individual species were found, and we can agree with Saatkamp et al. (2011a) that burial depth detection is a highly species-specific mechanism. Different species with light requirements for germination showed different patterns in soil seed persistence. For example, seeds of *B. falcatum* and *Linum catharticum* did not stay viable in the upper soil layer, which suggests that they germinate immediately after burial in light conditions. Seeds of these species stay viable during one season in deeper soil layers, and after this time, the number of viable seeds decreased to the same number as in the upper soil layer. Conditions in deeper soil layers postponed the decline of a count of viable seeds, but they did not guarantee their long-term survival.

Furthermore, we found the group of species with light requirements for germination, namely, *C. acaule, D. carota, H. perforatum, O. vulgare*, and *Leontodon hispidus*, which showed another pattern in soil seed persistence. Seeds of these species survived in a similar amount after the first winter in all burial depths. Later, the number of viable seeds decreased substantially in the upper soil layer. In contrast, at deeper burial depth, their number remained constant throughout the experiment. On the other hand, high seed persistence in the deeper soil layer throughout the experiment and at the same time, constantly low number of viable seeds in the upper soil layer was found for species *G. germanica* and *Lactuca serriola*. That indicates that detecting the burial depth is crucial for these species immediately after burial; they are not able to germinate from deeper soil layers. They can stay viable in the soil seed bank for a long time, although both are often classified as transient soil seed banks (i.e., their seeds should persist in the soil less than one year) in the literature (Pons, 1991; Kleyer et al., 2008). We found around 50% of seeds viable after two winters in the deep soil layer and around 20% of seeds viable in the upper soil layer for *G. germanica*. This inconsistency between classification as transient soil seed bank from literature and our finding of viable seeds after two years of burial could result from the long dormancy, so classical germination experiments cannot detect it, as reported by Fischer and Matthies (1998).

Our results of differences between species with and without alternating temperature requirements for germination match our expectations. The proportion of viable seeds changes with the burial depth and during the time for species with such requirements. This relationship was weak, and we see the main reason for the unbalanced design of our dataset. We have only three species out of 28 with alternating temperature requirements for germination, which differ in other seed traits, so it is challenging to generalize them. Alternating temperature requirements are known as the mechanism which can serve for detection of burial depth but simultaneously also for detection of disturbances (Thompson and Grime, 1983; Saatkamp et al., 2011a). Nevertheless, its role in soil seed persistence in some environments seems to be negligible (Rivera et al., 2012). Therefore, further investigation with the precise selection of species according to this seed trait and careful setup of the experiment is needed to disentangle the role of alternating temperature for soil seed persistence.

Our work showed a broad range of soil seed persistence strategies under the different burial depths. This diversity can potentially promote species coexistence by the storage effect (Chesson, 1994; Facelli et al., 2005) and thereby maintaining a species-rich community that can withstand temporal fluctuations in environmental conditions. On the other hand, it is known that current climatic changes, such as changing temperature and rainfall regimes, can accelerate the decline of seed viability (Chen et al., 2021), compromising the persistence of plant populations dependent on long-lived seed banks (Ooi, 2012) or dry habitats (Basto et al., 2018). Our results from the long-term burial experiment also proved that seeds of some species could survive in the soil much longer than expected from the indirect measurement of seed longevity by the seedling establishment from soil samples. It points out the importance of further direct long-term investigation.

## Data Availability Statement

The raw data supporting the conclusions of this article will be made available by the authors, without undue reservation.

## Author Contributions

PP designed the study and performed the experiments. TM analyzed the data. TM and PP interpreted results. TM wrote the text with contributions of PP. All authors approved the final version of the manuscript.

## Conflict of Interest

The authors declare that the research was conducted in the absence of any commercial or financial relationships that could be construed as a potential conflict of interest. The handling Editor declared a past co-authorship with one of the authors PP.

## Publisher’s Note

All claims expressed in this article are solely those of the authors and do not necessarily represent those of their affiliated organizations, or those of the publisher, the editors and the reviewers. Any product that may be evaluated in this article, or claim that may be made by its manufacturer, is not guaranteed or endorsed by the publisher.

## Footnotes

1
http://enscobase.maich.gr/

2
http://data.kew.org/sid

## Supplementary Material

Supplementary Figure 1 The species-specific pattern in the proportion of viable seeds during the time and different burial depths.

Supplementary Figure 2 The species-specific pattern in the proportion of dormant seeds during the time and different burial depths.
