# Supplementary material for: Soil Seed Bank Persistence Across Time and Burial Depth in Calcareous Grassland Habitats
Source: Front Plant Sci. 2022 Feb 4;12:790867. doi: 10.3389/fpls.2021.790867 (PMC8854790; doi:10.3389/fpls.2021.790867)
Supplement: Supplementary Figure 1 — The species-specific pattern in the proportion of viable seeds during the time and different burial depths. [file Data_Sheet_1.PDF]

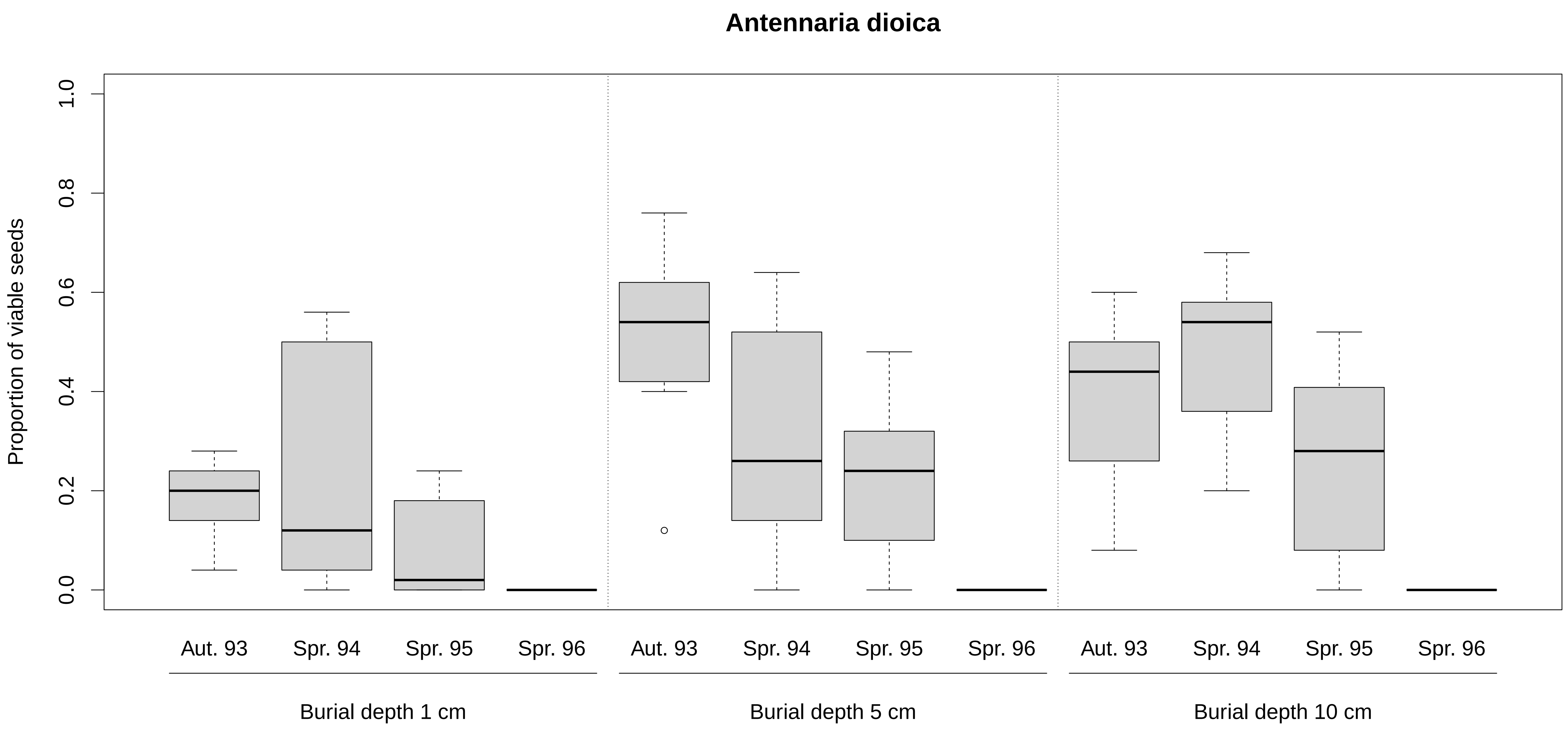
Antennaria dioica
Proportion of viable seeds
0.0
0.2
0.4
0.6
0.8
1.0
Aut. 93
Spr. 94
Spr. 95
Spr. 96
Aut. 93
Spr. 94
Spr. 95
Spr. 96
Aut. 93
Spr. 94
Spr. 95
Spr. 96
Burial depth 1 cm
Burial depth 5 cm
Burial depth 10 cm

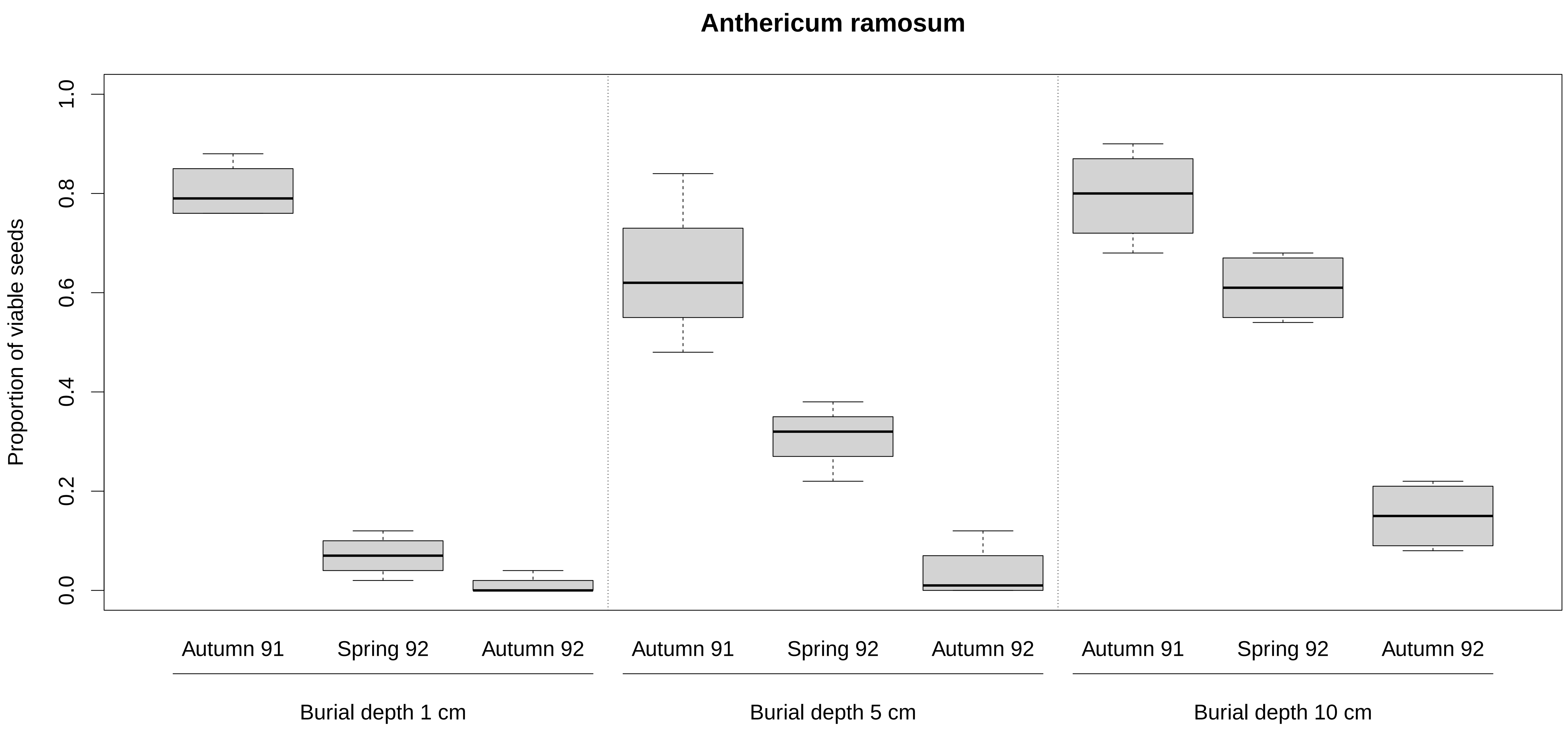
Anthericum ramosum
Proportion of viable seeds
0.0
0.2
0.4
0.6
0.8
1.0
Autumn 91
Spring 92
Autumn 92
Autumn 91
Spring 92
Autumn 92
Autumn 91
Spring 92
Autumn 92
Burial depth 1 cm
Burial depth 5 cm
Burial depth 10 cm

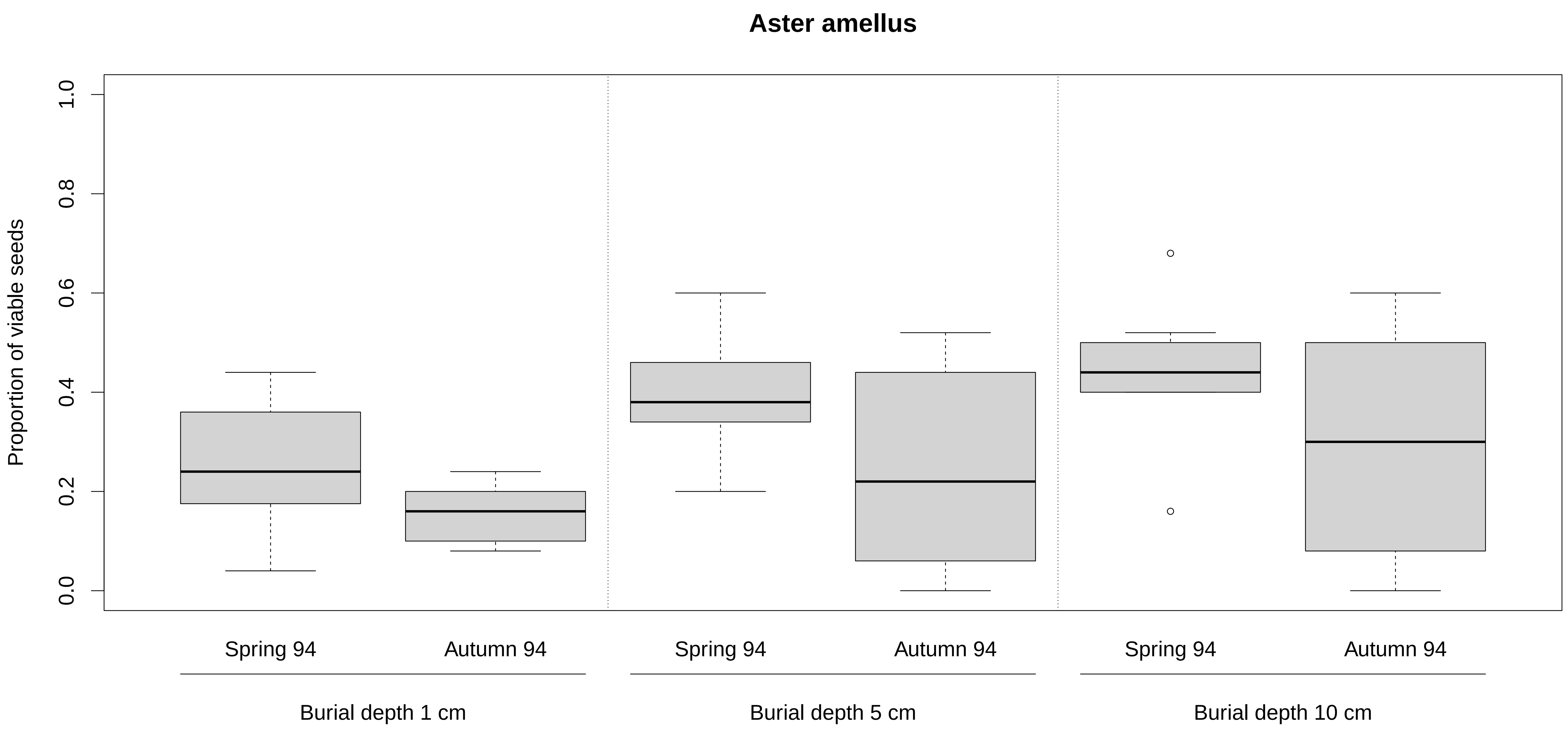
Aster amellus
Proportion of viable seeds
0.0
0.2
0.4
0.6
0.8
1.0
Spring 94
Autumn 94
Spring 94
Autumn 94
Spring 94
Autumn 94
Burial depth 1 cm
Burial depth 5 cm
Burial depth 10 cm

Brachypodium pinnatum

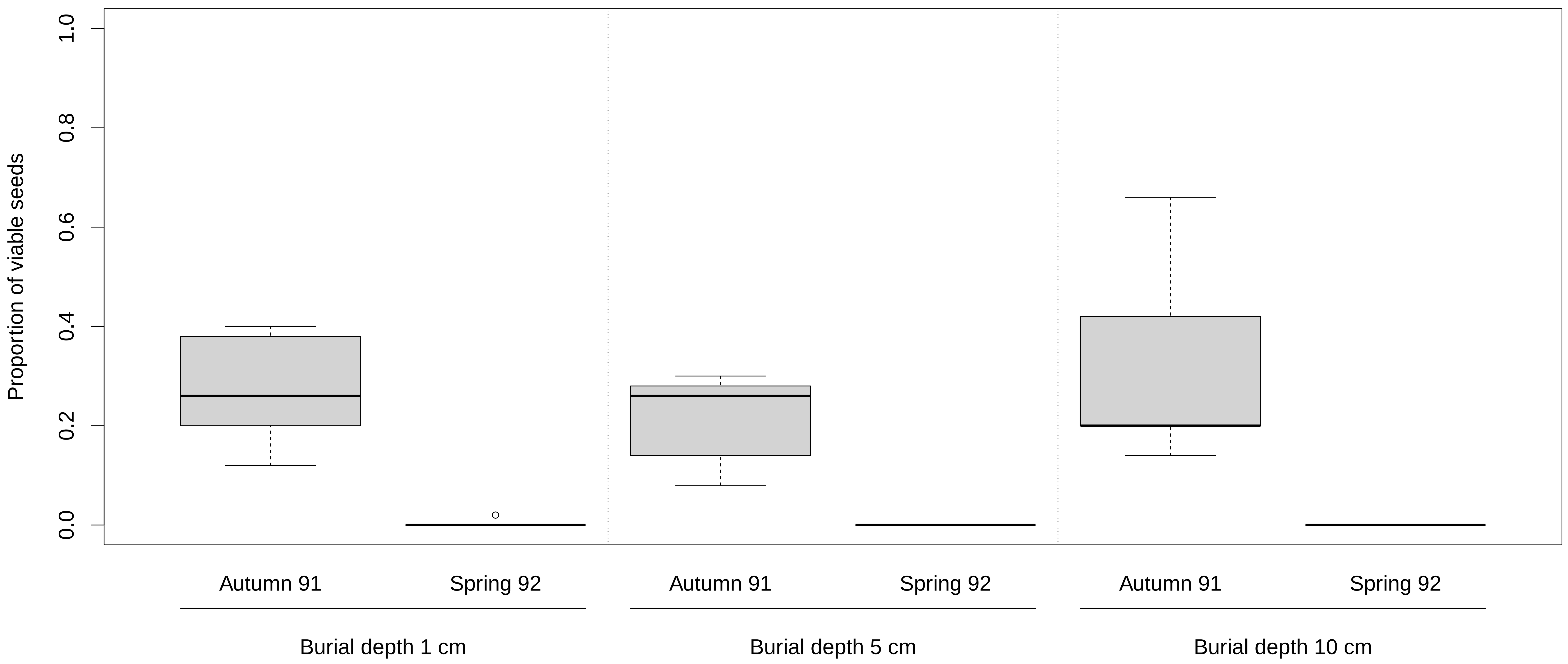

Bromus erectus

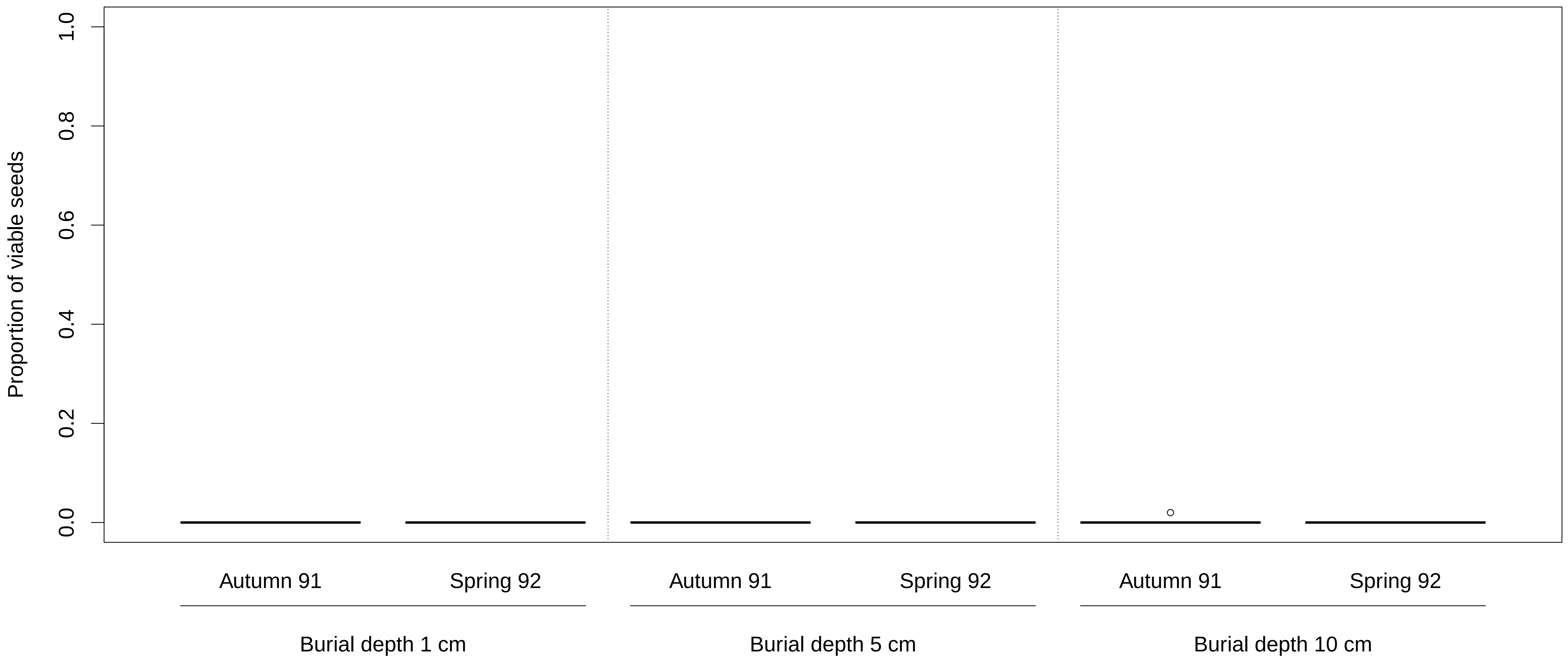

Bupleurum falcatum

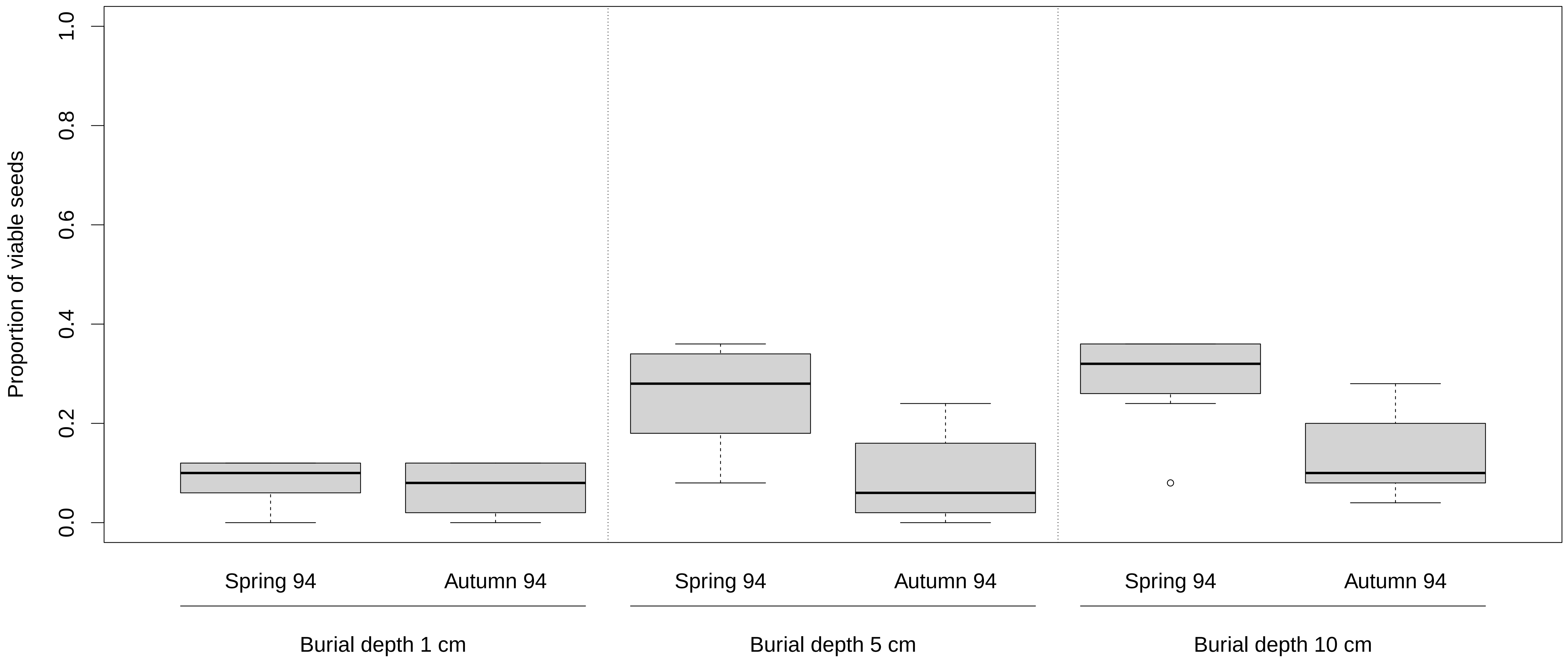

Carex flacca

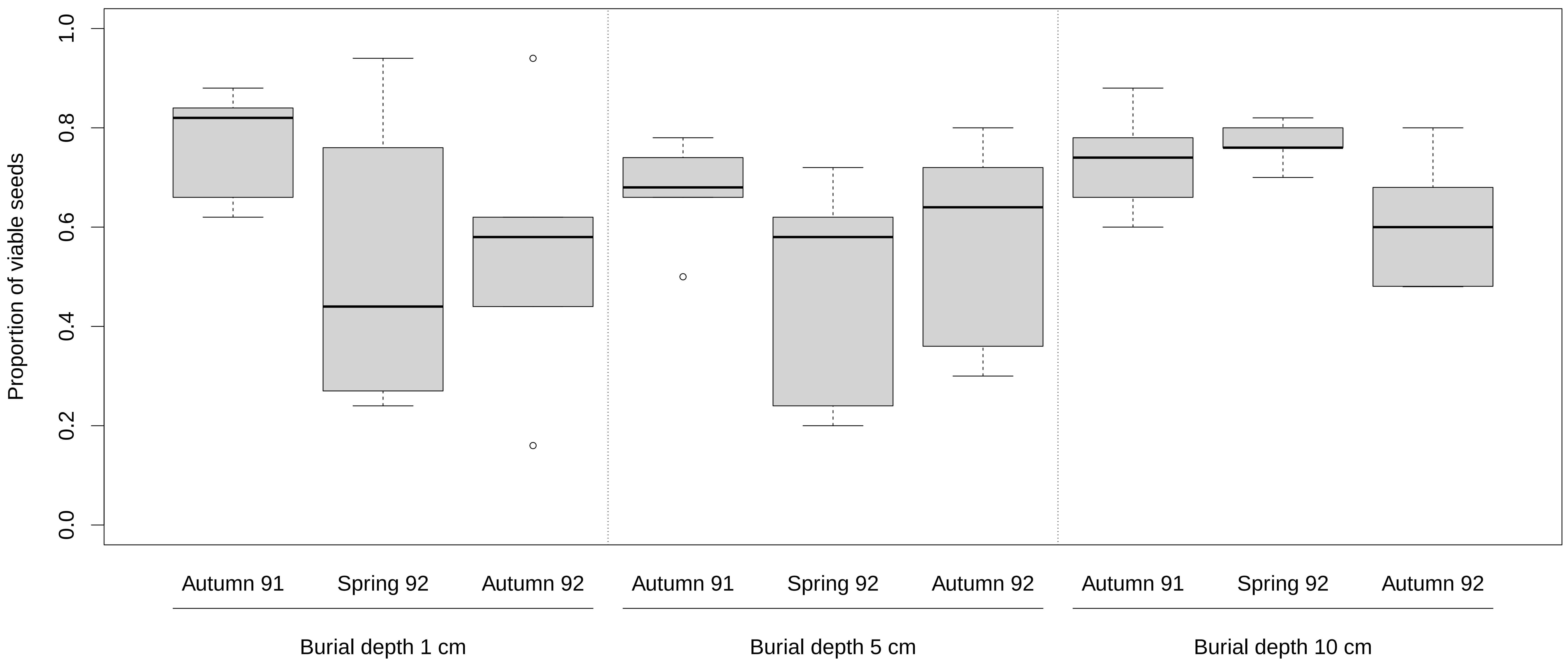

Carlina acaulis

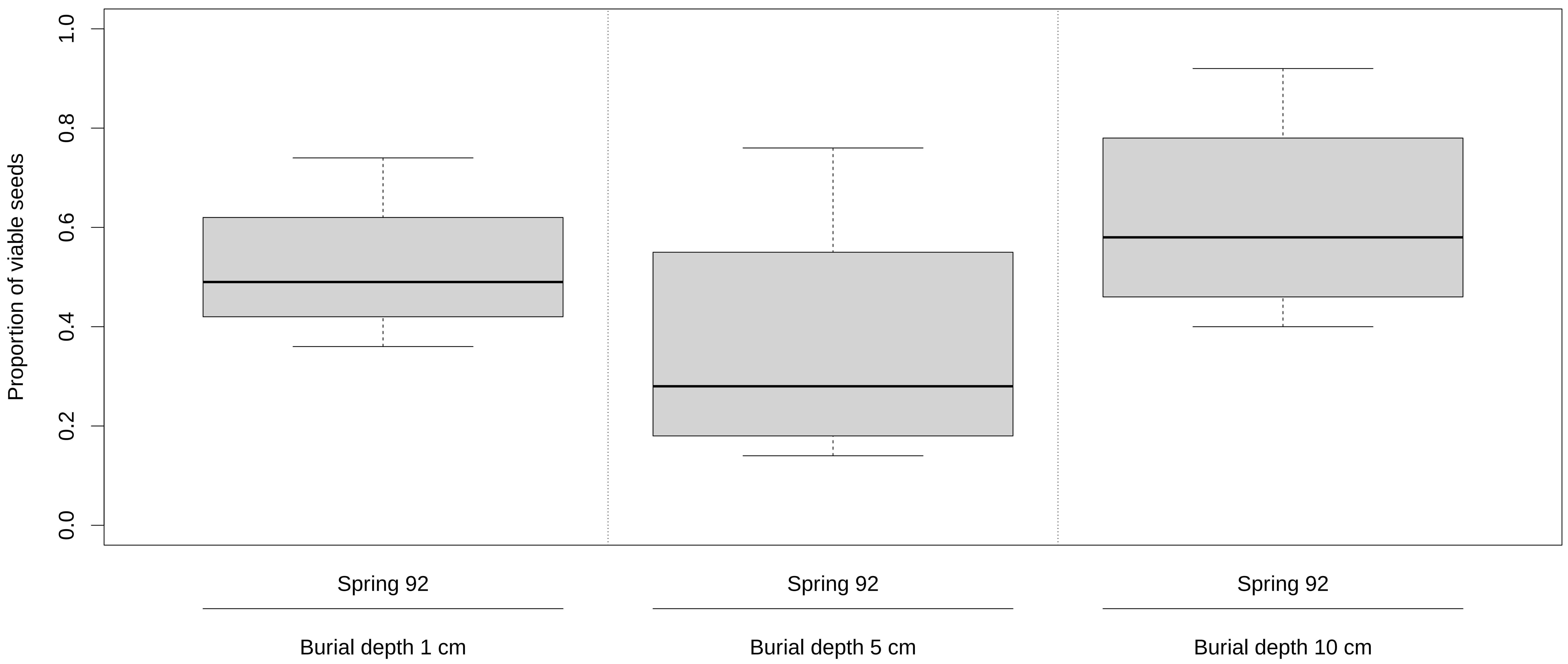

Carlina vulgaris

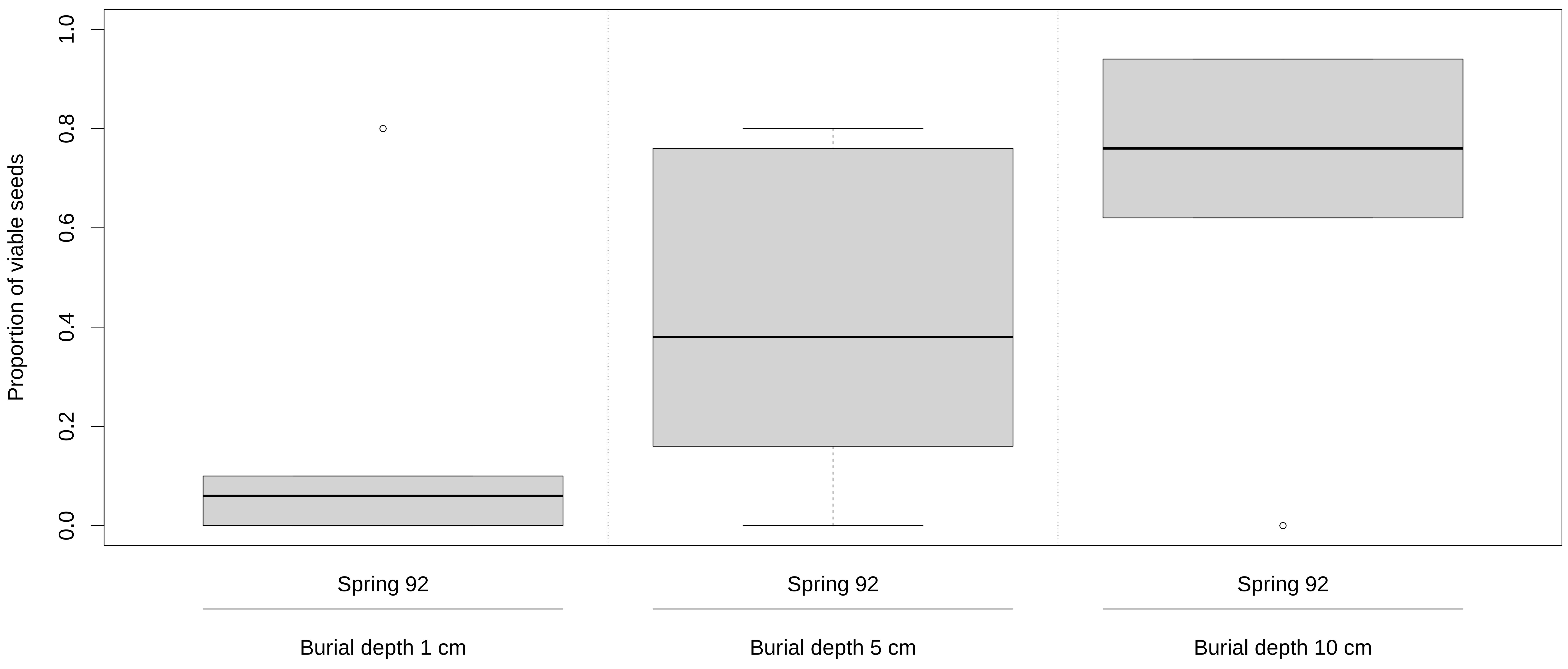

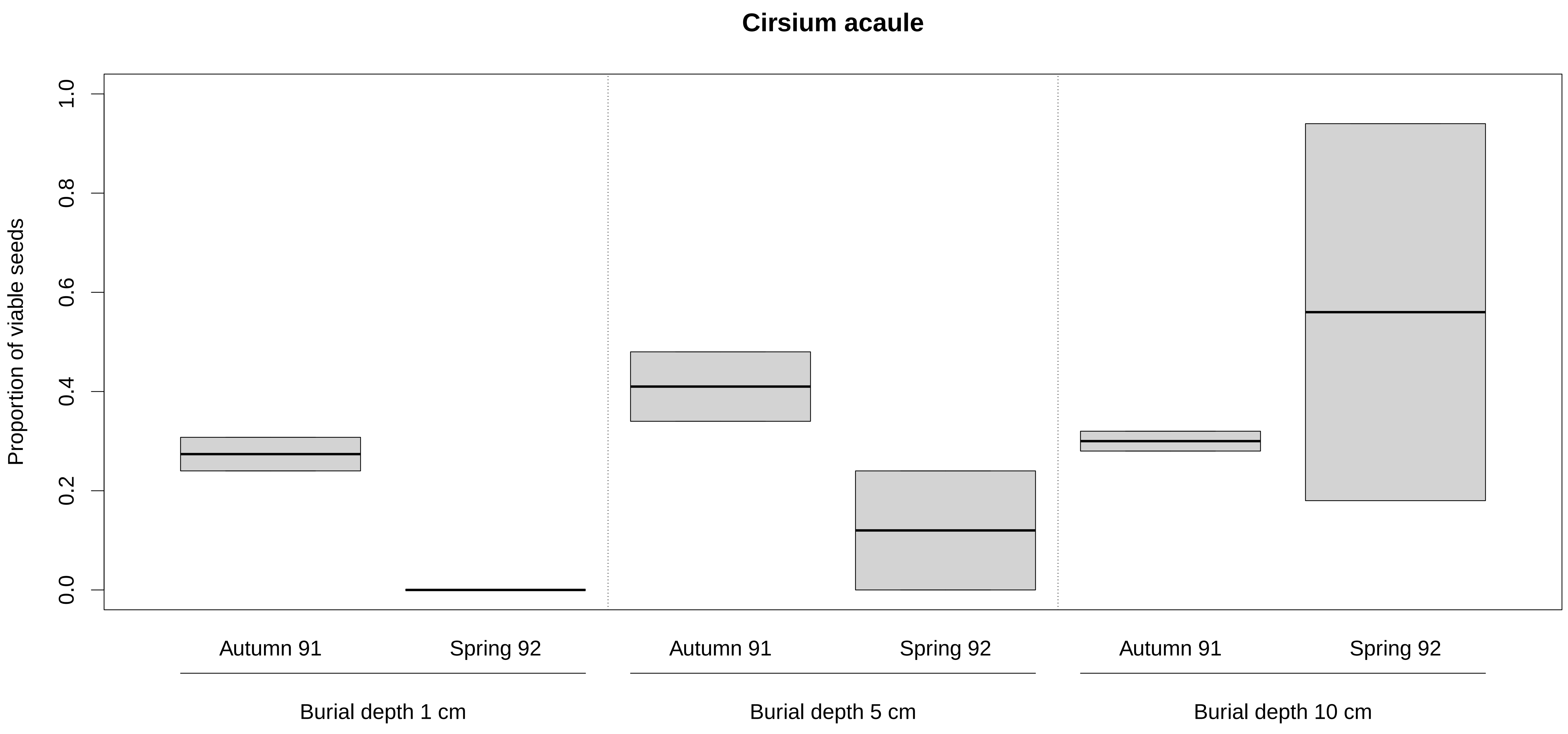
Cirsium acaule
Proportion of viable seeds
0.0
0.2
0.4
0.6
0.8
1.0
Autumn 91
Spring 92
Autumn 91
Spring 92
Autumn 91
Spring 92
Burial depth 1 cm
Burial depth 5 cm
Burial depth 10 cm

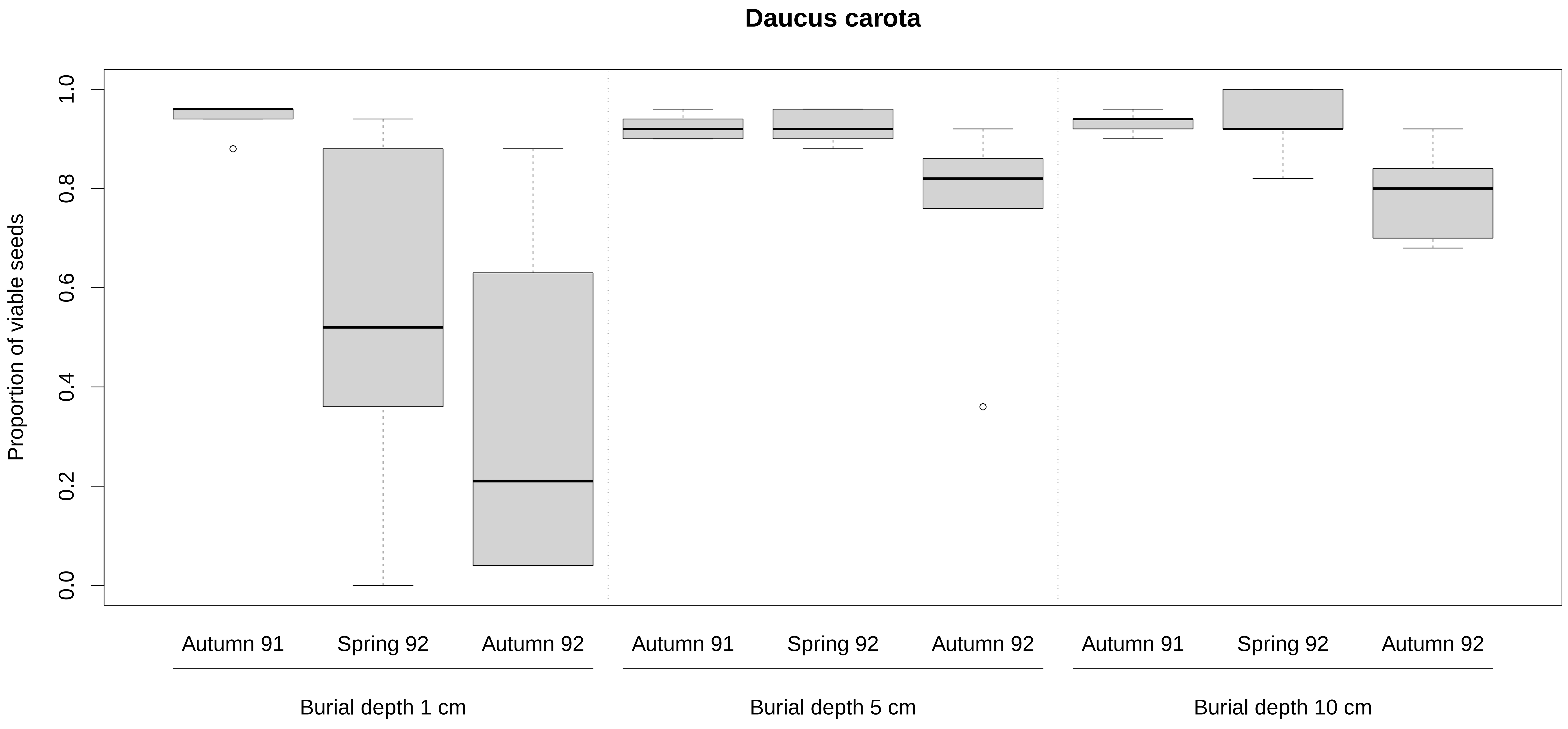
Daucus carota
Proportion of viable seeds
0.0
0.2
0.4
0.6
0.8
1.0
Autumn 91
Spring 92
Autumn 92
Autumn 91
Spring 92
Autumn 92
Autumn 91
Spring 92
Autumn 92
Burial depth 1 cm
Burial depth 5 cm
Burial depth 10 cm

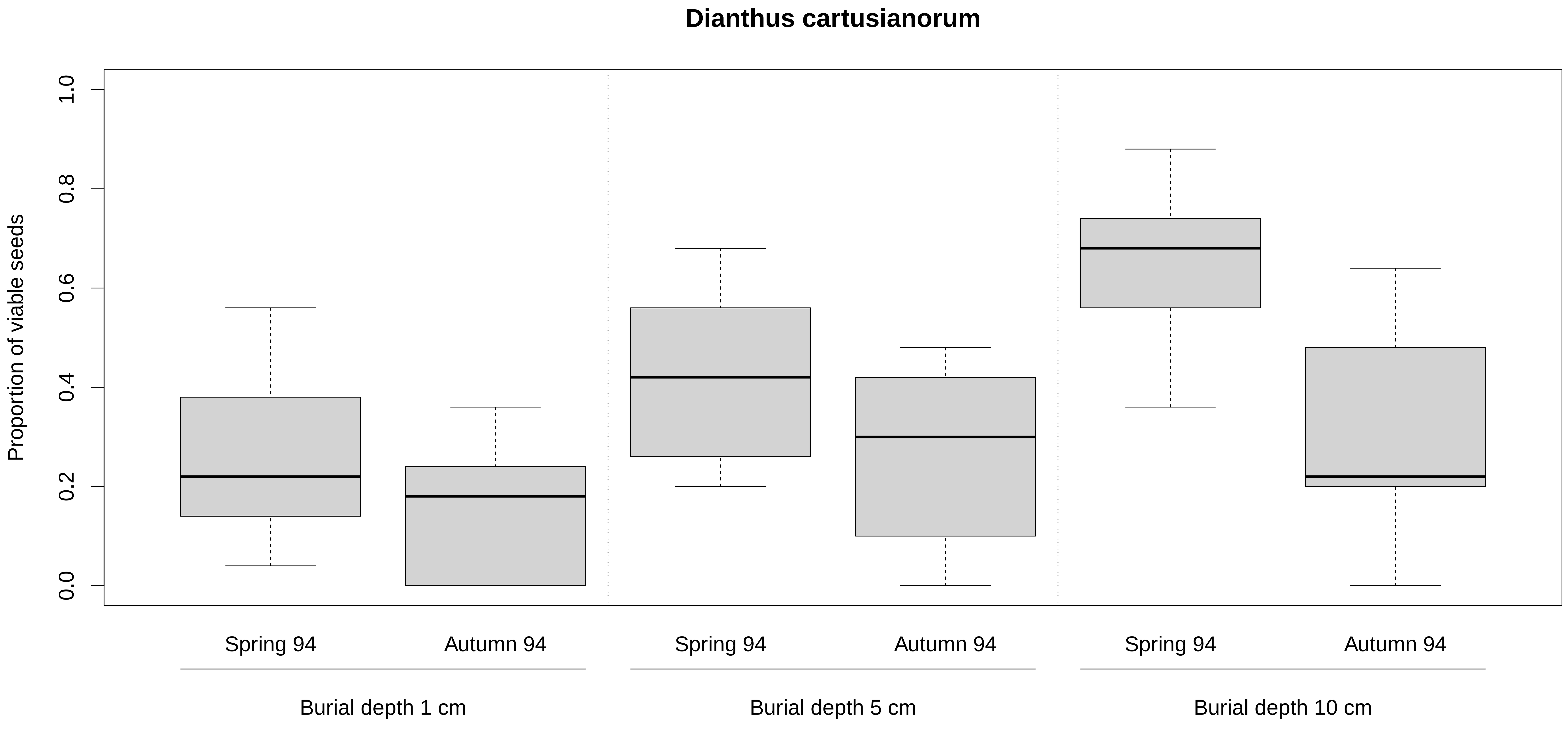
Dianthus cartusianorum
Proportion of viable seeds
0.0
0.2
0.4
0.6
0.8
1.0
Spring 94
Autumn 94
Spring 94
Autumn 94
Spring 94
Autumn 94
Burial depth 1 cm
Burial depth 5 cm
Burial depth 10 cm

Festuca ovina

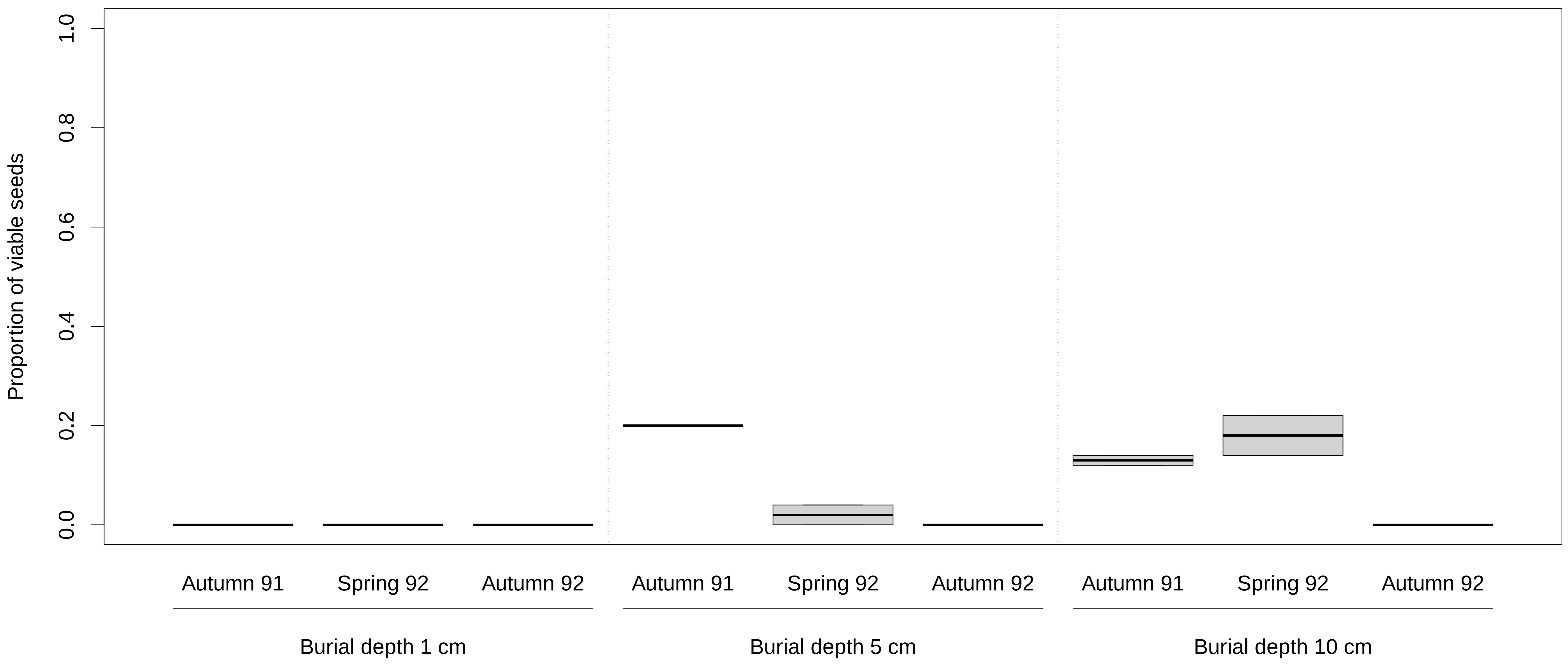

Gentianella germanica

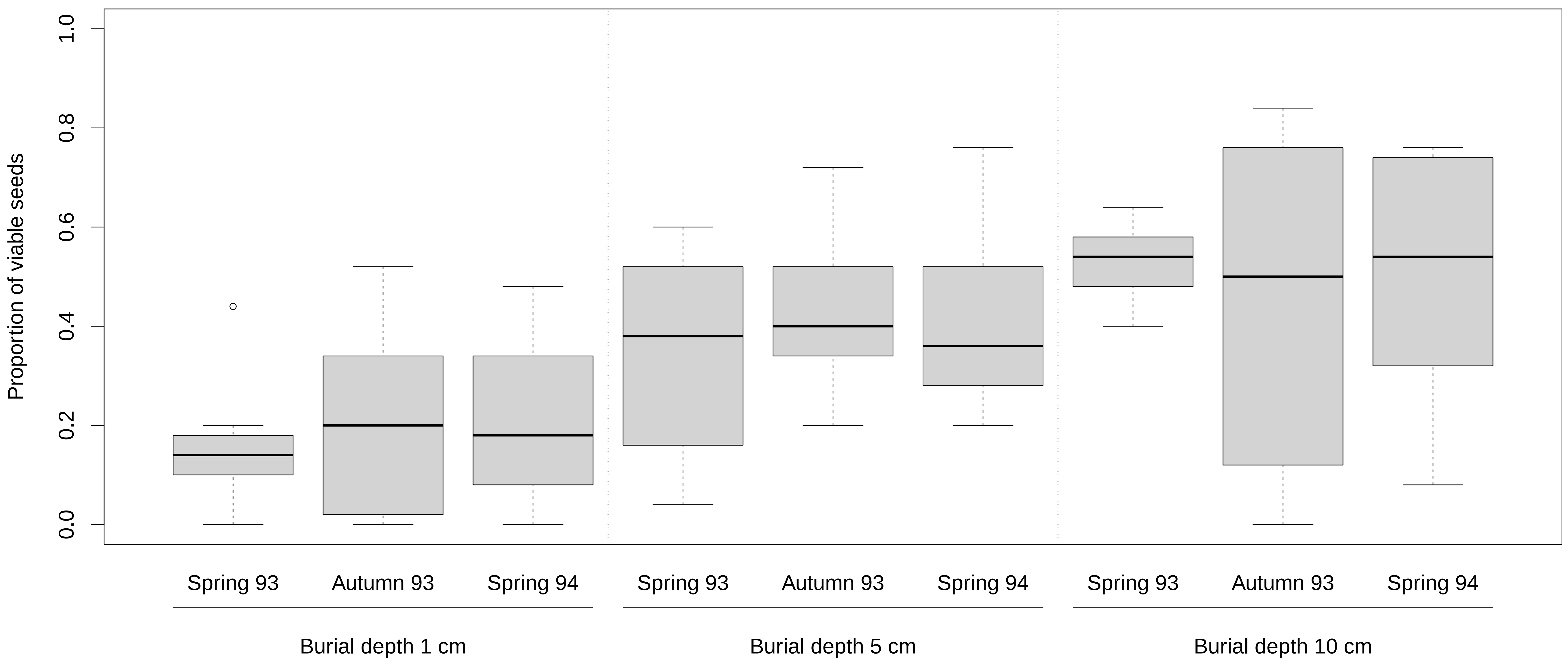

Globularia elongata

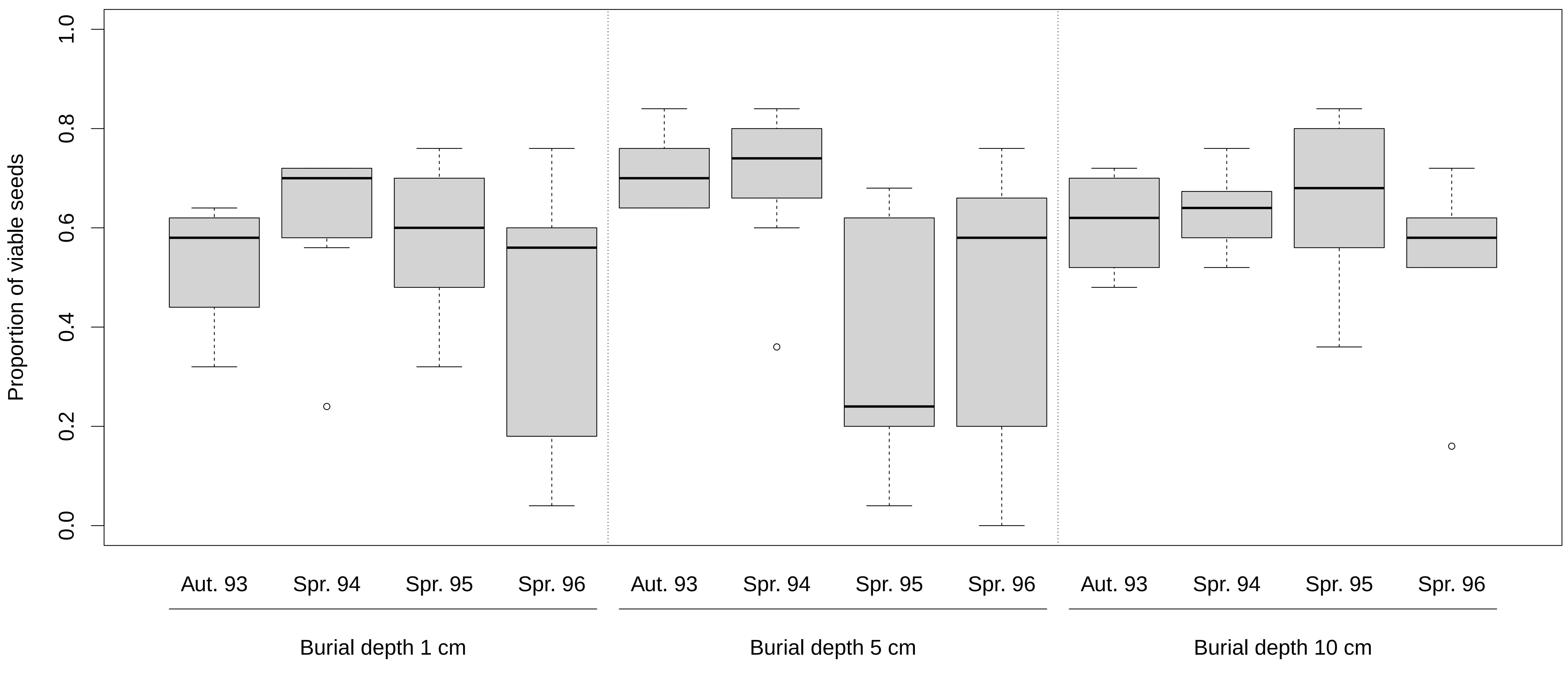

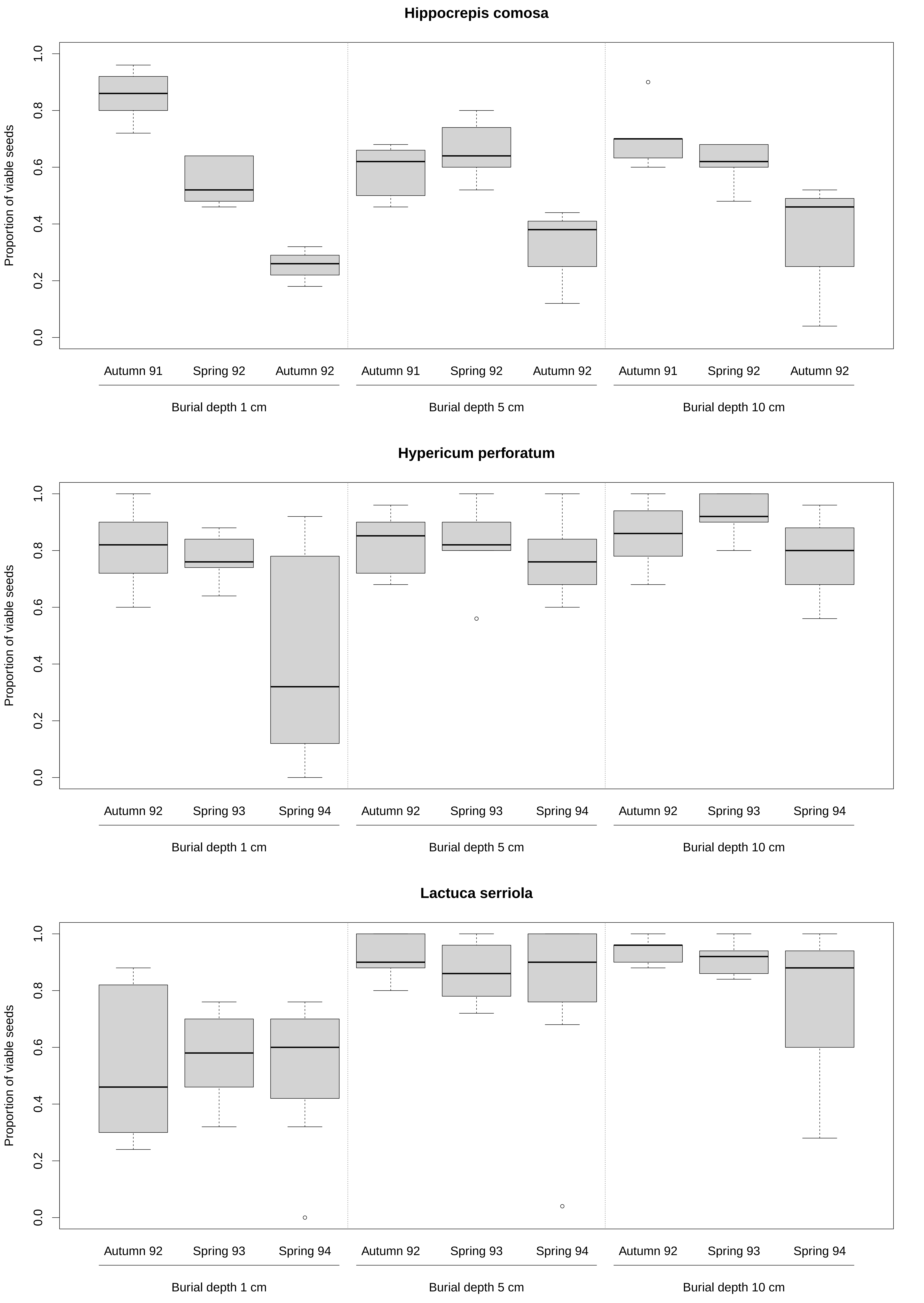
Hippocrepis comosa
Proportion of viable seeds
0.0
0.2
0.4
0.6
0.8
1.0
Autumn 91
Spring 92
Autumn 92
Burial depth 1 cm
Burial depth 5 cm
Burial depth 10 cm
Hypericum perforatum
Autumn 92
Spring 93
Spring 94
Lactuca serriola

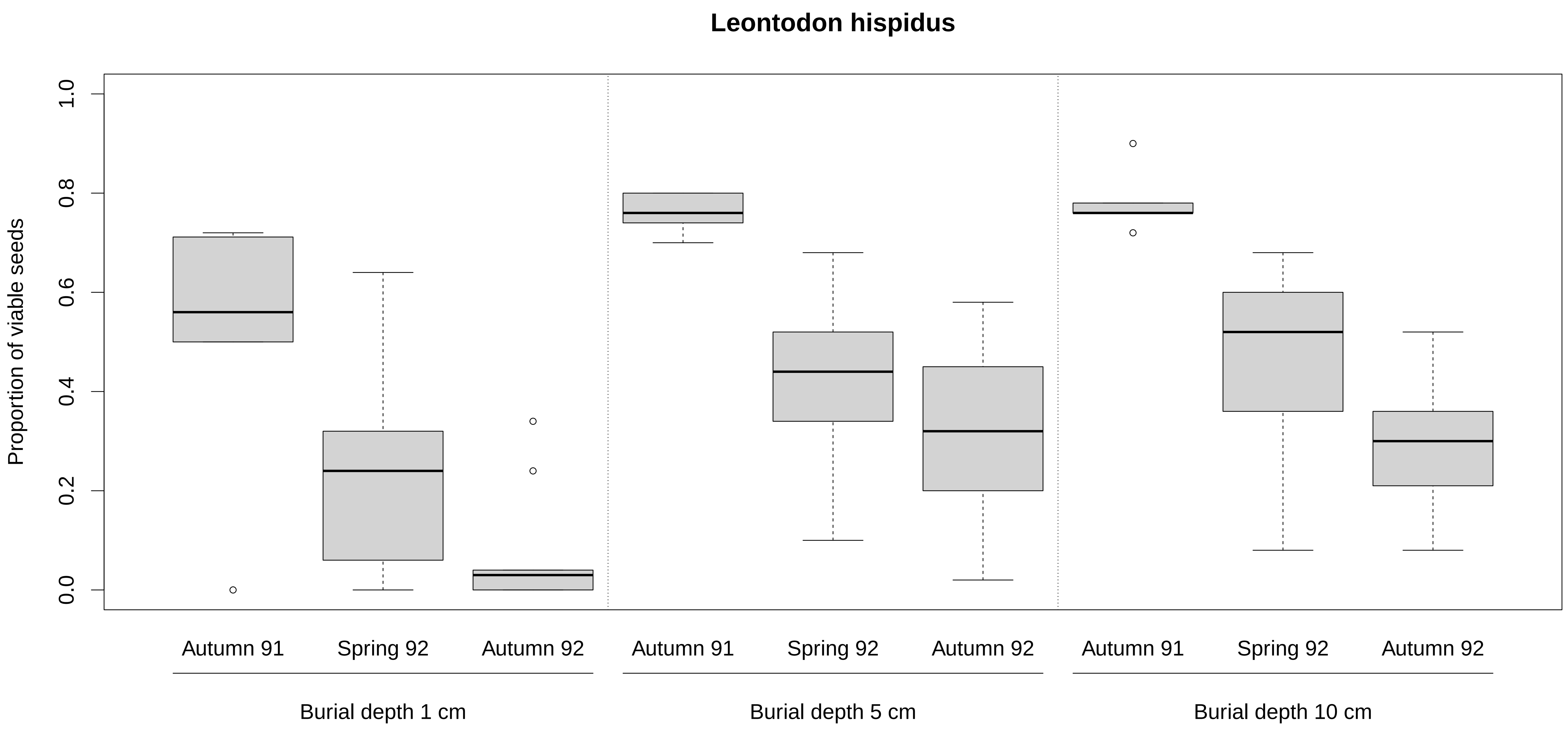
Leontodon hispidus
Proportion of viable seeds
0.0
0.2
0.4
0.6
0.8
1.0
Autumn 91
Spring 92
Autumn 92
Autumn 91
Spring 92
Autumn 92
Autumn 91
Spring 92
Autumn 92
Burial depth 1 cm
Burial depth 5 cm
Burial depth 10 cm

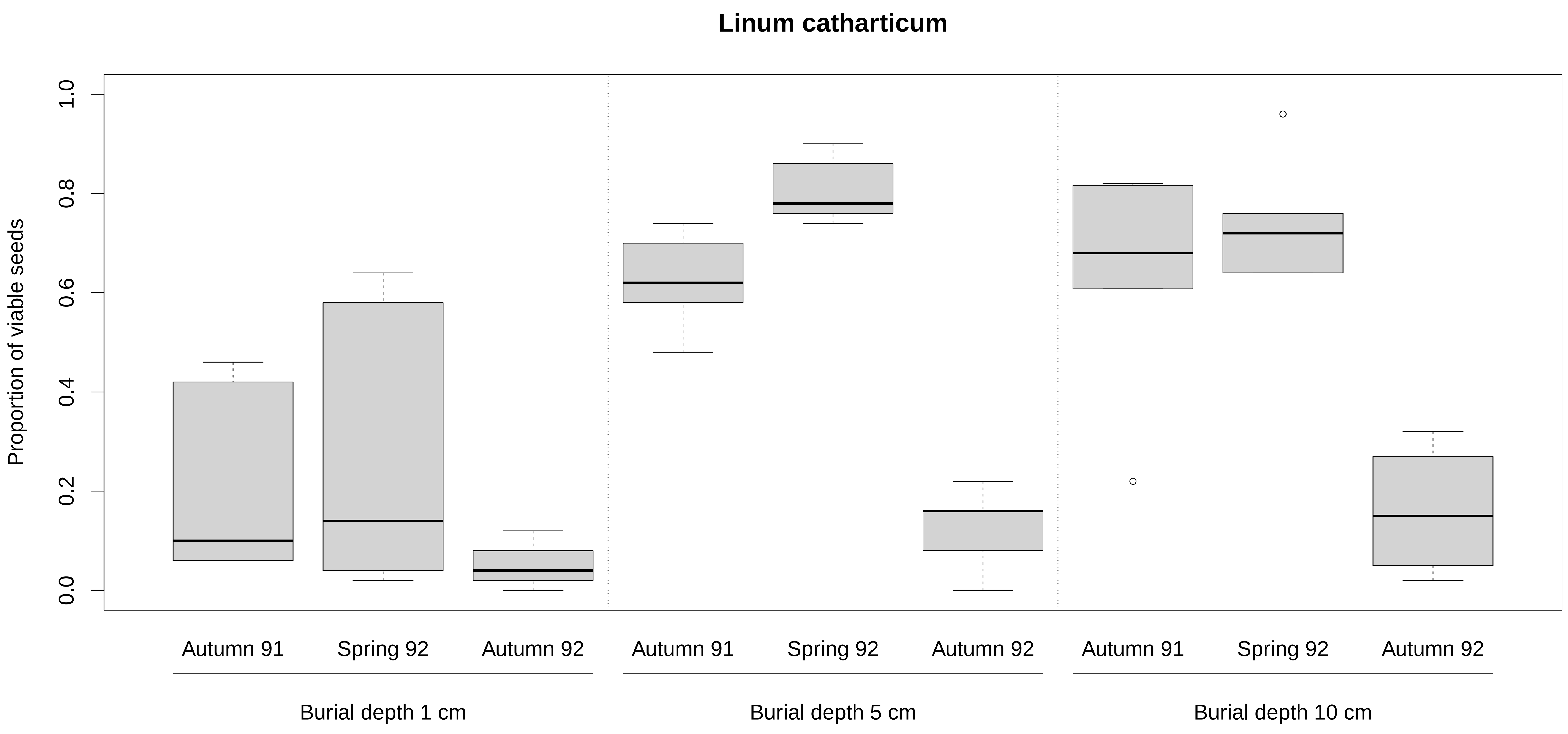
Linum catharticum
Proportion of viable seeds
0.0
0.2
0.4
0.6
0.8
1.0
Autumn 91
Spring 92
Autumn 92
Autumn 91
Spring 92
Autumn 92
Autumn 91
Spring 92
Autumn 92
Burial depth 1 cm
Burial depth 5 cm
Burial depth 10 cm

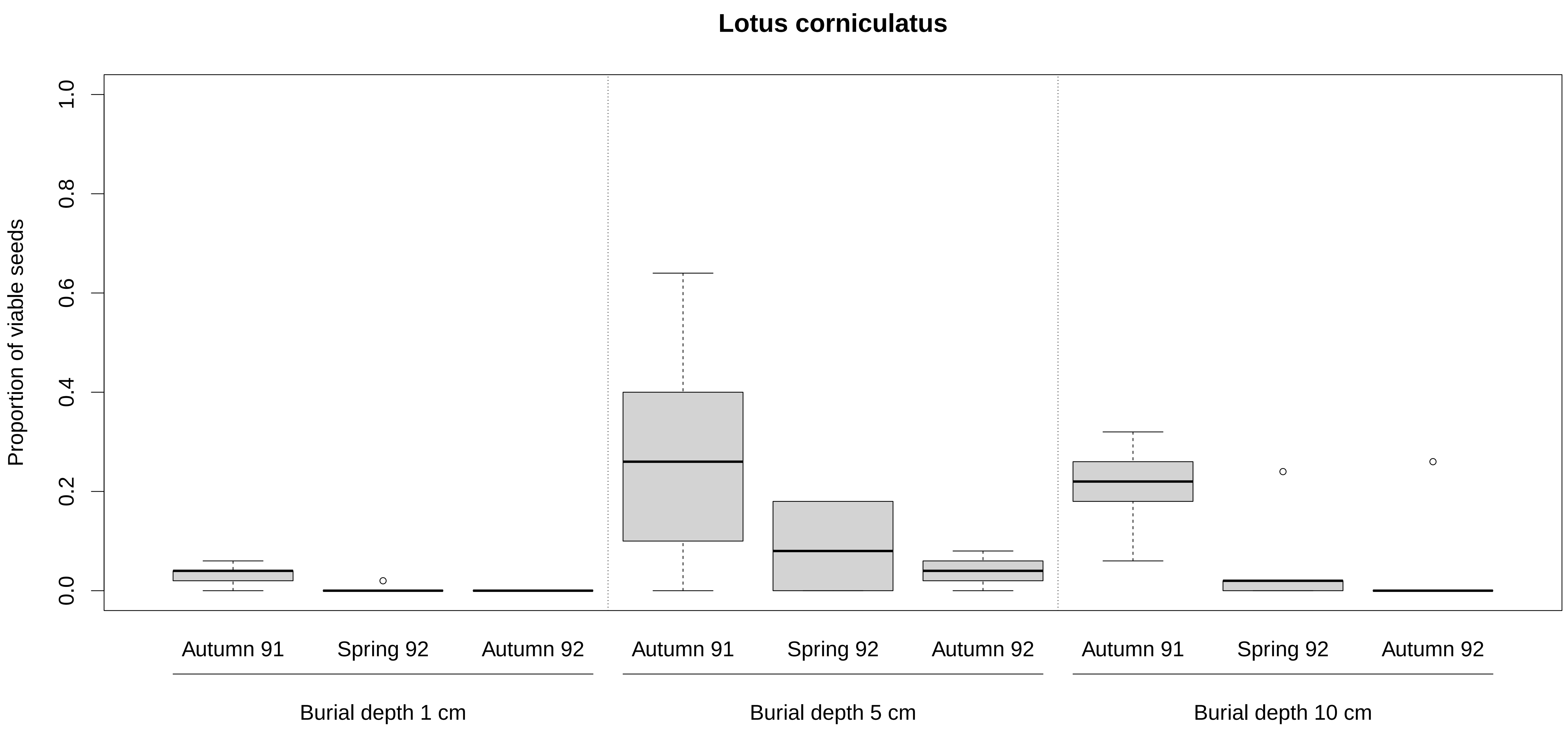
Lotus corniculatus
Proportion of viable seeds
0.0
0.2
0.4
0.6
0.8
1.0
Autumn 91
Spring 92
Autumn 92
Autumn 91
Spring 92
Autumn 92
Autumn 91
Spring 92
Autumn 92
Burial depth 1 cm
Burial depth 5 cm
Burial depth 10 cm

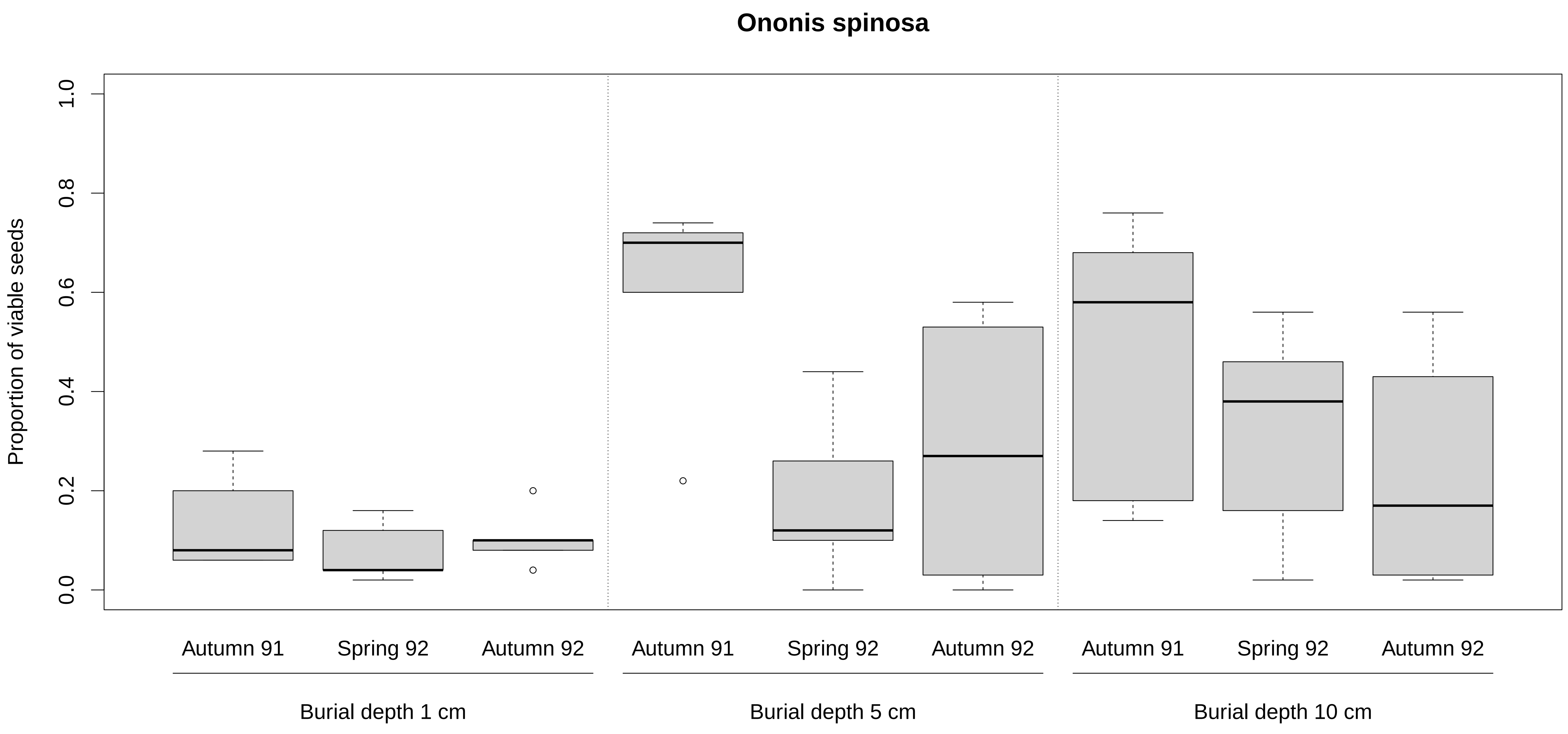
Ononis spinosa
Proportion of viable seeds
0.0
0.2
0.4
0.6
0.8
1.0
Autumn 91
Spring 92
Autumn 92
Autumn 91
Spring 92
Autumn 92
Autumn 91
Spring 92
Autumn 92
Burial depth 1 cm
Burial depth 5 cm
Burial depth 10 cm

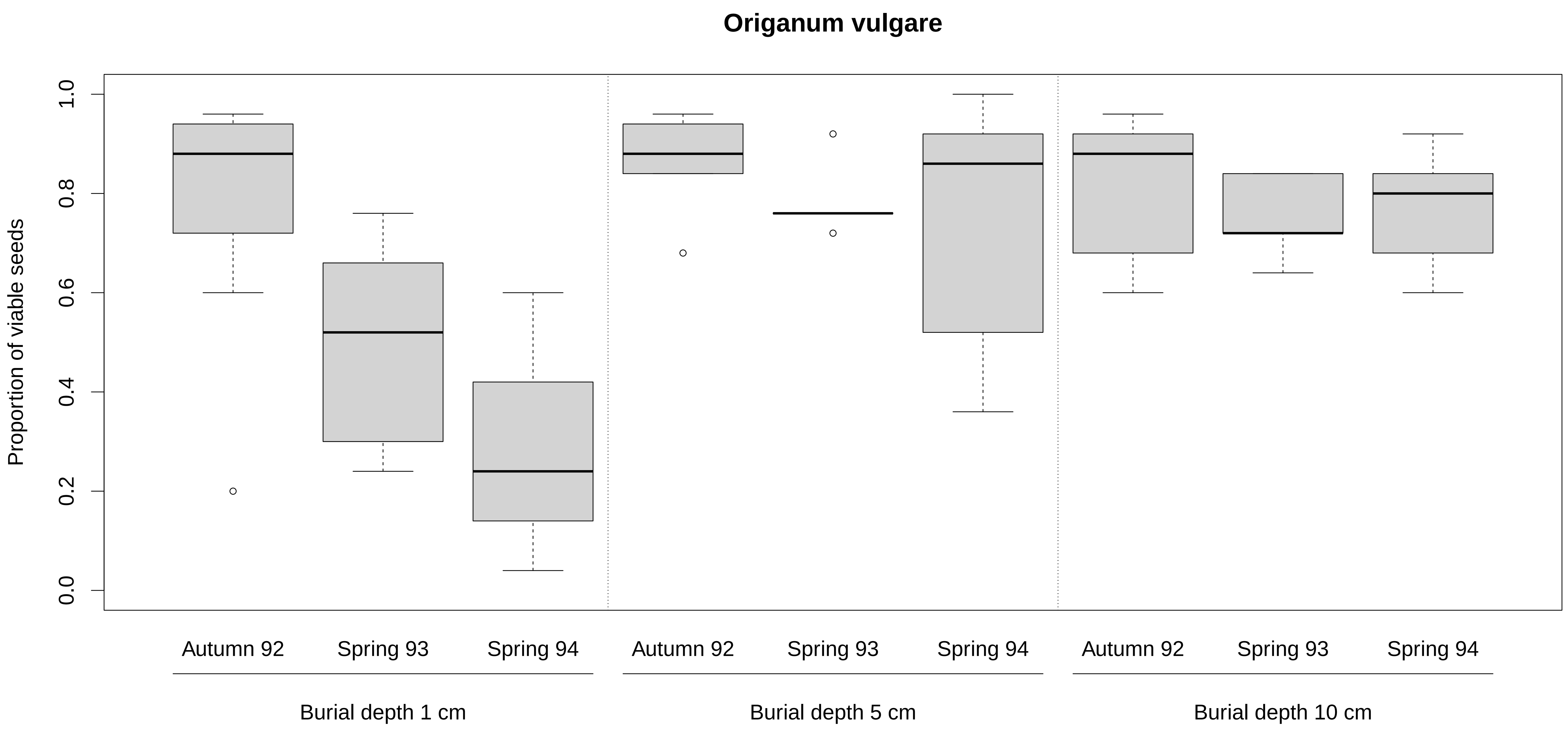
Origanum vulgare
Proportion of viable seeds
0.0
0.2
0.4
0.6
0.8
1.0
Autumn 92
Spring 93
Spring 94
Autumn 92
Spring 93
Spring 94
Autumn 92
Spring 93
Spring 94
Burial depth 1 cm
Burial depth 5 cm
Burial depth 10 cm

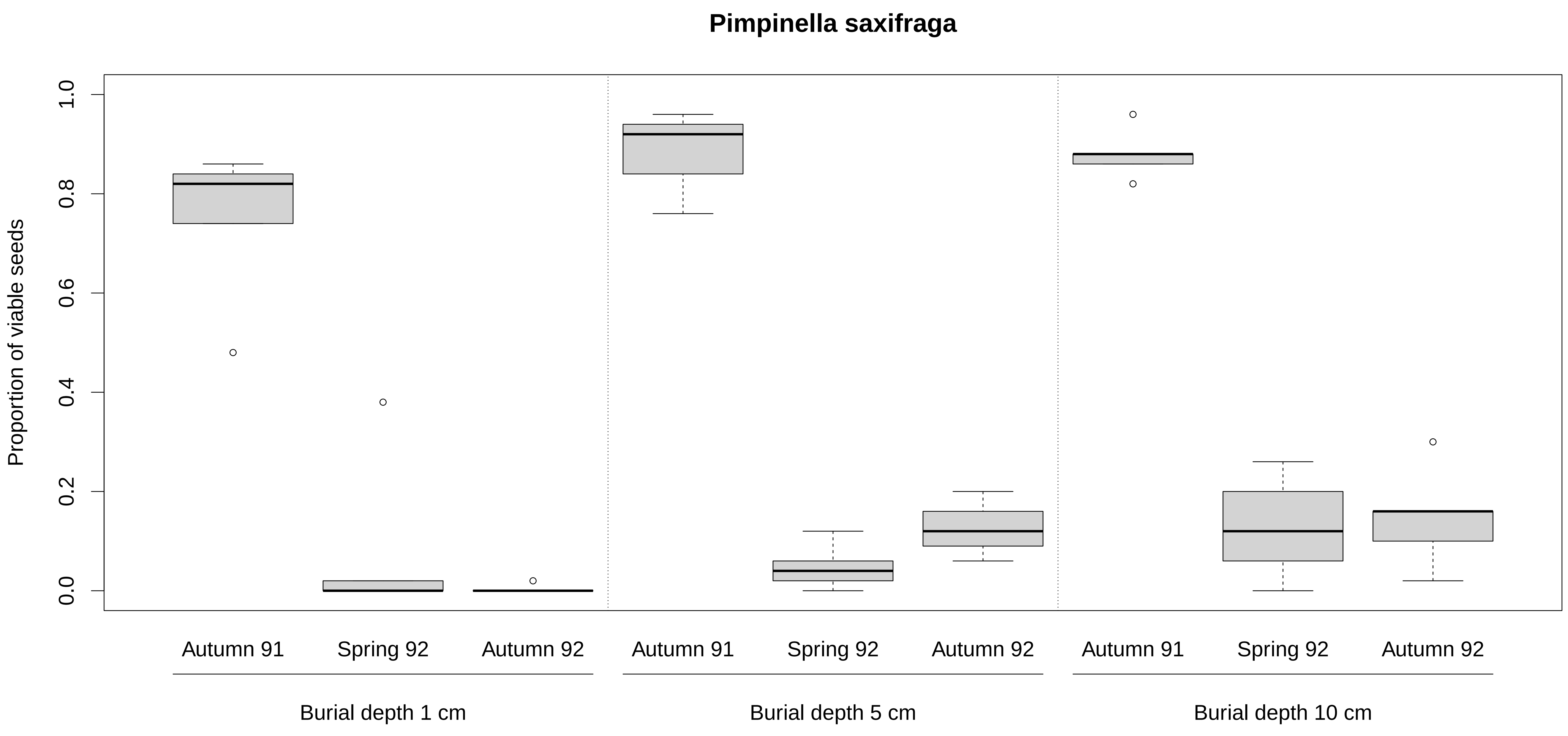
Pimpinella saxifraga
Proportion of viable seeds
0.0
0.2
0.4
0.6
0.8
1.0
Autumn 91
Spring 92
Autumn 92
Autumn 91
Spring 92
Autumn 92
Autumn 91
Spring 92
Autumn 92
Burial depth 1 cm
Burial depth 5 cm
Burial depth 10 cm

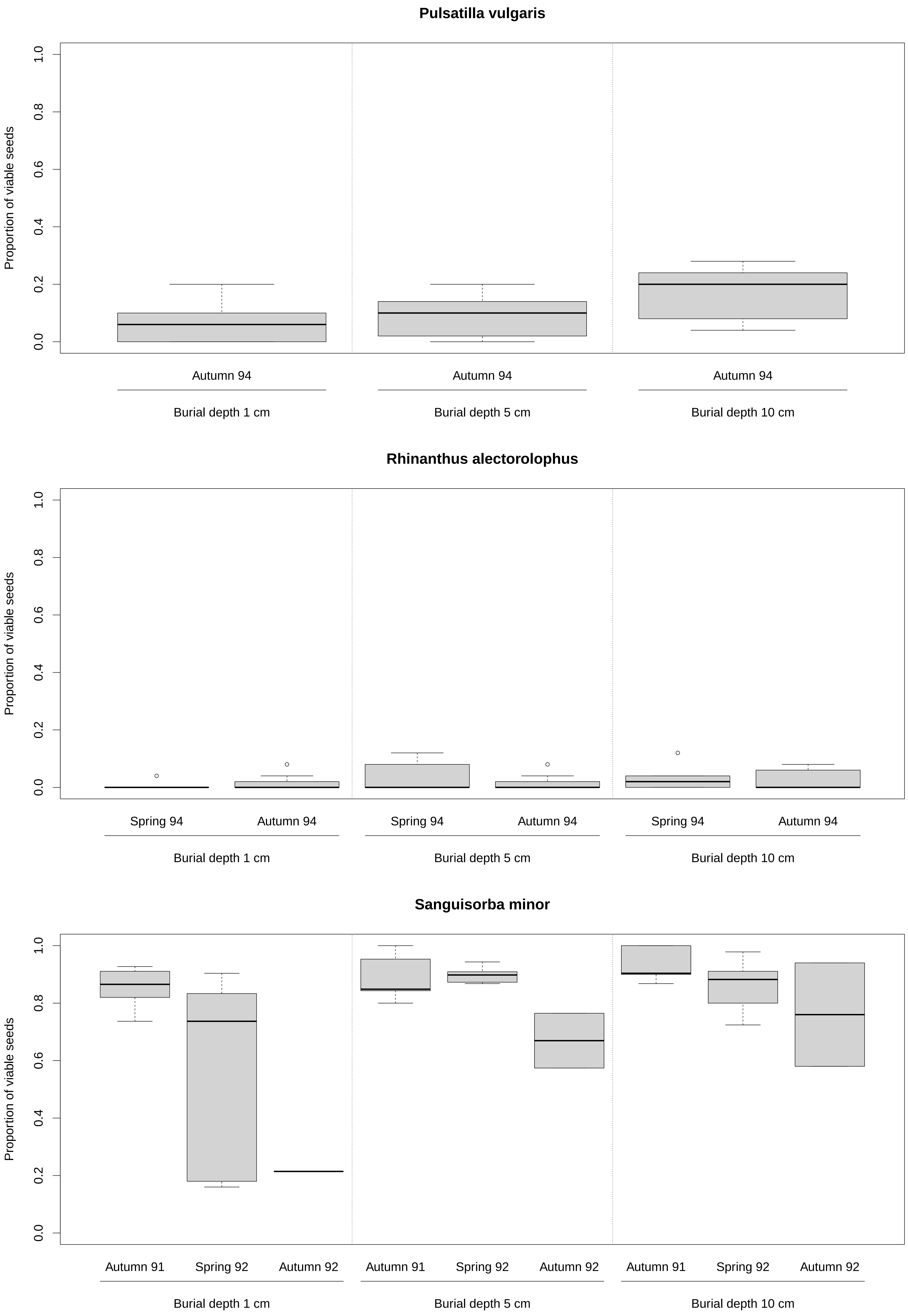
Pulsatilla vulgaris
Proportion of viable seeds
0.0
0.2
0.4
0.6
0.8
1.0
Autumn 94
Autumn 94
Autumn 94
Burial depth 1 cm
Burial depth 5 cm
Burial depth 10 cm
Rhinanthus alectorolophus
Proportion of viable seeds
0.0
0.2
0.4
0.6
0.8
1.0
Spring 94
Autumn 94
Spring 94
Autumn 94
Spring 94
Autumn 94
Burial depth 1 cm
Burial depth 5 cm
Burial depth 10 cm
Sanguisorba minor
Proportion of viable seeds
0.0
0.2
0.4
0.6
0.8
1.0
Autumn 91
Spring 92
Autumn 92
Autumn 91
Spring 92
Autumn 92
Autumn 91
Spring 92
Autumn 92
Burial depth 1 cm
Burial depth 5 cm
Burial depth 10 cm

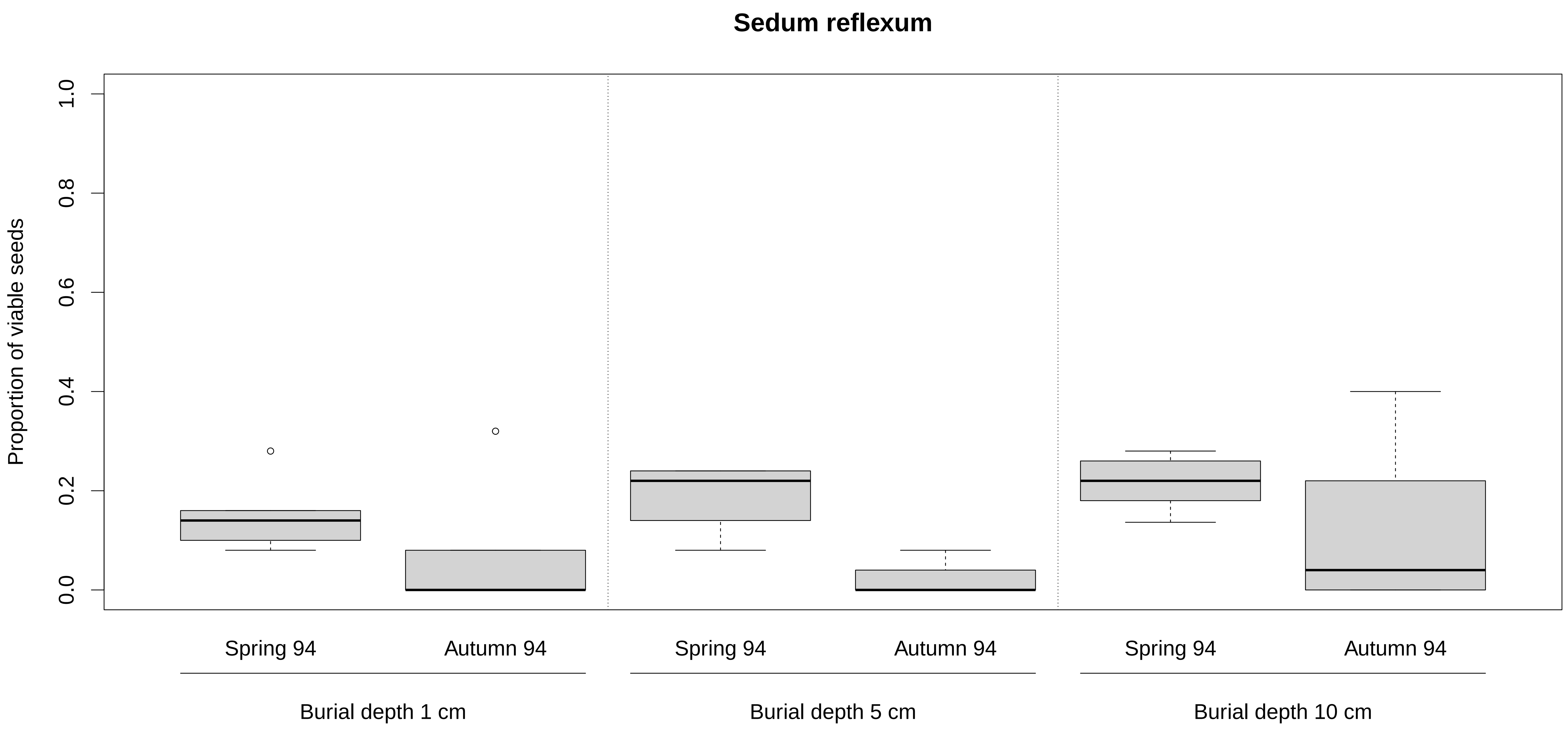

Sedum reflexum
Proportion of viable seeds
0.0
0.2
0.4
0.6
0.8
1.0
Spring 94
Autumn 94
Spring 94
Autumn 94
Spring 94
Autumn 94
Burial depth 1 cm
Burial depth 5 cm
Burial depth 10 cm
