# Supplementary material for: Soil Seed Bank Persistence Across Time and Burial Depth in Calcareous Grassland Habitats
Source: Front Plant Sci. 2022 Feb 4;12:790867. doi: 10.3389/fpls.2021.790867 (PMC8854790; doi:10.3389/fpls.2021.790867)
Supplement: Supplementary Figure 2 — The species-specific pattern in the proportion of dormant seeds during the time and different burial depths. [file Data_Sheet_2.PDF]

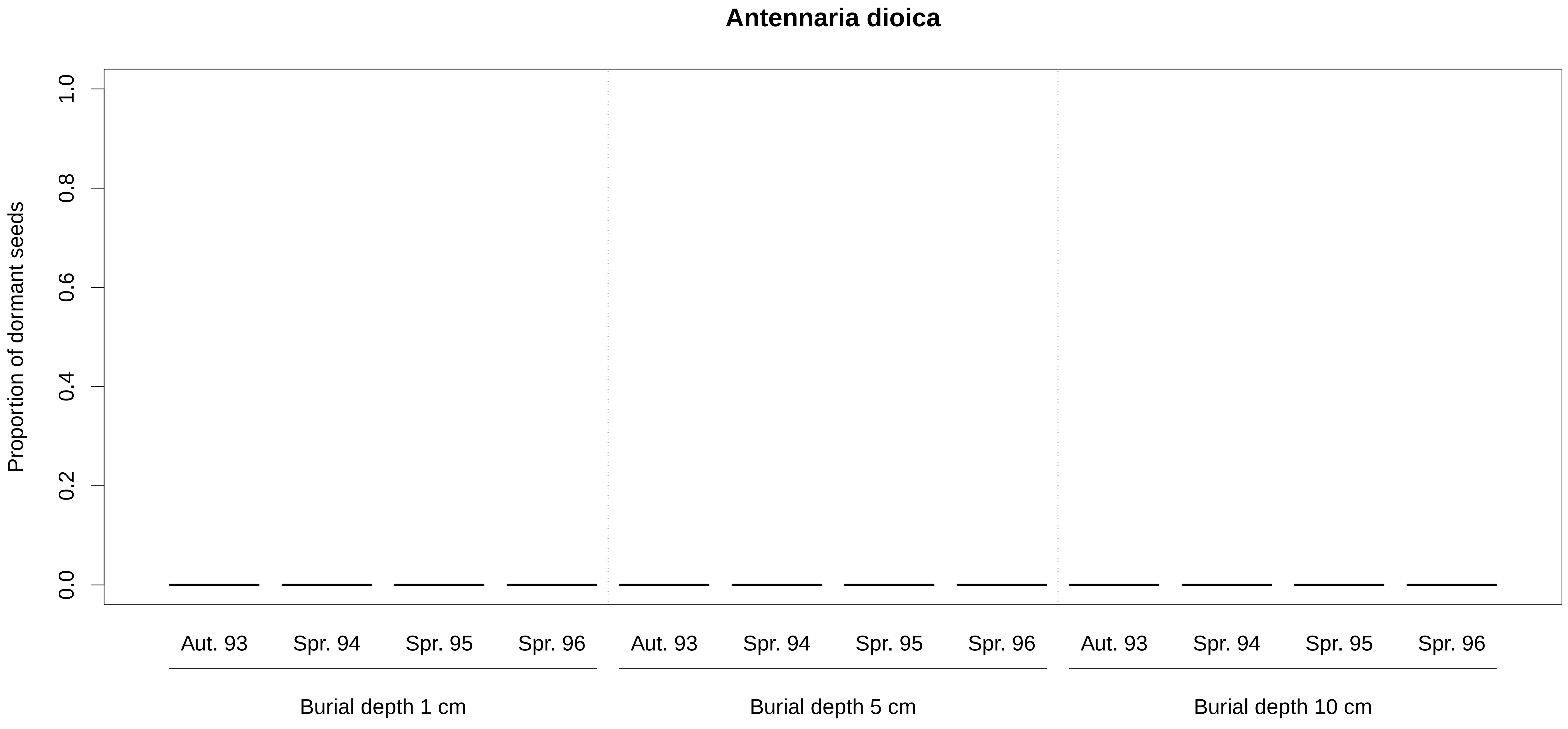
Antennaria dioica
Proportion of dormant seeds
0.0
0.2
0.4
0.6
0.8
1.0
Aut. 93
Spr. 94
Spr. 95
Spr. 96
Aut. 93
Spr. 94
Spr. 95
Spr. 96
Aut. 93
Spr. 94
Spr. 95
Spr. 96
Burial depth 1 cm
Burial depth 5 cm
Burial depth 10 cm

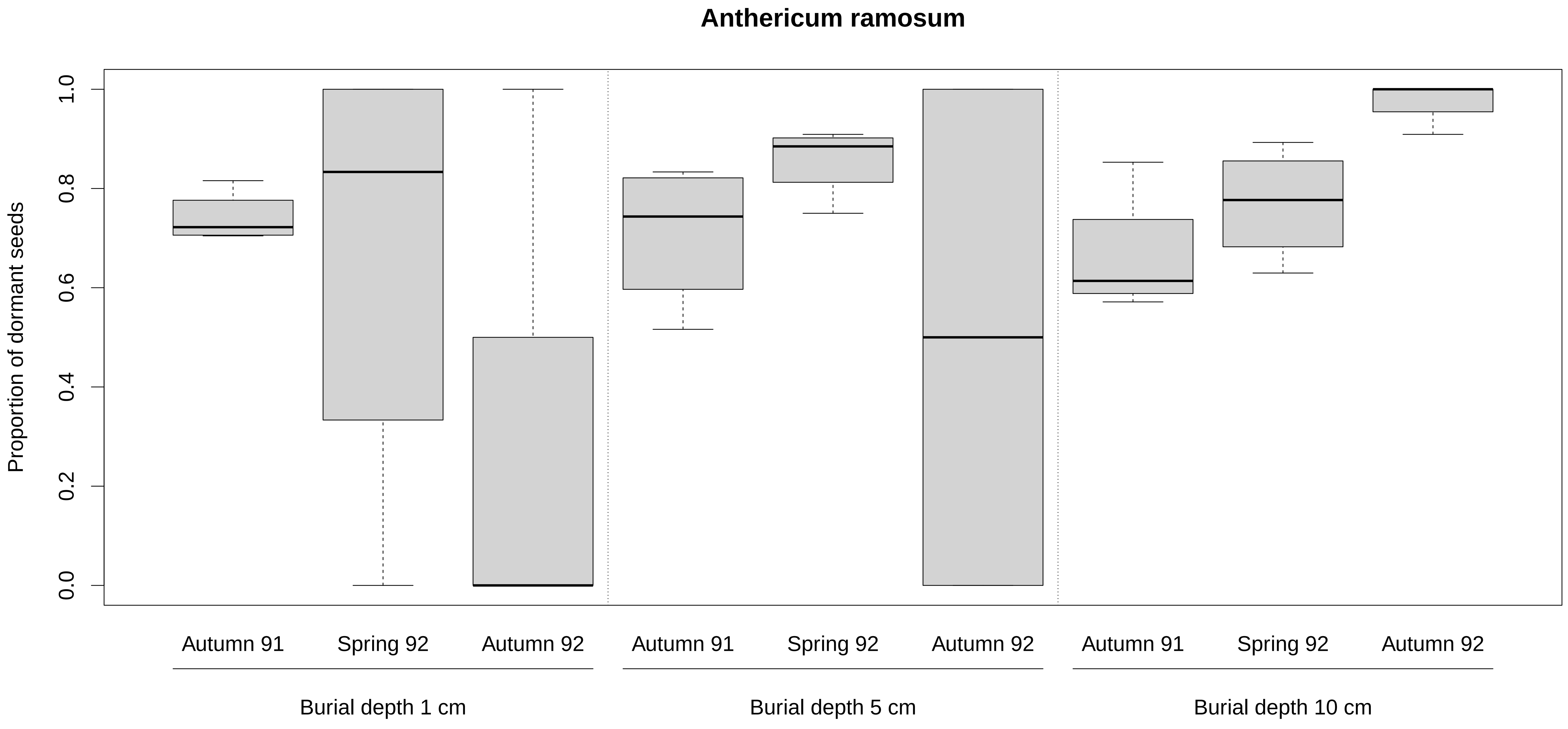
Anthericum ramosum
Proportion of dormant seeds
0.0
0.2
0.4
0.6
0.8
1.0
Autumn 91
Spring 92
Autumn 92
Autumn 91
Spring 92
Autumn 92
Autumn 91
Spring 92
Autumn 92
Burial depth 1 cm
Burial depth 5 cm
Burial depth 10 cm

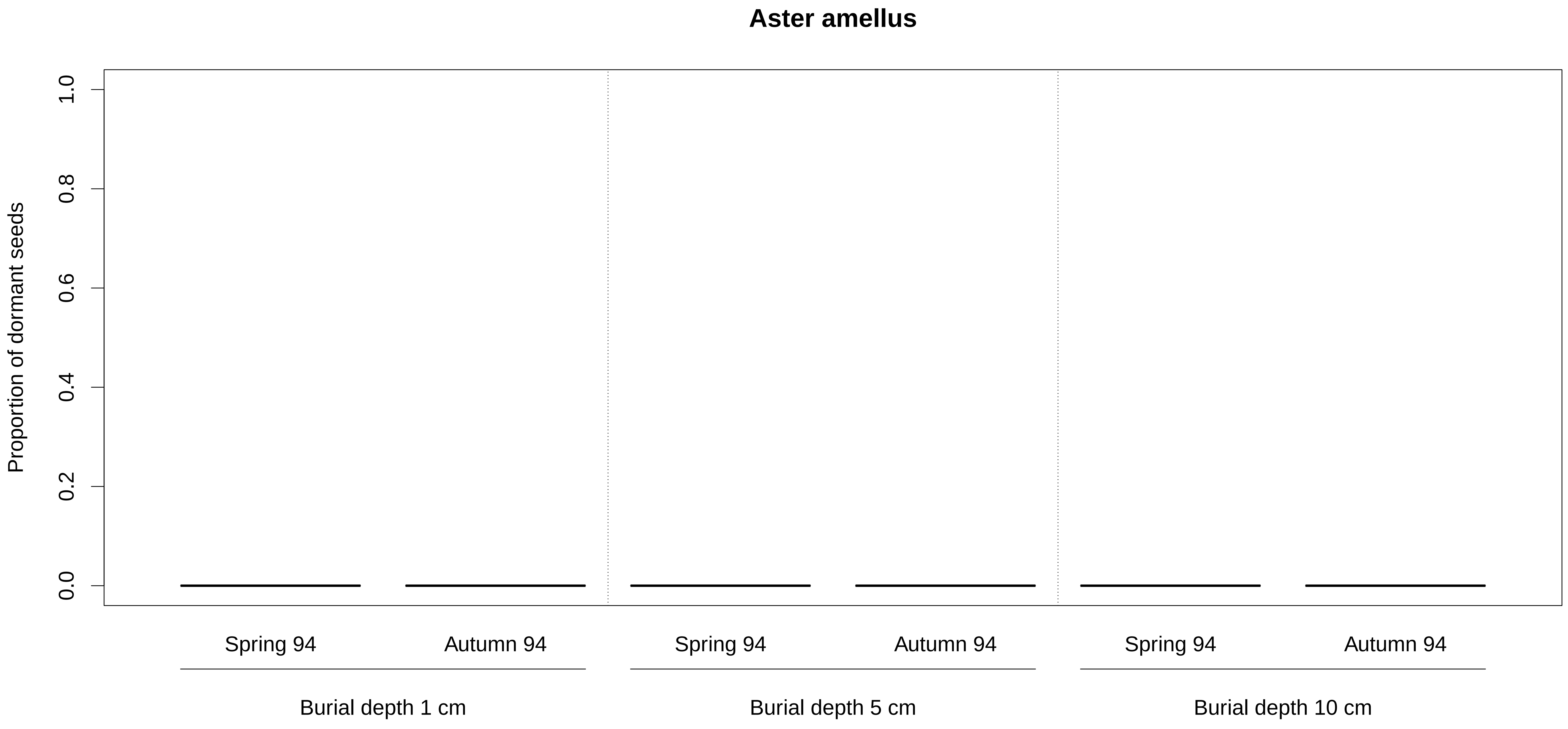
Aster amellus
Proportion of dormant seeds
0.0
0.2
0.4
0.6
0.8
1.0
Spring 94
Autumn 94
Spring 94
Autumn 94
Spring 94
Autumn 94
Burial depth 1 cm
Burial depth 5 cm
Burial depth 10 cm

Brachypodium pinnatum

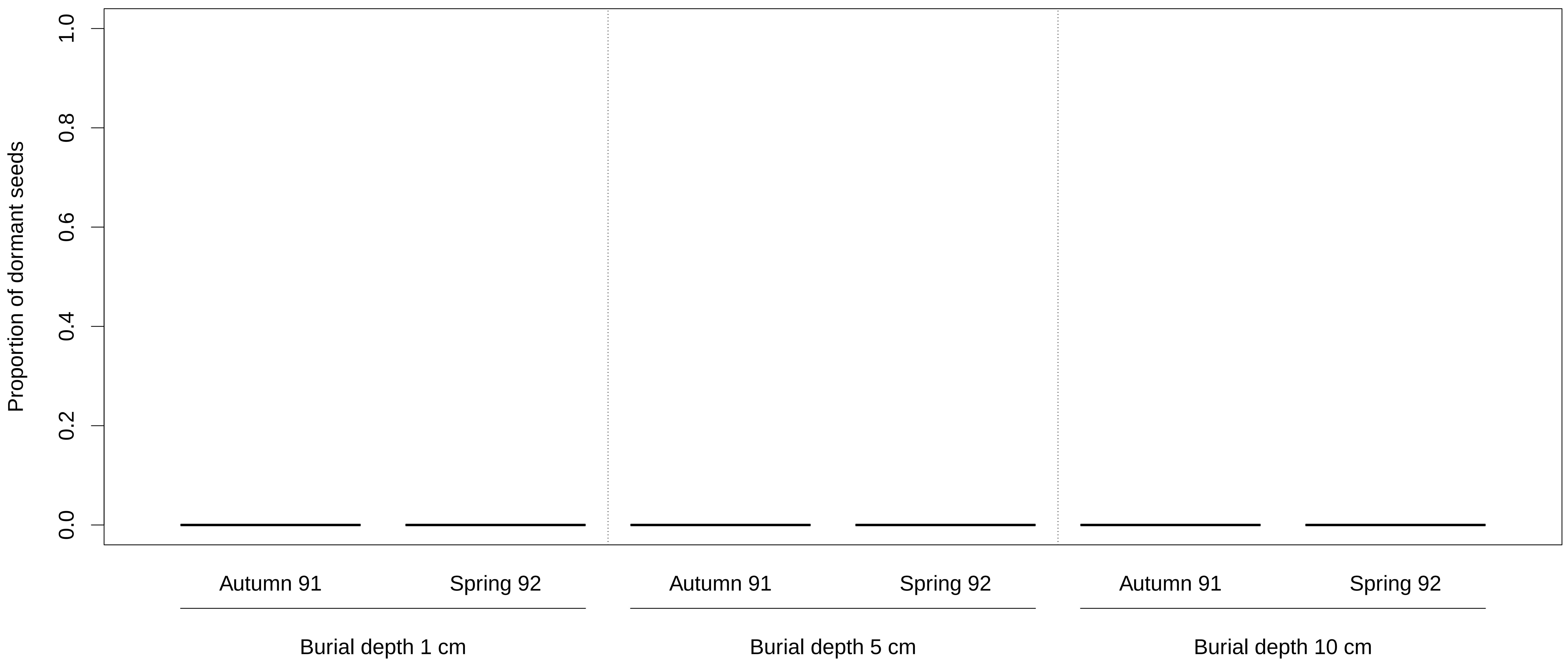

Bromus erectus

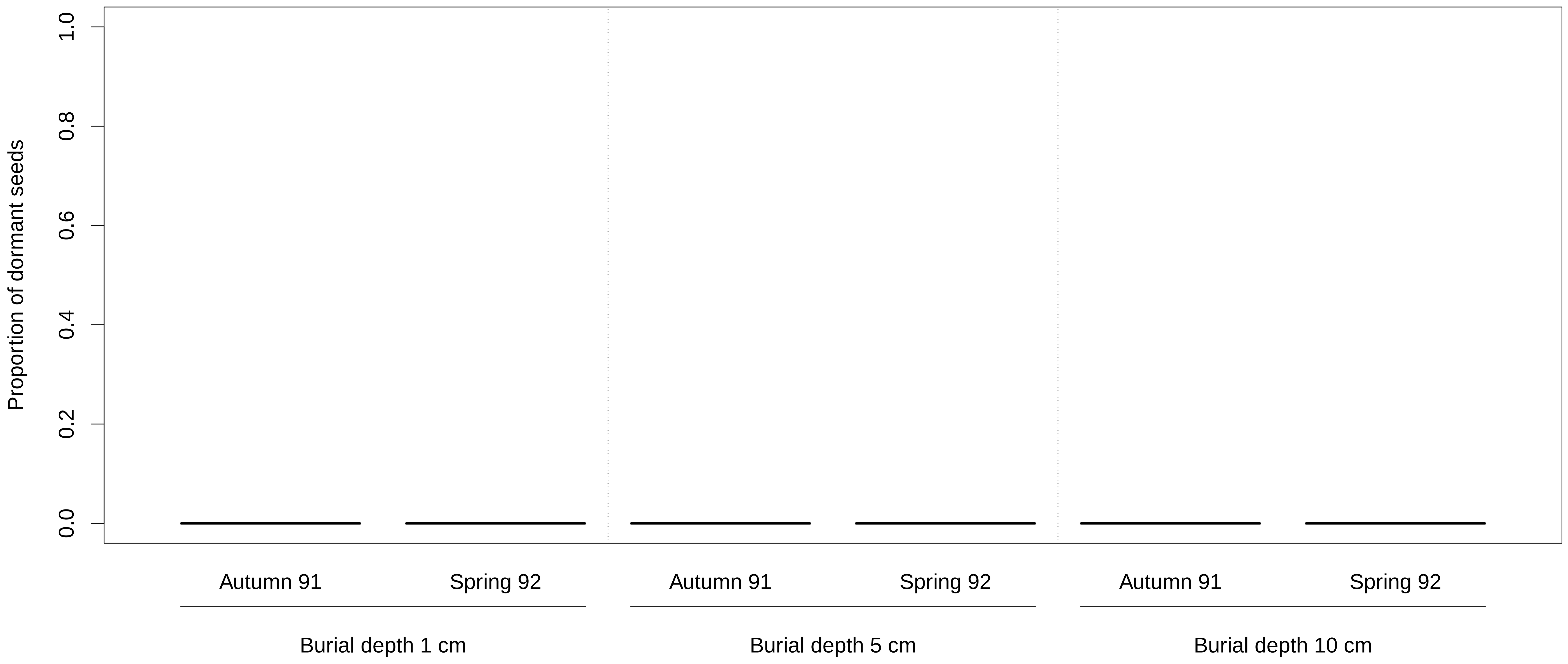

Bupleurum falcatum

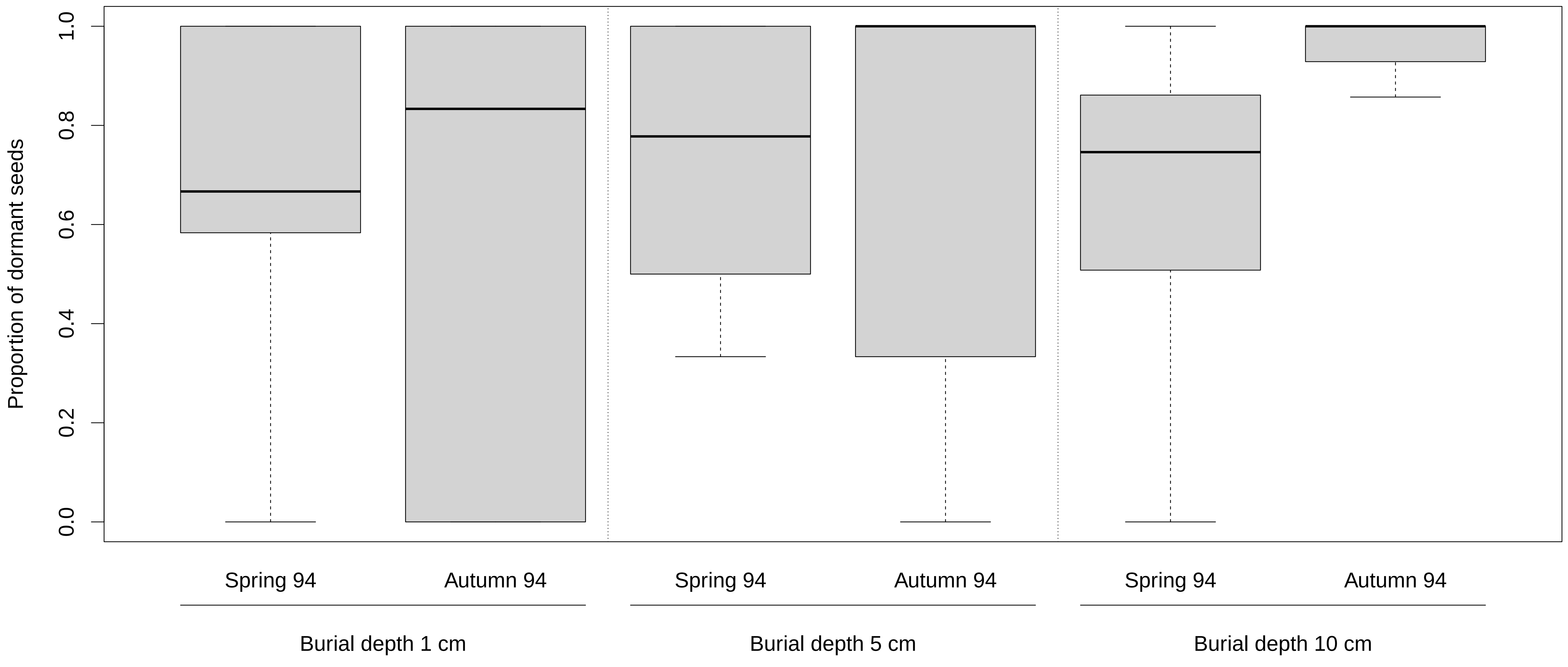

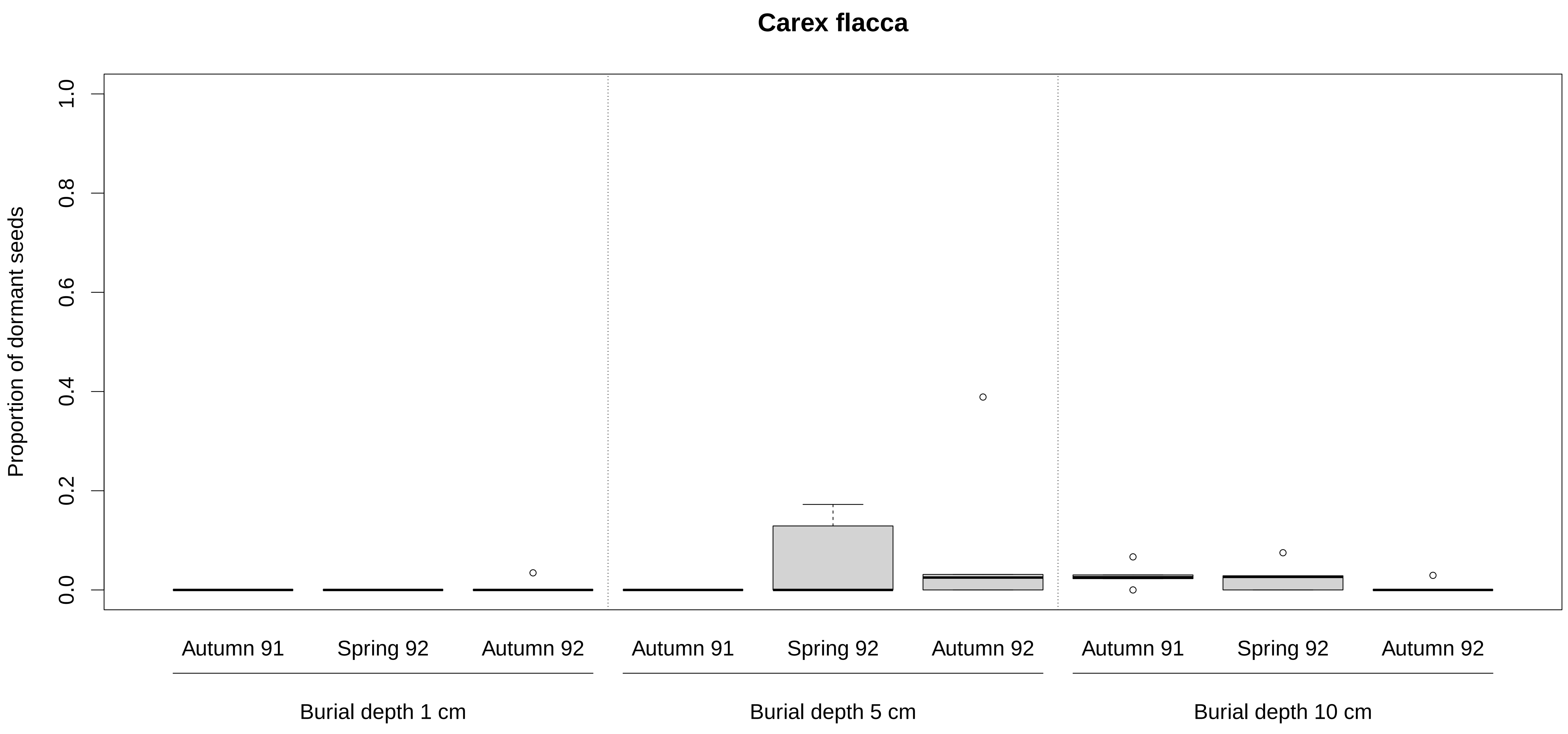
Carex flacca
Proportion of dormant seeds
0.0
0.2
0.4
0.6
0.8
1.0
Autumn 91
Spring 92
Autumn 92
Autumn 91
Spring 92
Autumn 92
Autumn 91
Spring 92
Autumn 92
Burial depth 1 cm
Burial depth 5 cm
Burial depth 10 cm

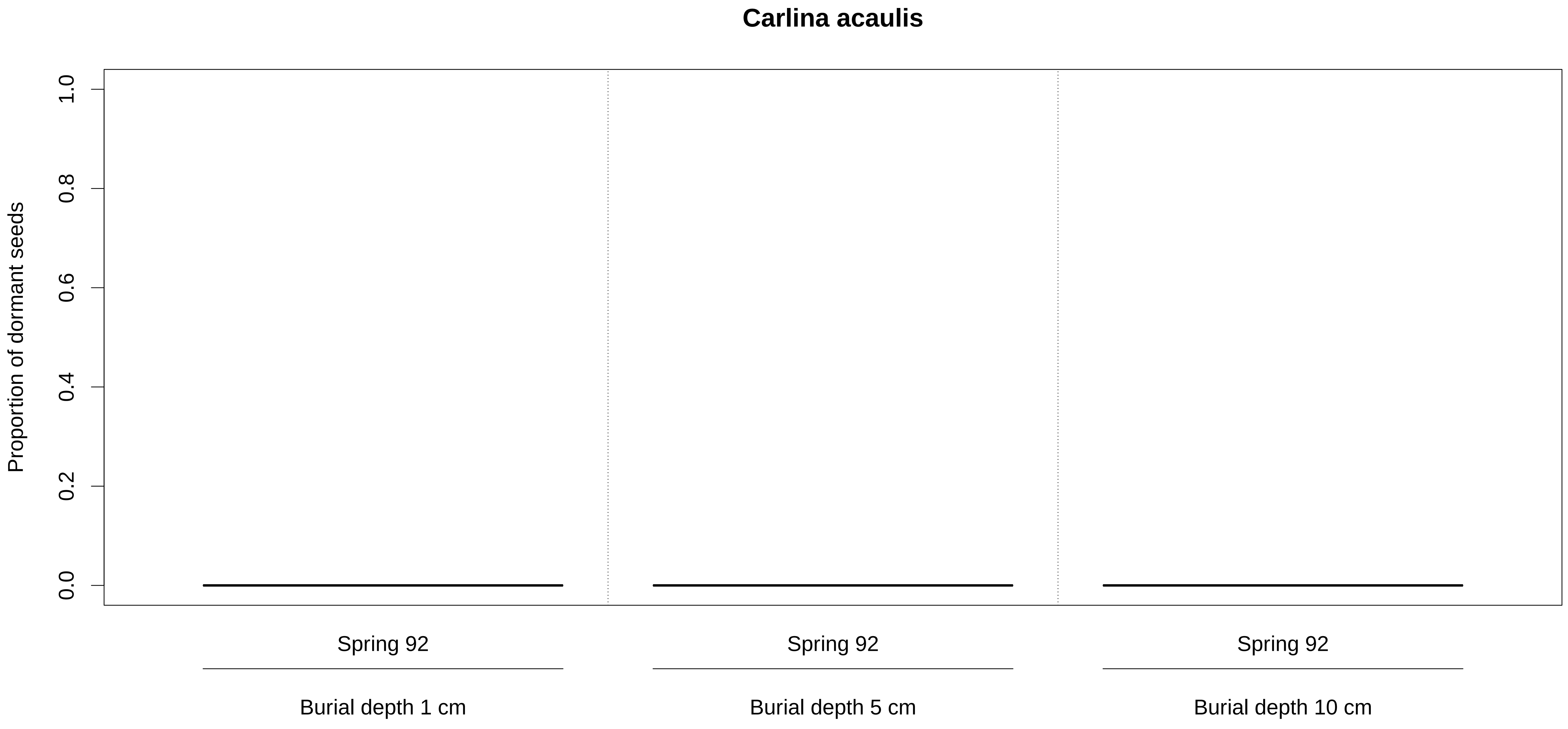
Carlina acaulis
Proportion of dormant seeds
0.0
0.2
0.4
0.6
0.8
1.0
Spring 92
Spring 92
Spring 92
Burial depth 1 cm
Burial depth 5 cm
Burial depth 10 cm

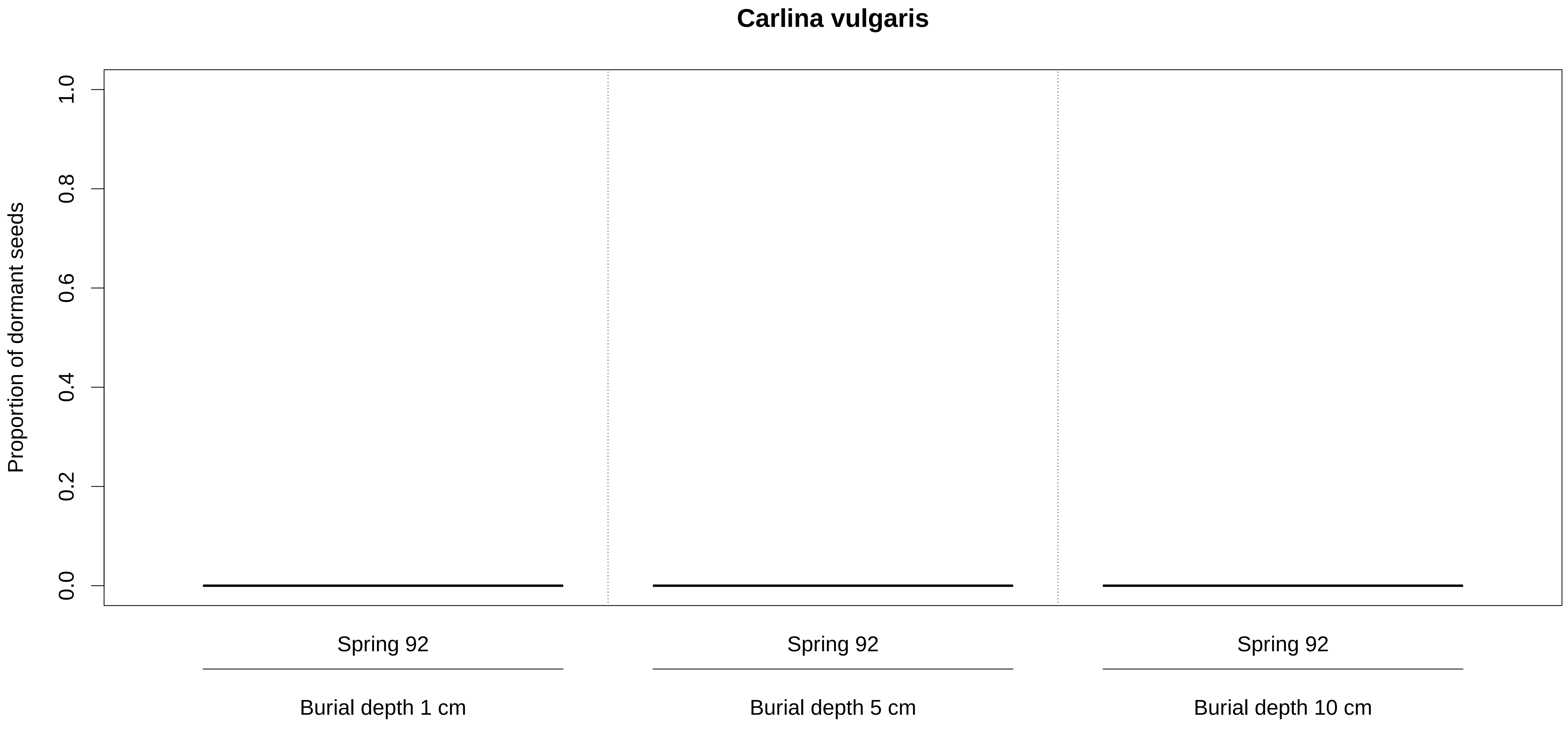
Carlina vulgaris
Proportion of dormant seeds
0.0
0.2
0.4
0.6
0.8
1.0
Spring 92
Spring 92
Spring 92
Burial depth 1 cm
Burial depth 5 cm
Burial depth 10 cm

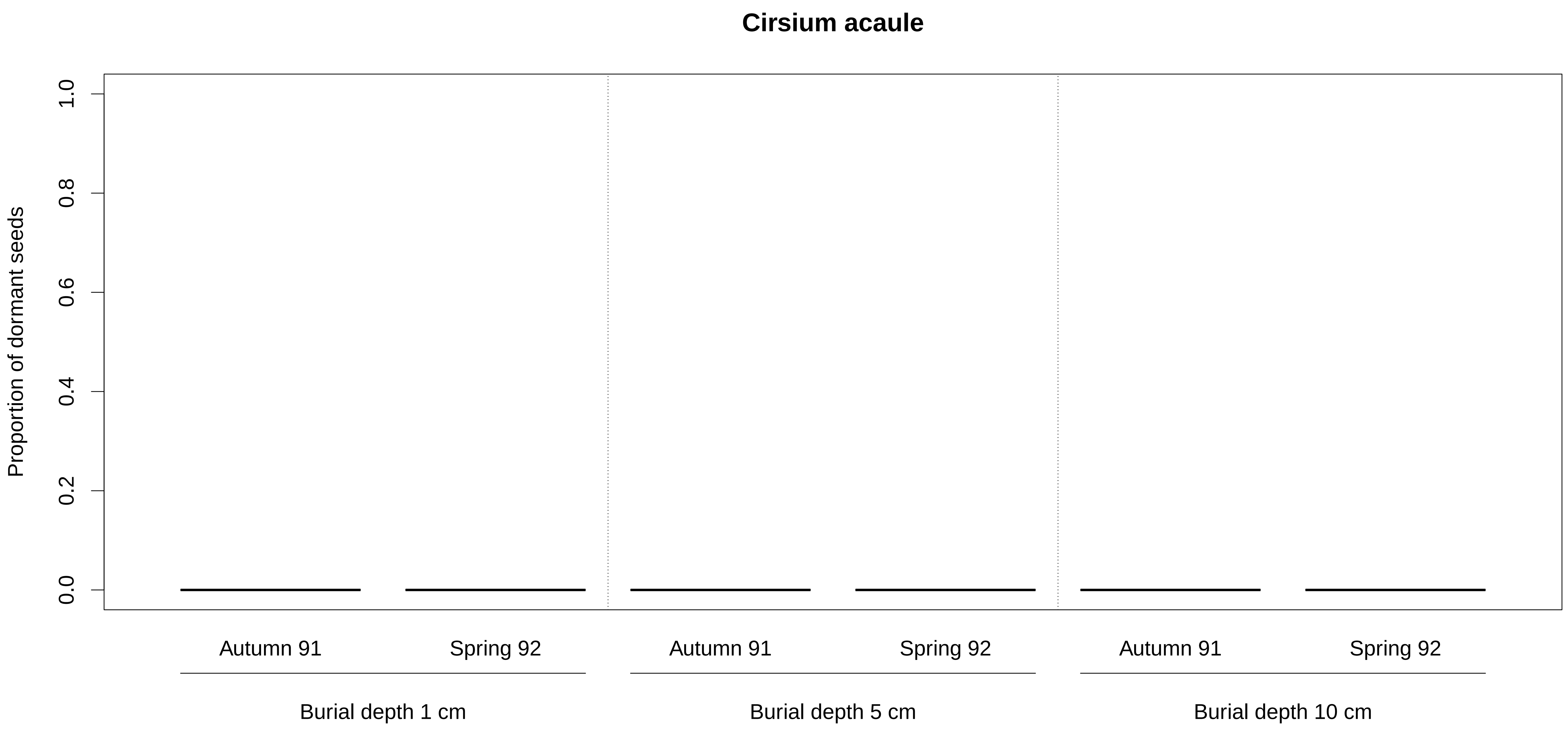
Cirsium acaule
Proportion of dormant seeds
0.0
0.2
0.4
0.6
0.8
1.0
Autumn 91
Spring 92
Autumn 91
Spring 92
Autumn 91
Spring 92
Burial depth 1 cm
Burial depth 5 cm
Burial depth 10 cm

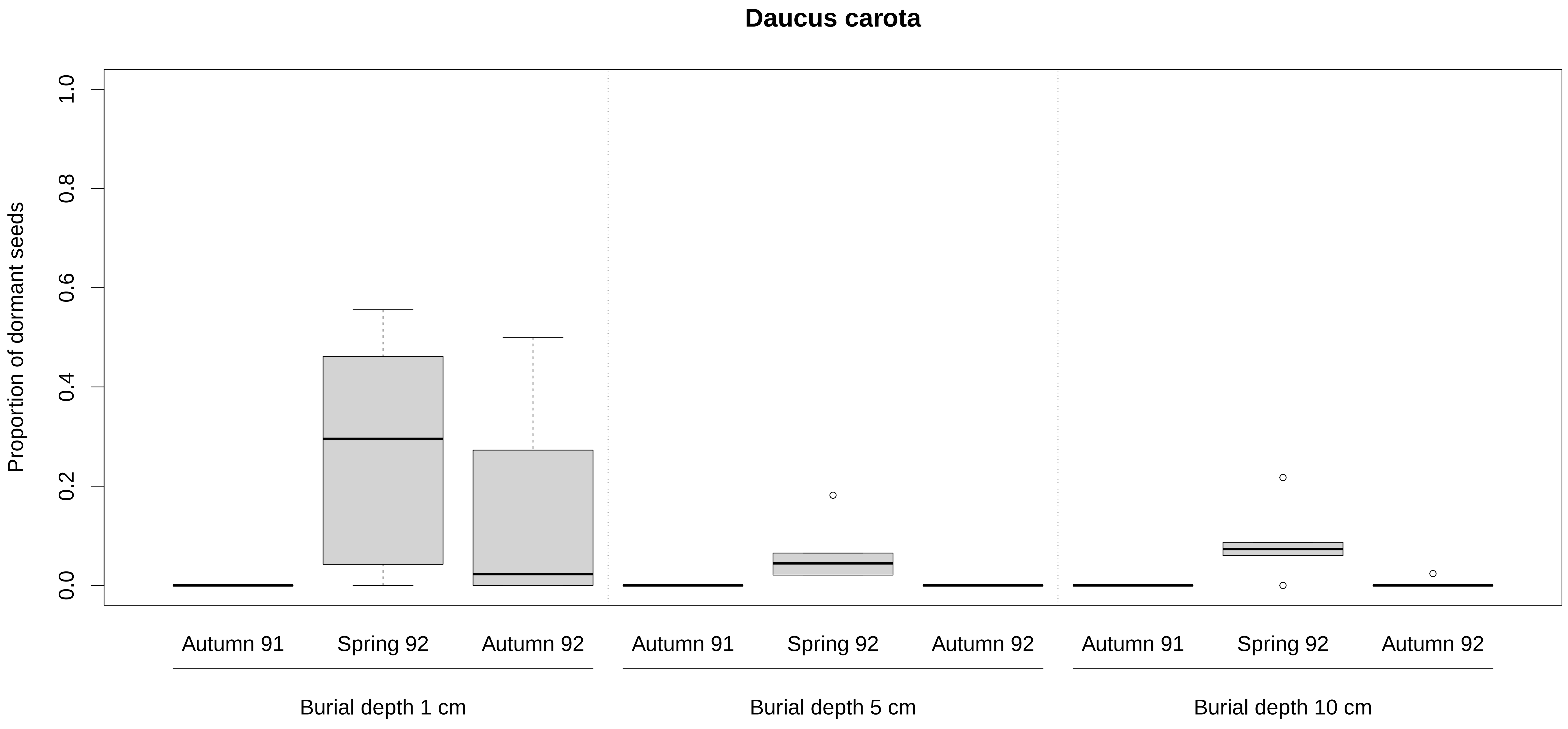
Daucus carota
Proportion of dormant seeds
0.0
0.2
0.4
0.6
0.8
1.0
Autumn 91
Spring 92
Autumn 92
Autumn 91
Spring 92
Autumn 92
Autumn 91
Spring 92
Autumn 92
Burial depth 1 cm
Burial depth 5 cm
Burial depth 10 cm

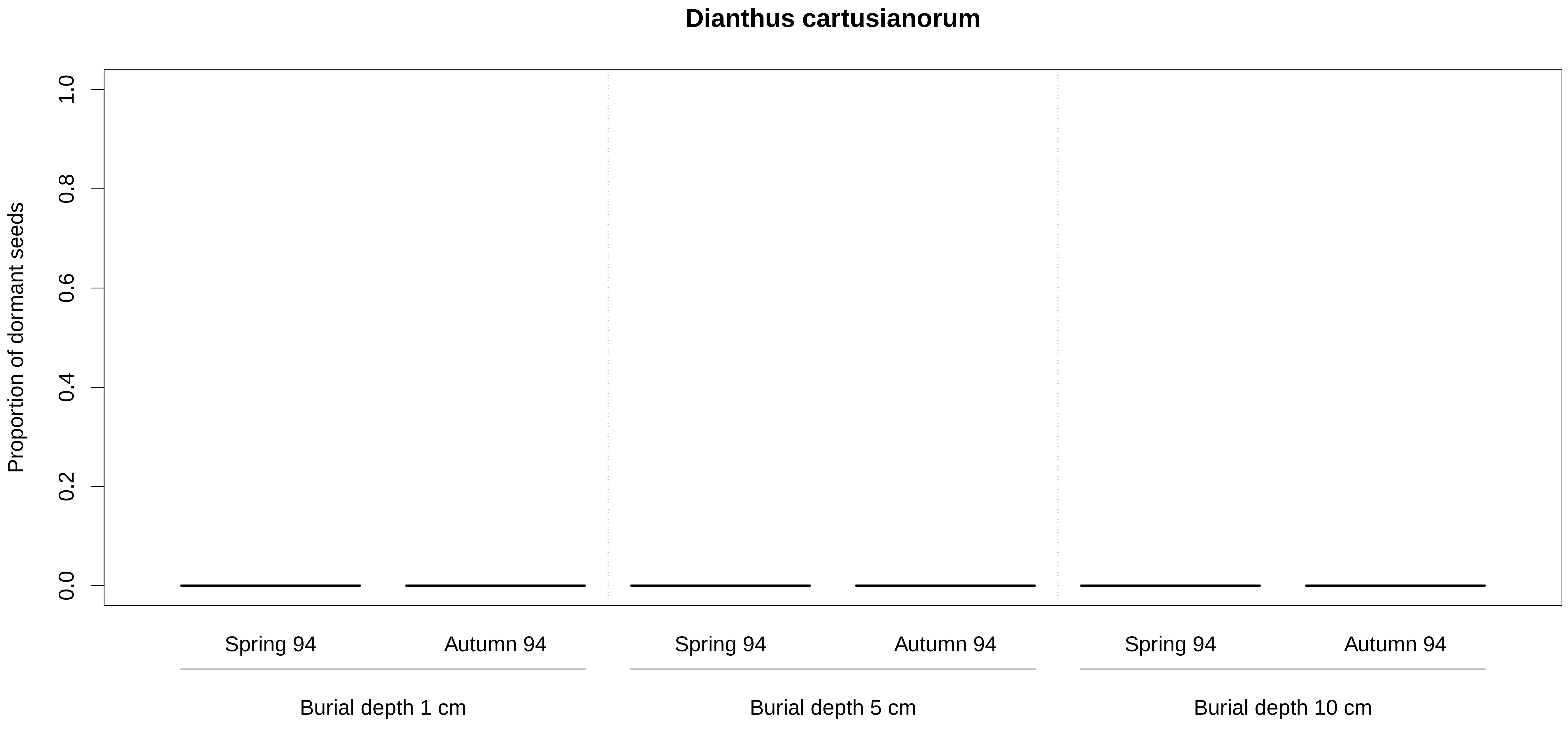
Dianthus cartusianorum
Proportion of dormant seeds
0.0
0.2
0.4
0.6
0.8
1.0
Spring 94
Autumn 94
Spring 94
Autumn 94
Spring 94
Autumn 94
Burial depth 1 cm
Burial depth 5 cm
Burial depth 10 cm

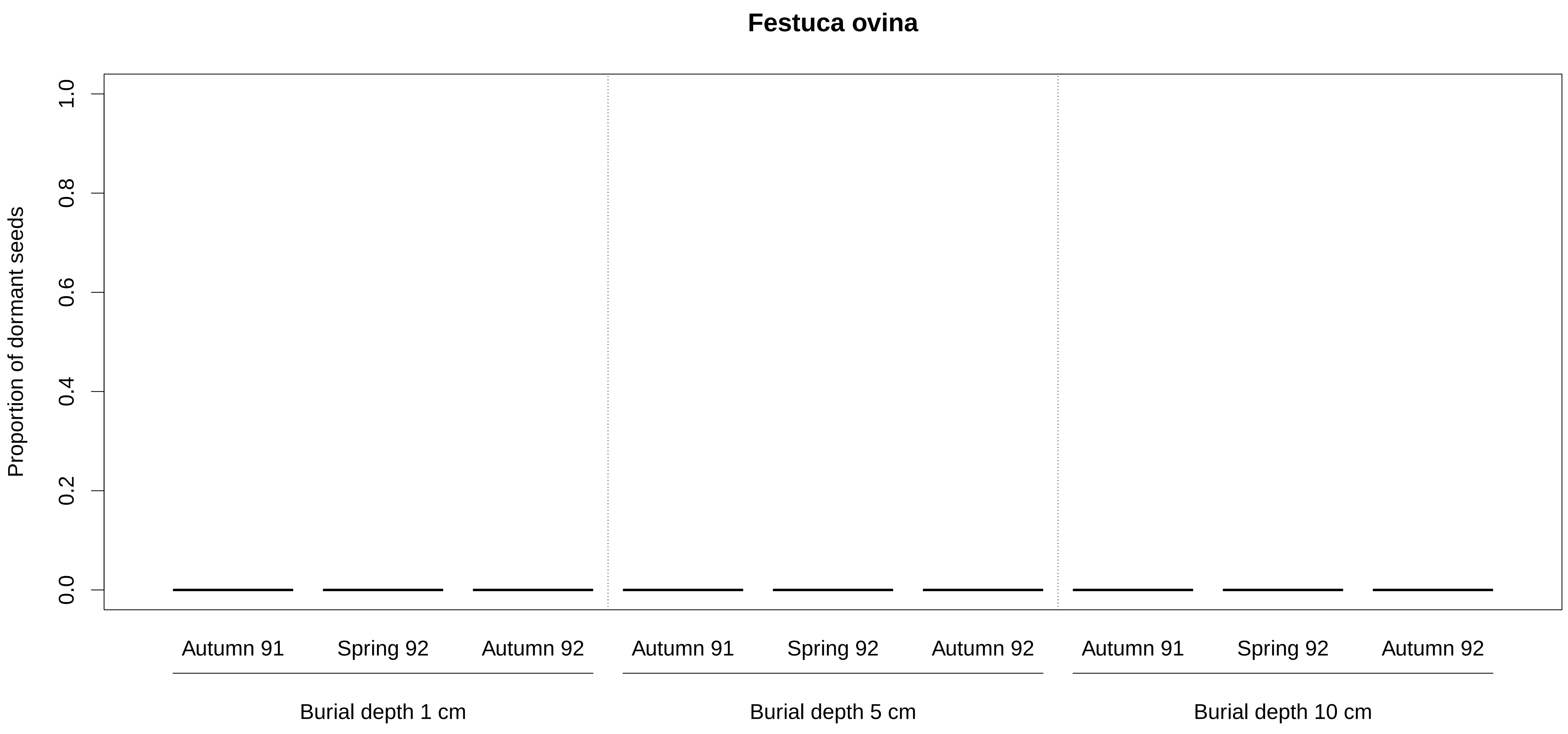
Festuca ovina
Proportion of dormant seeds
0.0
0.2
0.4
0.6
0.8
1.0
Autumn 91
Spring 92
Autumn 92
Burial depth 1 cm
Burial depth 5 cm
Burial depth 10 cm

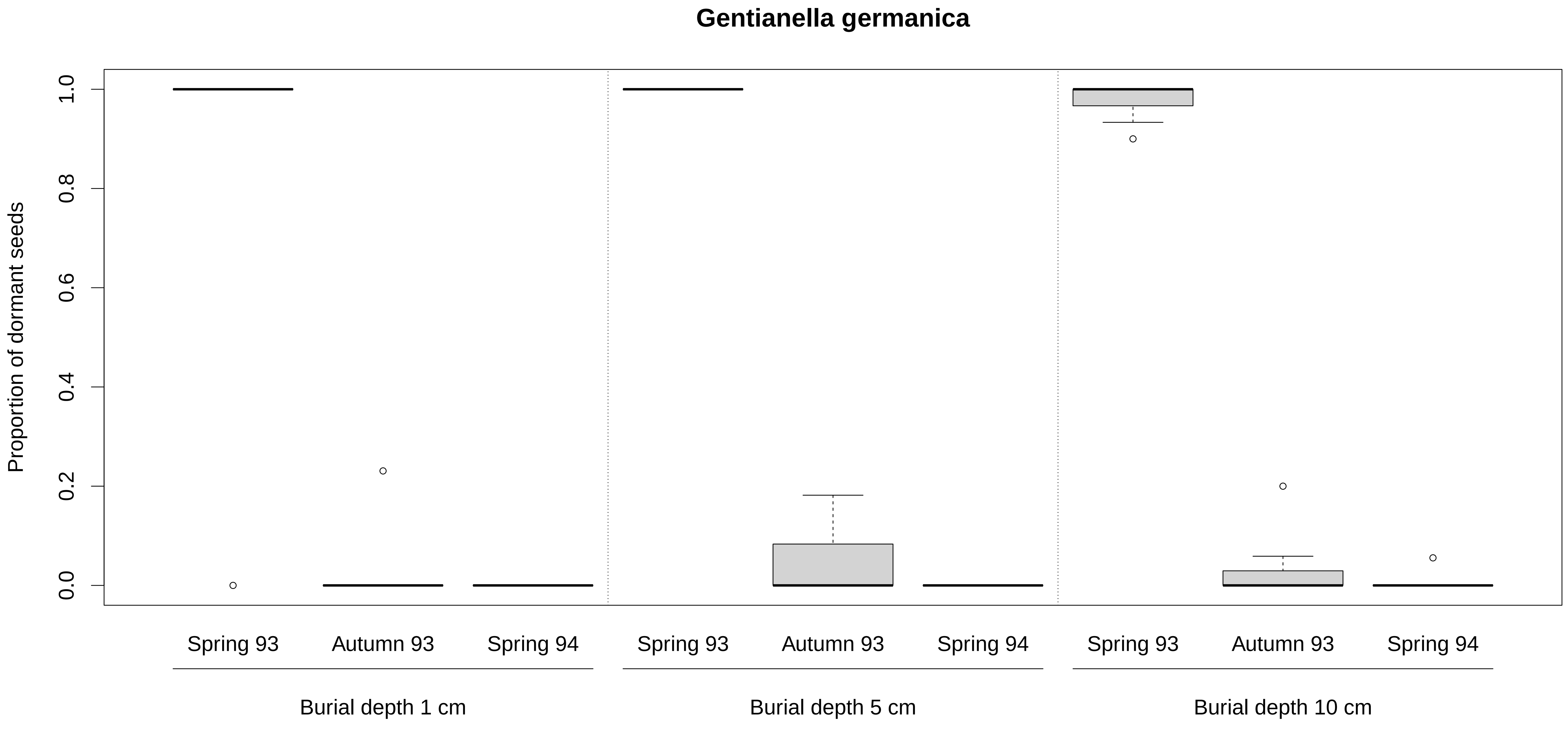
Gentianella germanica
Proportion of dormant seeds
0.0
0.2
0.4
0.6
0.8
1.0
Spring 93
Autumn 93
Spring 94
Burial depth 1 cm
Burial depth 5 cm
Burial depth 10 cm

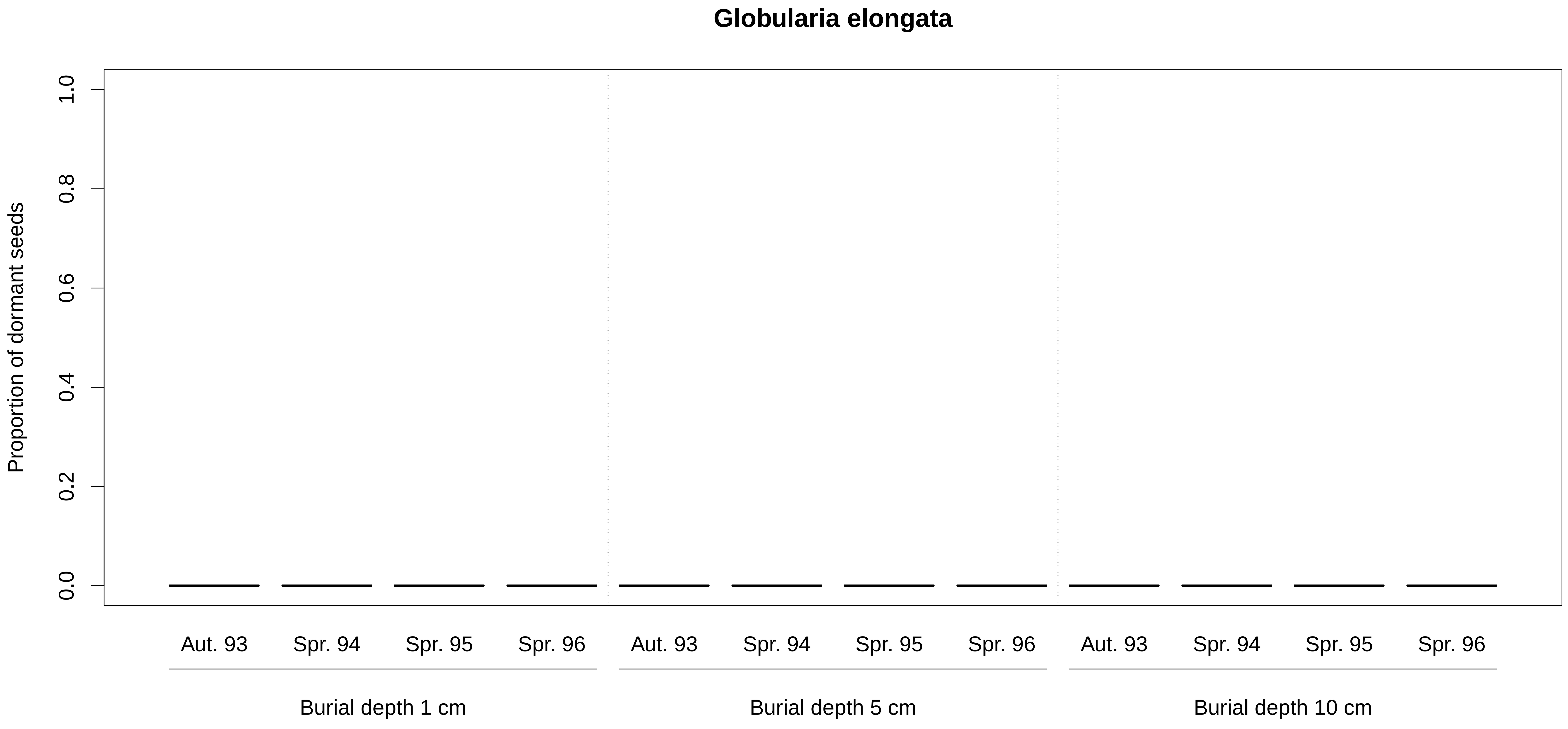
Globularia elongata
Proportion of dormant seeds
0.0
0.2
0.4
0.6
0.8
1.0
Aut. 93
Spr. 94
Spr. 95
Spr. 96
Burial depth 1 cm
Burial depth 5 cm
Burial depth 10 cm

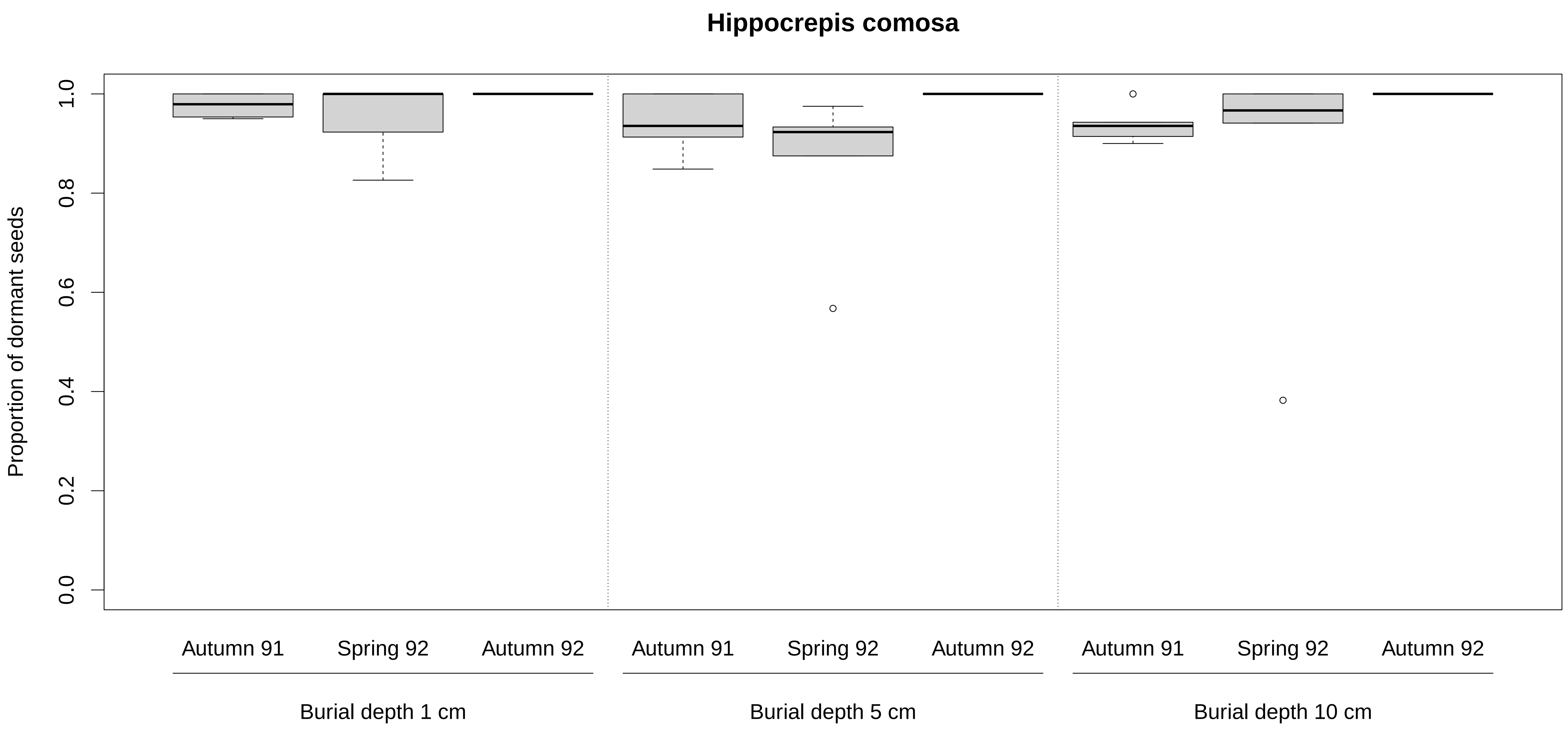
Hippocrepis comosa
Proportion of dormant seeds
0.0
0.2
0.4
0.6
0.8
1.0
Autumn 91
Spring 92
Autumn 92
Autumn 91
Spring 92
Autumn 92
Autumn 91
Spring 92
Autumn 92
Burial depth 1 cm
Burial depth 5 cm
Burial depth 10 cm

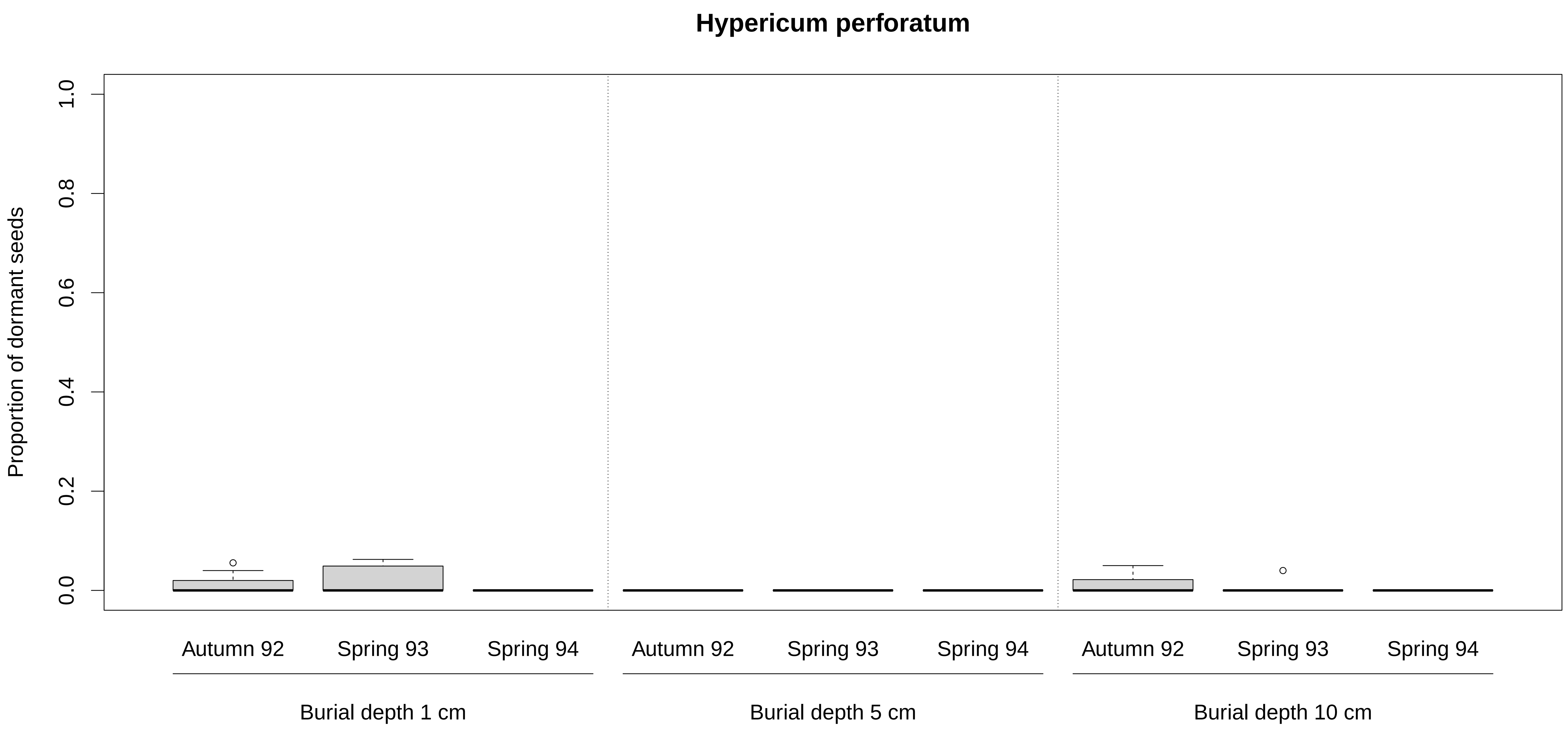
Hypericum perforatum
Proportion of dormant seeds
0.0
0.2
0.4
0.6
0.8
1.0
Autumn 92
Spring 93
Spring 94
Autumn 92
Spring 93
Spring 94
Autumn 92
Spring 93
Spring 94
Burial depth 1 cm
Burial depth 5 cm
Burial depth 10 cm

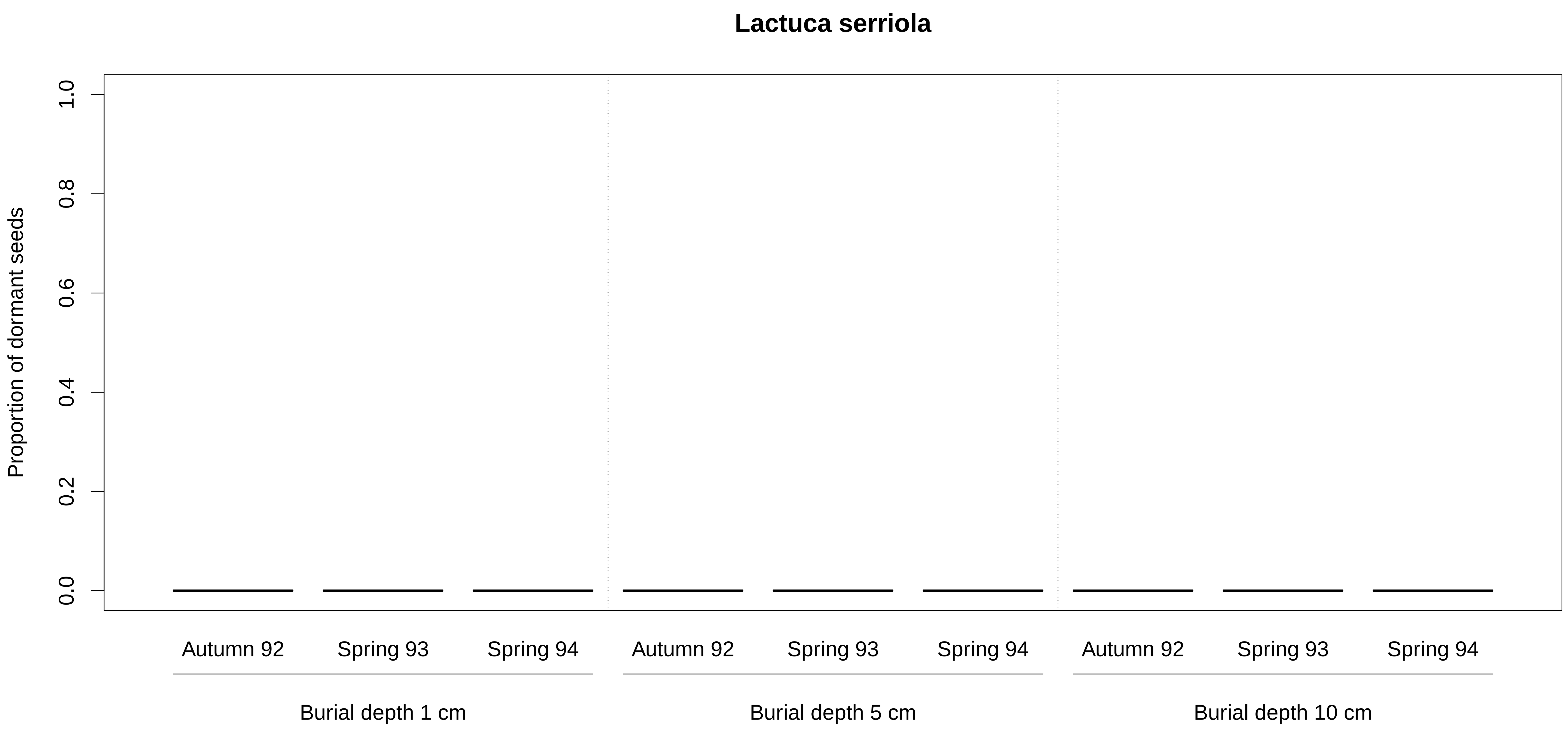
Lactuca serriola
Proportion of dormant seeds
0.0
0.2
0.4
0.6
0.8
1.0
Autumn 92
Spring 93
Spring 94
Autumn 92
Spring 93
Spring 94
Autumn 92
Spring 93
Spring 94
Burial depth 1 cm
Burial depth 5 cm
Burial depth 10 cm

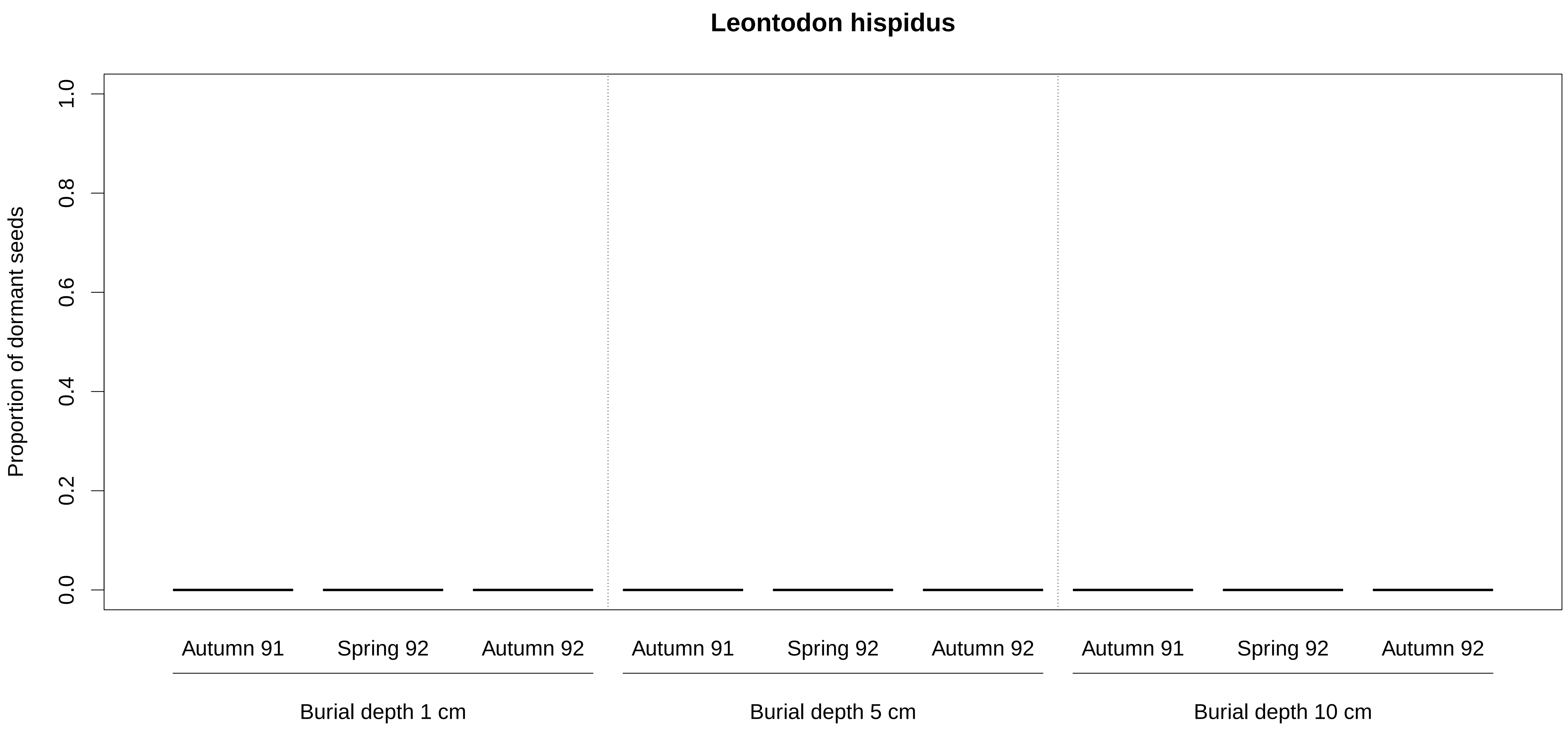
Leontodon hispidus
Proportion of dormant seeds
0.0
0.2
0.4
0.6
0.8
1.0
Autumn 91
Spring 92
Autumn 92
Autumn 91
Spring 92
Autumn 92
Autumn 91
Spring 92
Autumn 92
Burial depth 1 cm
Burial depth 5 cm
Burial depth 10 cm

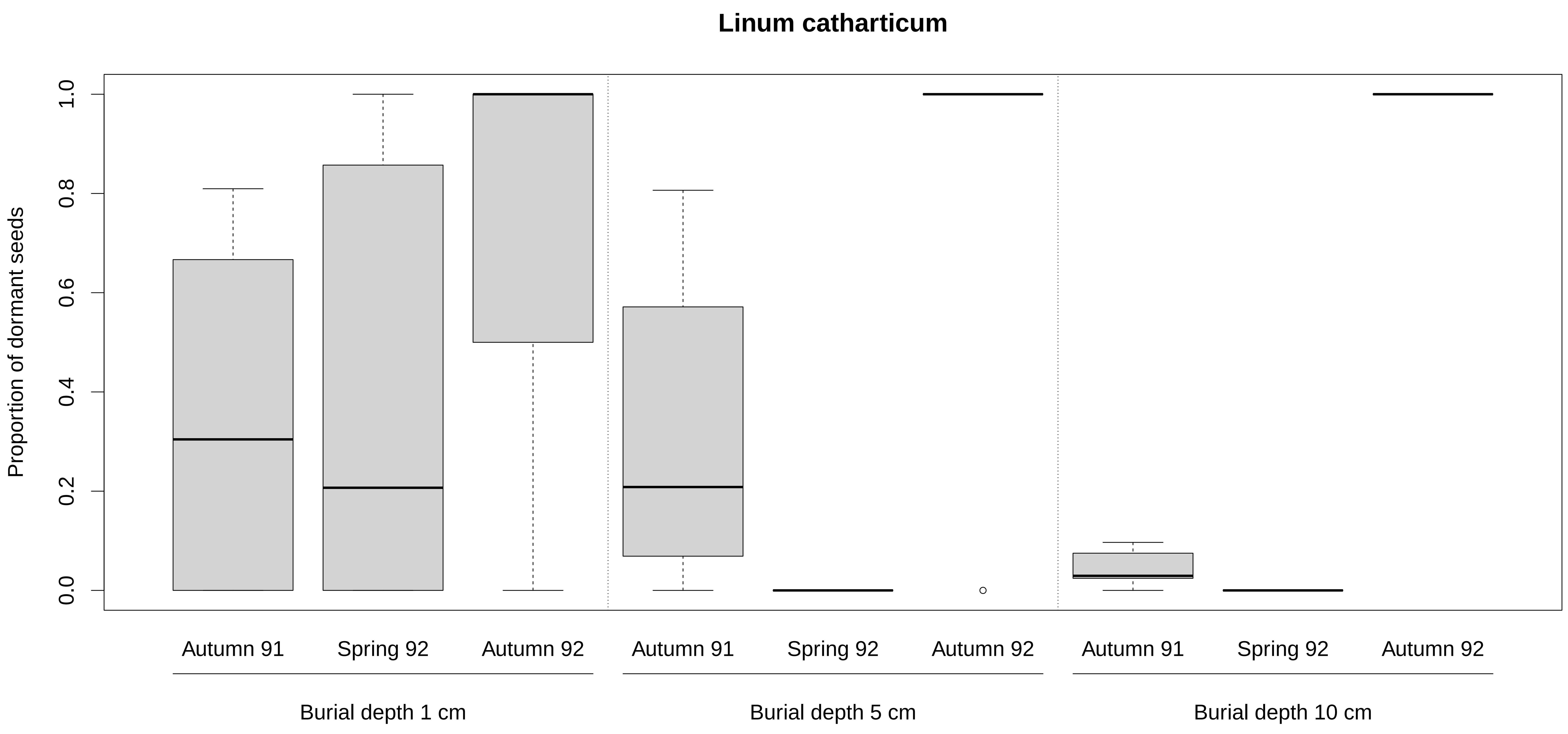
Linum catharticum
Proportion of dormant seeds
0.0
0.2
0.4
0.6
0.8
1.0
Autumn 91
Spring 92
Autumn 92
Autumn 91
Spring 92
Autumn 92
Autumn 91
Spring 92
Autumn 92
Burial depth 1 cm
Burial depth 5 cm
Burial depth 10 cm

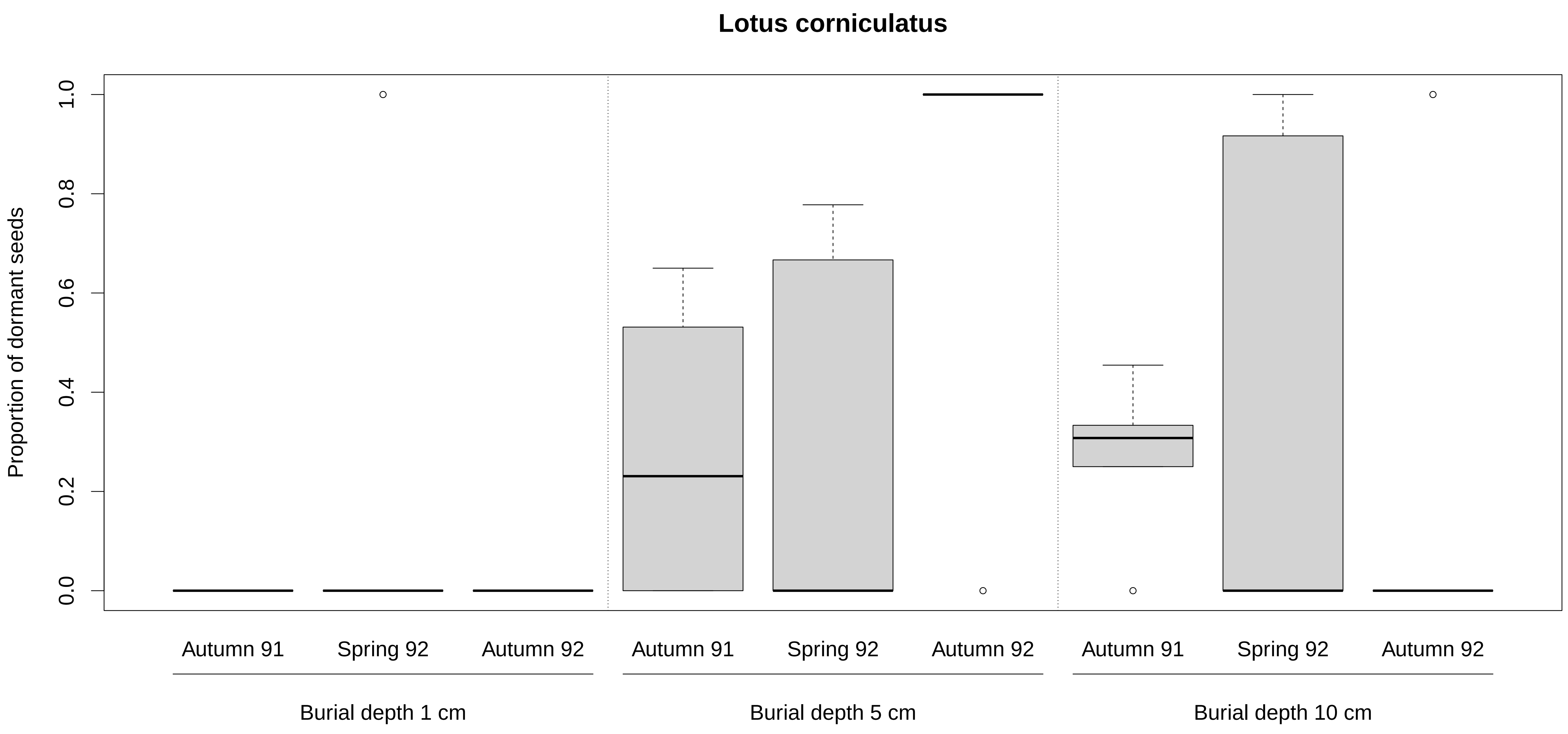
Lotus corniculatus
Proportion of dormant seeds
0.0
0.2
0.4
0.6
0.8
1.0
Autumn 91
Spring 92
Autumn 92
Autumn 91
Spring 92
Autumn 92
Autumn 91
Spring 92
Autumn 92
Burial depth 1 cm
Burial depth 5 cm
Burial depth 10 cm

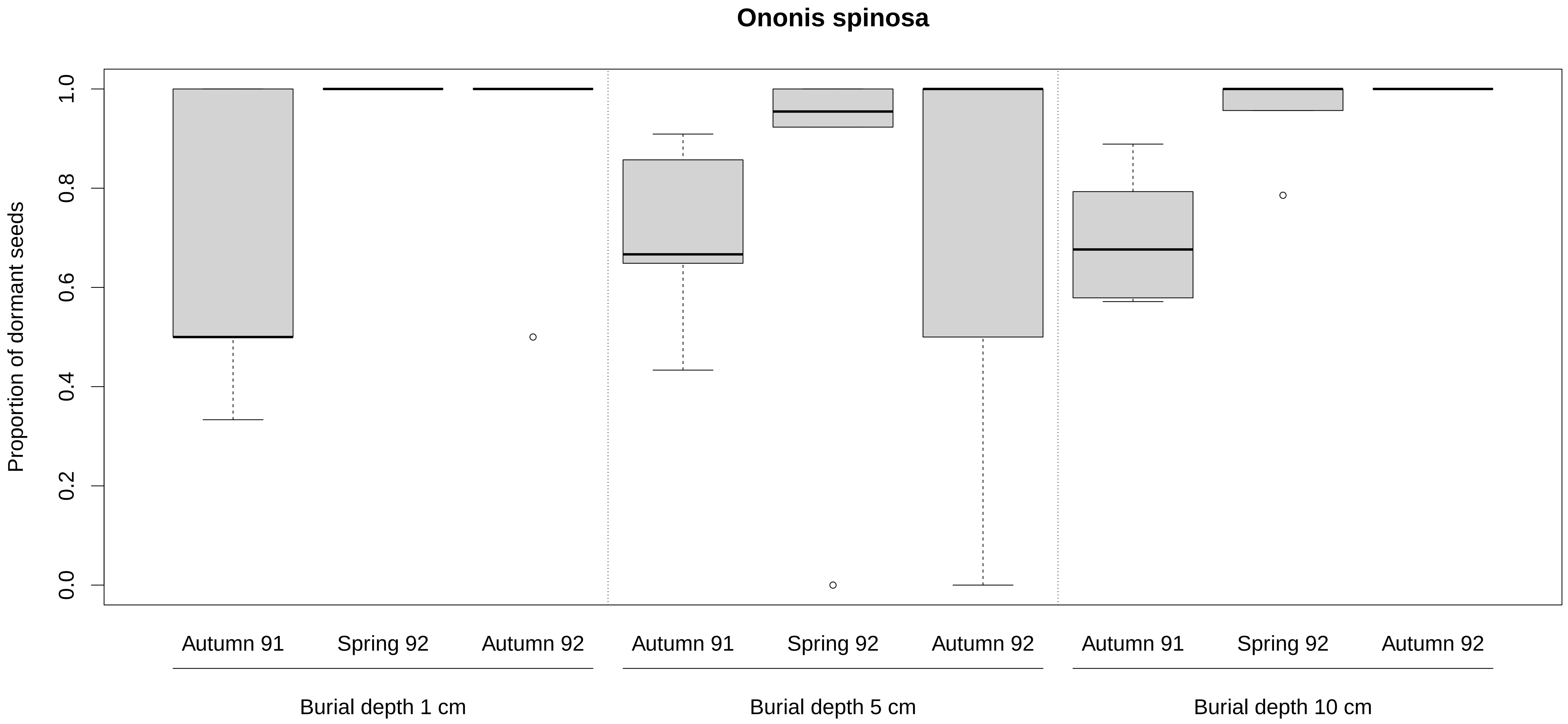

Ononis spinosa
Proportion of dormant seeds
0.0
0.2
0.4
0.6
0.8
1.0
Autumn 91
Spring 92
Autumn 92
Autumn 91
Spring 92
Autumn 92
Autumn 91
Spring 92
Autumn 92
Burial depth 1 cm
Burial depth 5 cm
Burial depth 10 cm

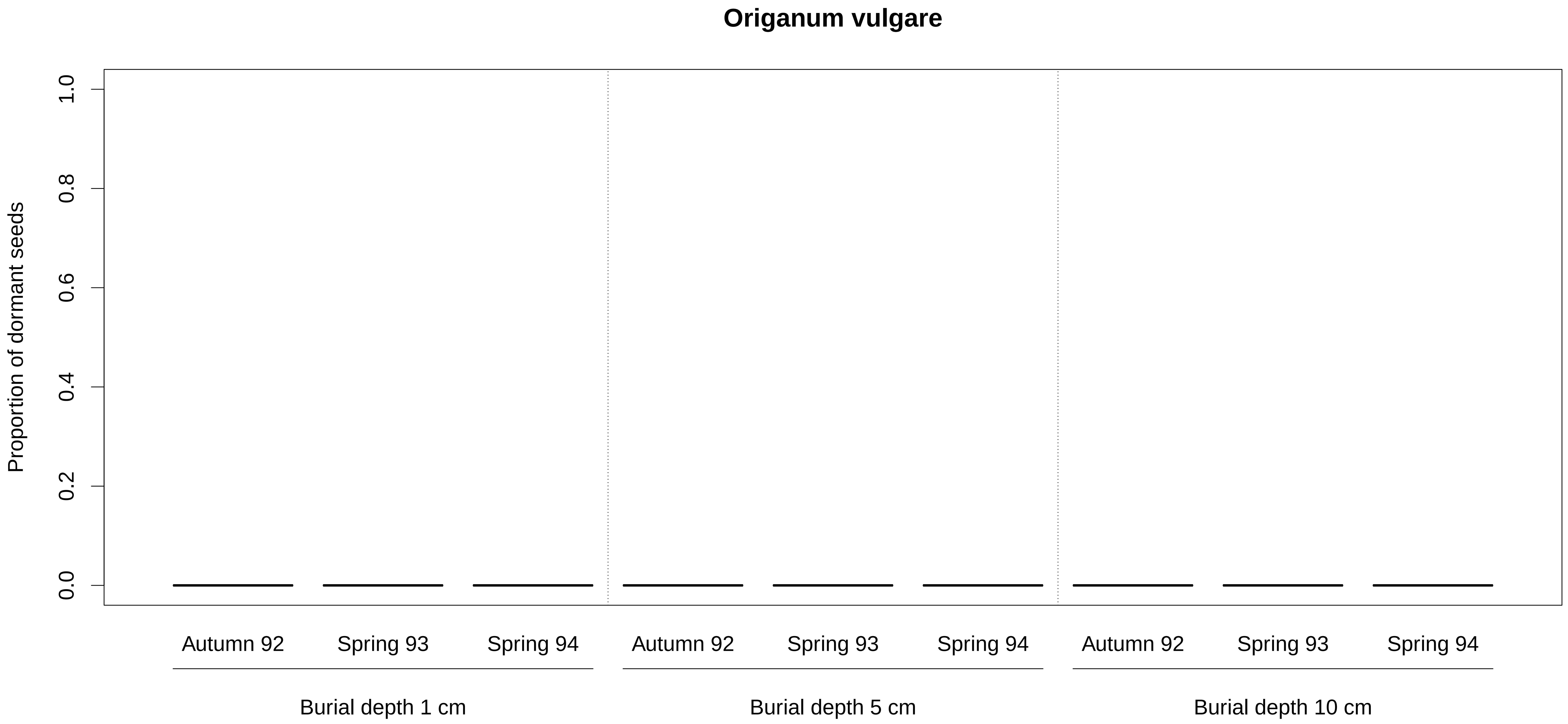

Origanum vulgare
Proportion of dormant seeds
0.0
0.2
0.4
0.6
0.8
1.0
Autumn 92
Spring 93
Spring 94
Autumn 92
Spring 93
Spring 94
Autumn 92
Spring 93
Spring 94
Burial depth 1 cm
Burial depth 5 cm
Burial depth 10 cm

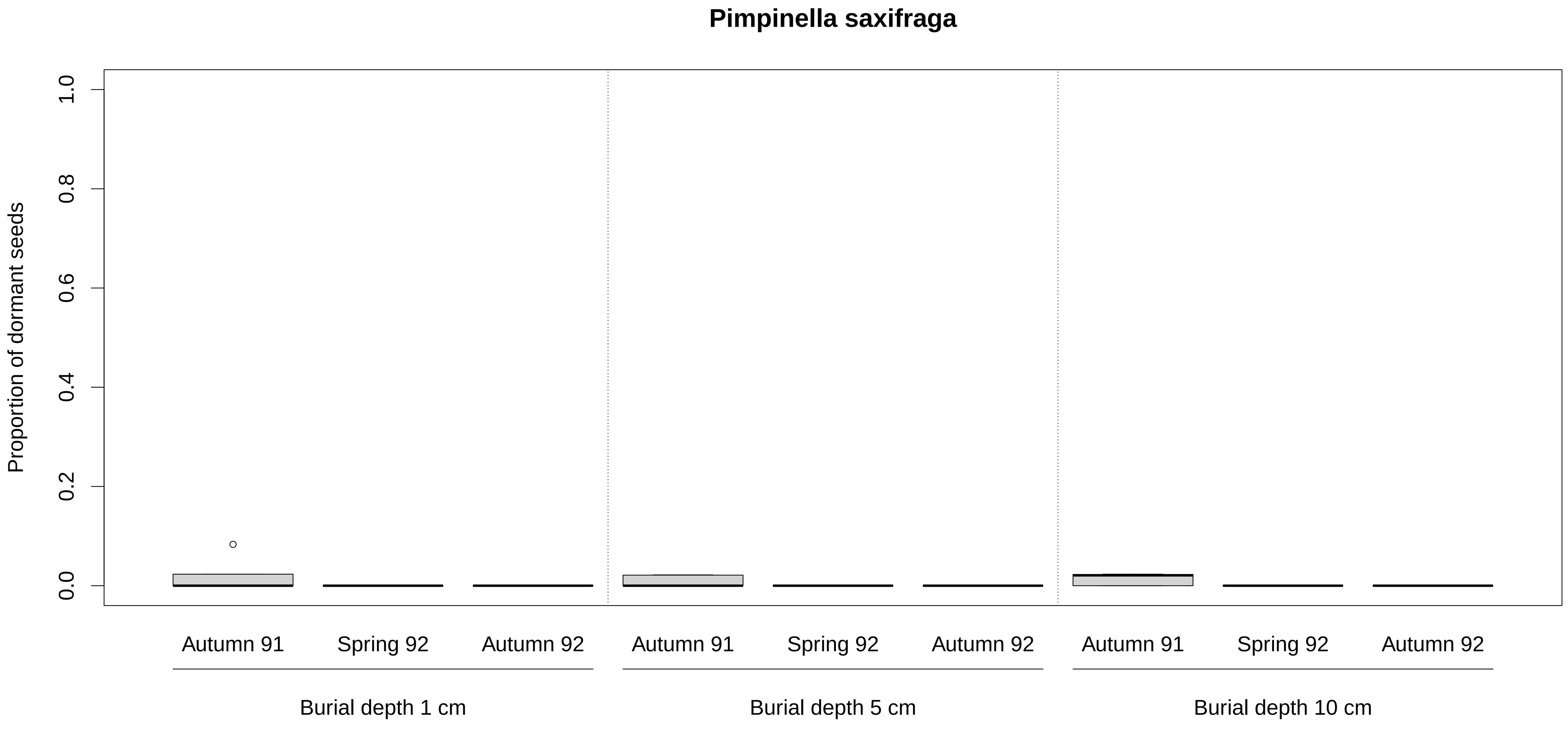

Pimpinella saxifraga
Proportion of dormant seeds
0.0
0.2
0.4
0.6
0.8
1.0
Autumn 91
Spring 92
Autumn 92
Autumn 91
Spring 92
Autumn 92
Autumn 91
Spring 92
Autumn 92
Burial depth 1 cm
Burial depth 5 cm
Burial depth 10 cm

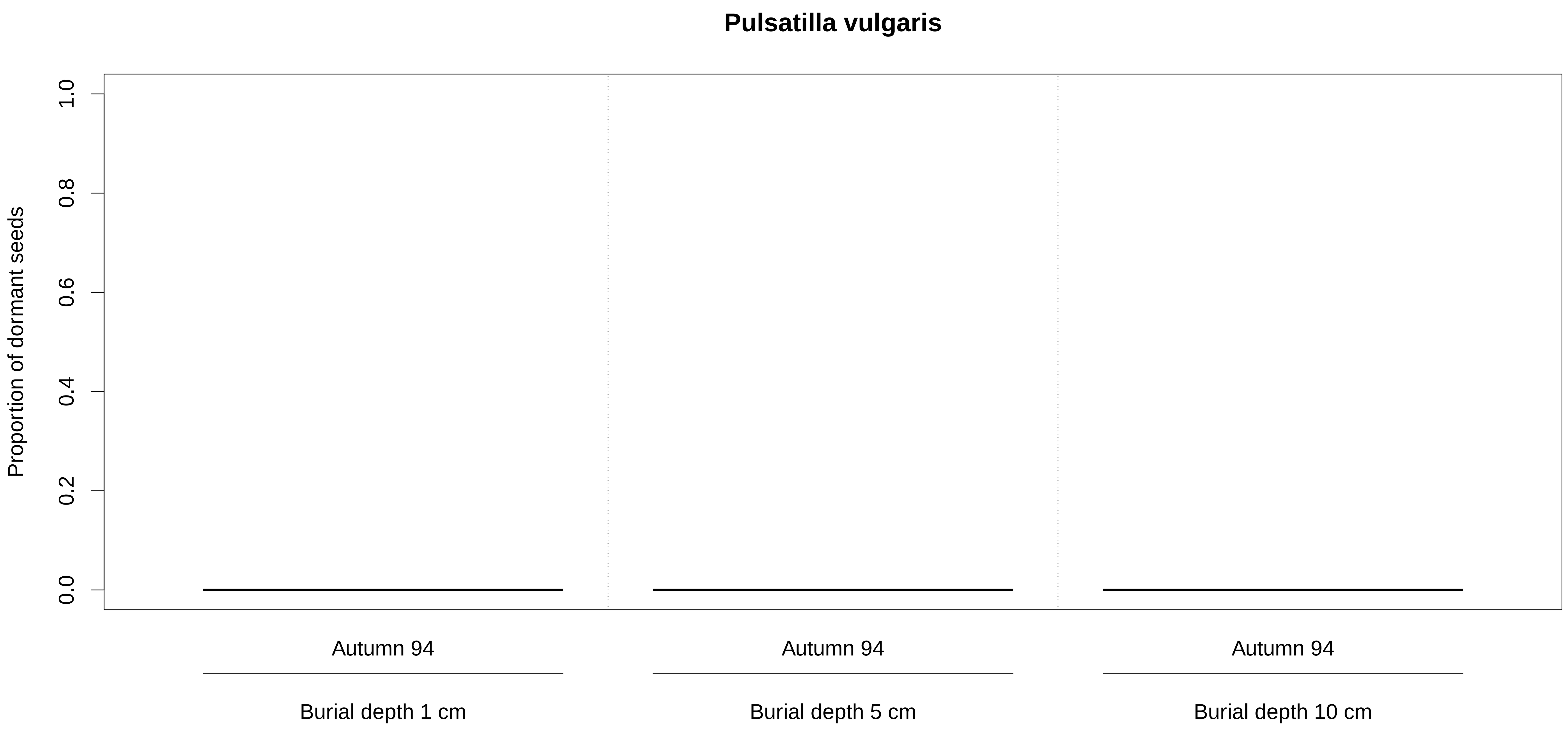
Pulsatilla vulgaris
Proportion of dormant seeds
0.0
0.2
0.4
0.6
0.8
1.0
Autumn 94
Autumn 94
Autumn 94
Burial depth 1 cm
Burial depth 5 cm
Burial depth 10 cm

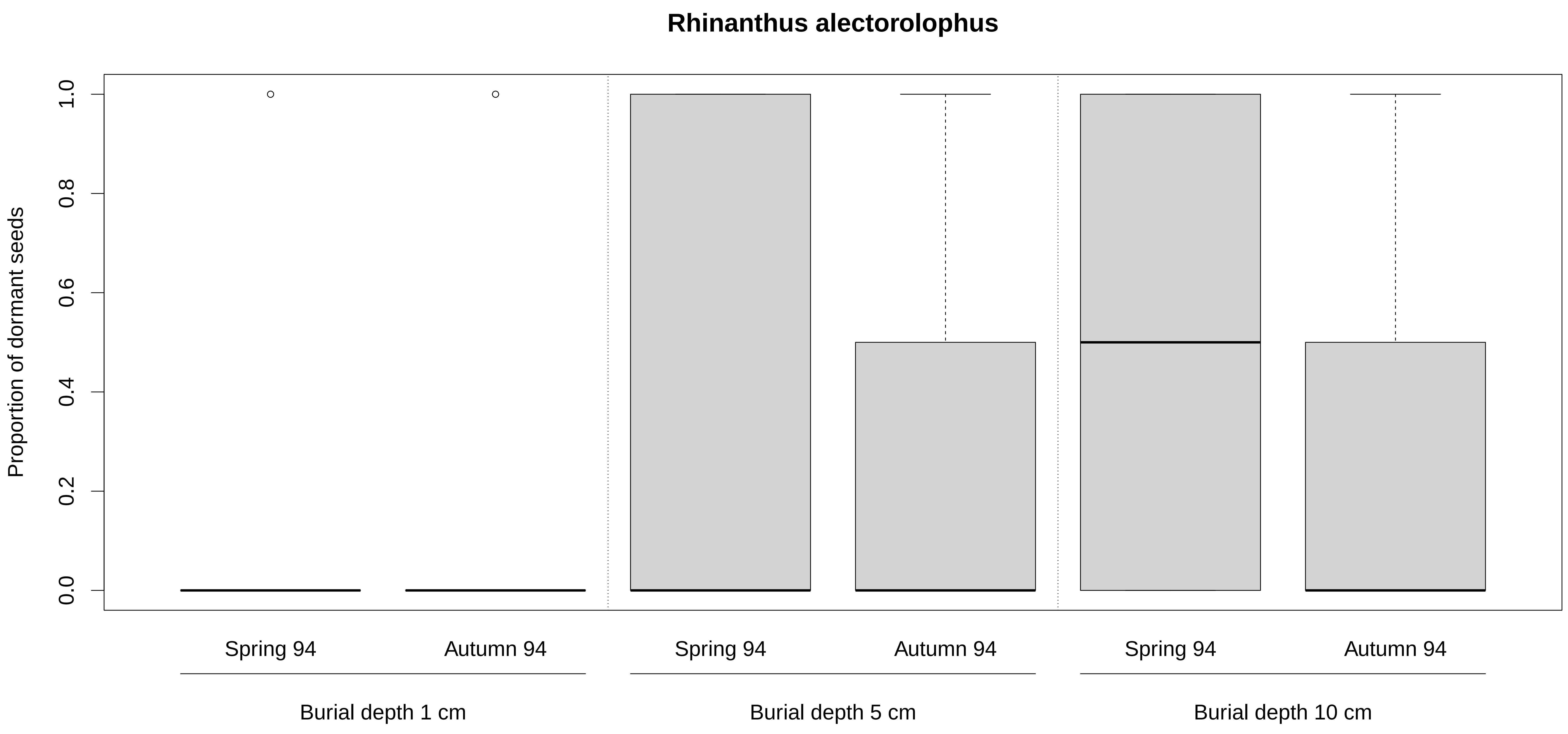
Rhinanthus alectorolophus
Proportion of dormant seeds
0.0
0.2
0.4
0.6
0.8
1.0
Spring 94
Autumn 94
Spring 94
Autumn 94
Spring 94
Autumn 94
Burial depth 1 cm
Burial depth 5 cm
Burial depth 10 cm

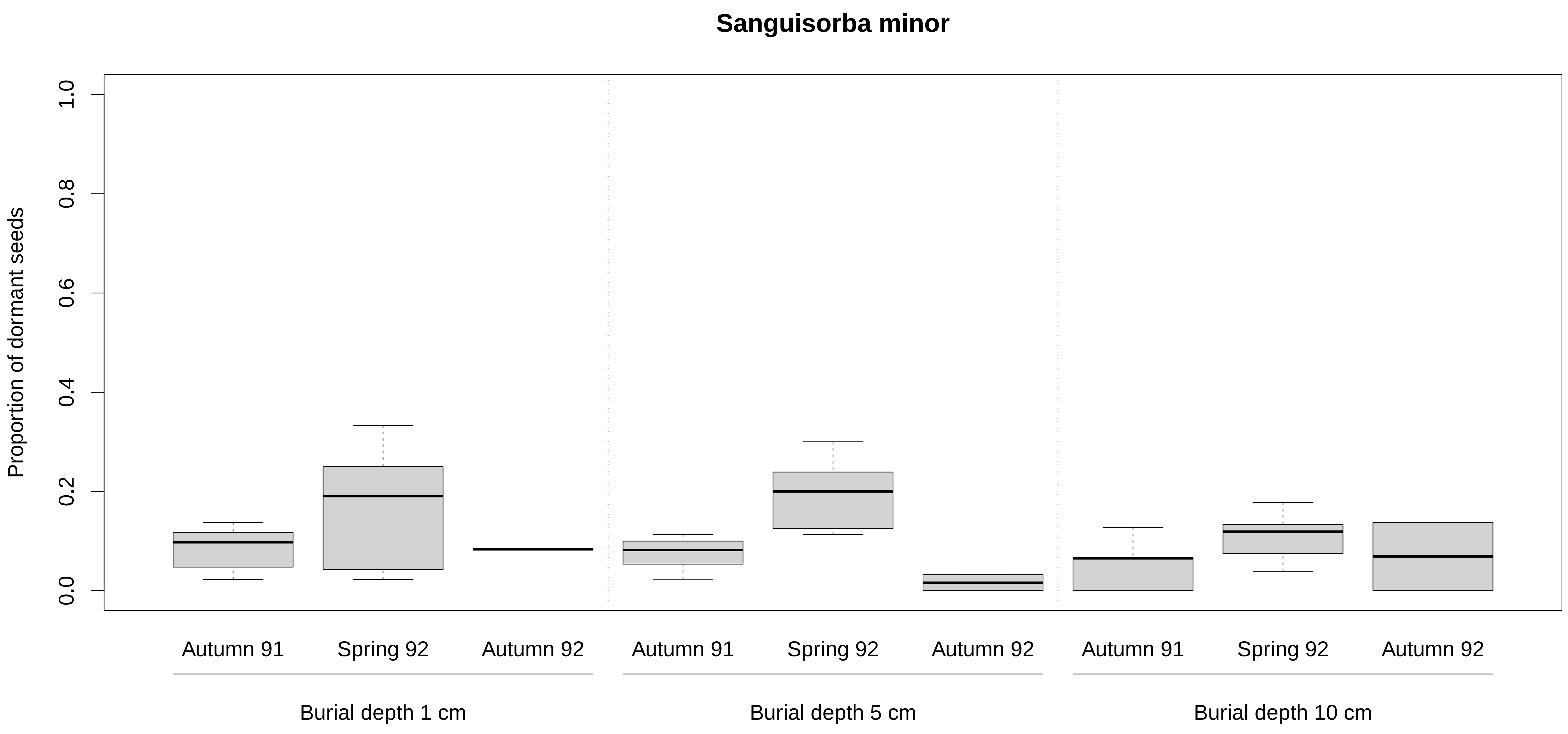
Sanguisorba minor
Proportion of dormant seeds
0.0
0.2
0.4
0.6
0.8
1.0
Autumn 91
Spring 92
Autumn 92
Autumn 91
Spring 92
Autumn 92
Autumn 91
Spring 92
Autumn 92
Burial depth 1 cm
Burial depth 5 cm
Burial depth 10 cm

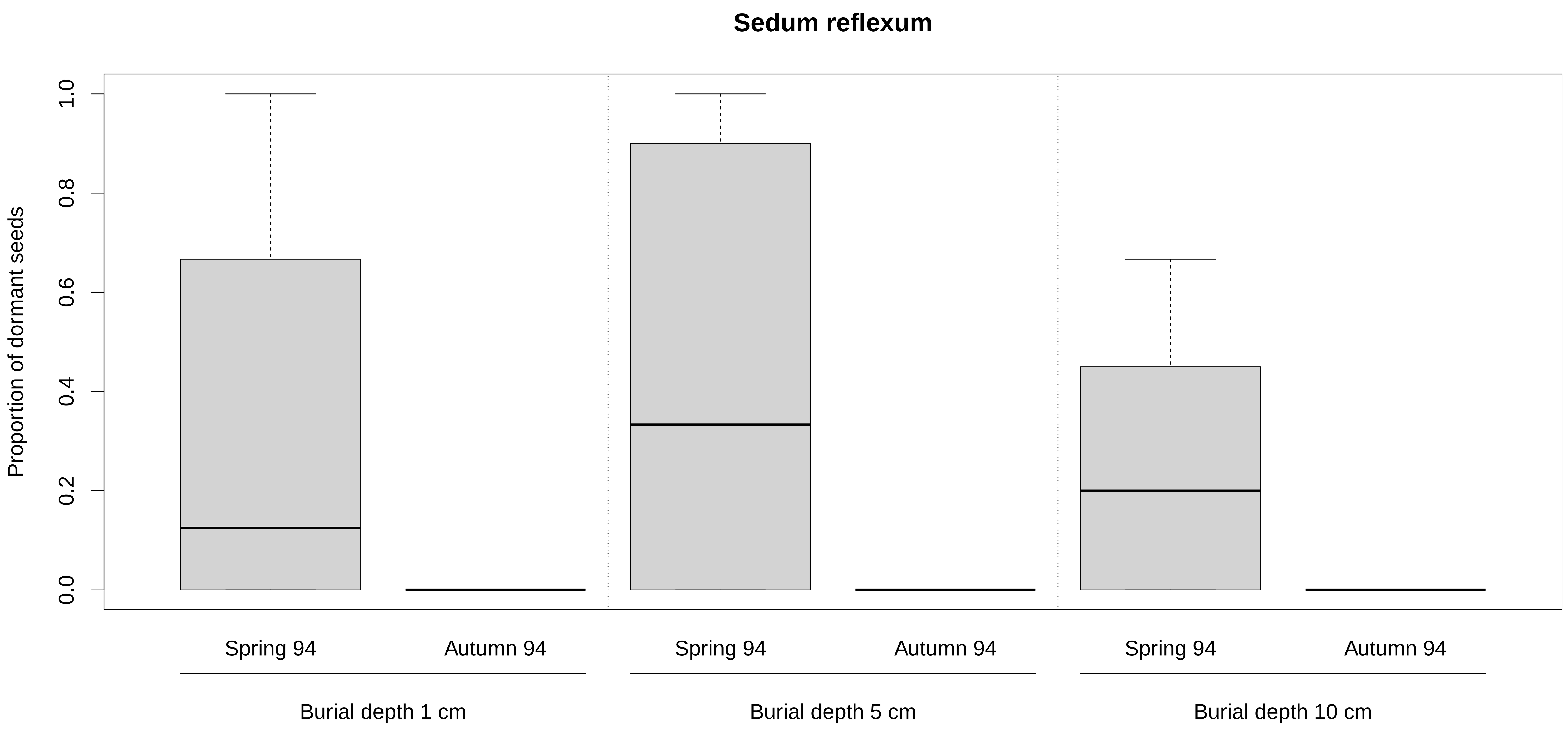

Sedum reflexum
Proportion of dormant seeds
0.0
0.2
0.4
0.6
0.8
1.0
Spring 94
Autumn 94
Spring 94
Autumn 94
Spring 94
Autumn 94
Burial depth 1 cm
Burial depth 5 cm
Burial depth 10 cm
